# Skatole Alleviates Osteoarthritis by Reprogramming Macrophage Polarization and Protecting Chondrocytes

**DOI:** 10.34133/research.0604

**Published:** 2025-02-03

**Authors:** Weiyun Wang, Yaru Chu, Yunkun Lu, Jie Xu, Weixuan Zhao, Zhuo Liang, Xueqiang Guo, Lingling Xi, Tao Han, Yaping Shen, Wenjuan Song, Yanhua Tang, Mengnan Wen, Zhuang Qian, Lei Wang, Zhenlin Fan, Guangdong Zhou, Wenjie Ren

**Affiliations:** ^1^ Institutes of Health Central Plain, Clinical Medical Center of Tissue Engineering and Regeneration, Xinxiang Medical University, Xinxiang 453003, China.; ^2^ The First Affiliated Hospital, Xinxiang Medical University, Xinxiang 453199, China.; ^3^ Department of General Surgery, Sir Run Run Shaw Hospital, Zhejiang University School of Medicine, Hangzhou 310013, China.; ^4^ Shanghai Key Lab of Tissue Engineering, Shanghai 9th People’s Hospital, Shanghai Jiao Tong University School of Medicine, Shanghai 200011, China.

## Abstract

Osteoarthritis (OA) is the most prevalent joint disease, yet effective disease-modifying OA drugs (DMOADs) remain elusive. Targeting macrophage polarization has emerged as a promising avenue for OA treatment. This study identified skatole through high-throughput screening as an efficient modulator of macrophage polarization. In vivo experiments demonstrated that skatole administration markedly reduced synovitis and cartilage damage in both destabilization of medial meniscus (DMM)-induced OA mice and monosodium iodoacetate (MIA)-induced OA rats. Mechanistically, skatole activated signal transducer and activator of transcription 6 (Stat6) signaling, promoting M2 macrophage polarization, while inhibiting nuclear factor-κB (NFκB) and mitogen-activated protein kinase (MAPK) signaling pathways to suppress M1 polarization. RNA-sequencing analysis, targeted metabolomics, and mitochondrial stress tests further revealed that skatole treatment shifted macrophages toward oxidative phosphorylation for energy production. Additionally, it up-regulated genes associated with glutathione metabolism and reactive oxygen species (ROS) pathways, reducing intracellular ROS production. The CUT&Tag assay results indicated that the downstream transcription factor p65 of NFκB can directly bind to gene loci related to inflammation, oxidative phosphorylation, and glutathione metabolism, thereby modulating gene expression. This regulatory process is inhibited by skatole. At the chondrocyte level, conditional medium from skatole-treated M1 macrophages balanced anabolism and catabolism in mouse chondrocytes and inhibited apoptosis. In IL1β-treated chondrocytes, skatole suppressed inflammation and catabolism without affecting apoptosis or anabolism. Overall, skatole maintains immune microenvironment homeostasis by modulating macrophage polarization in joints and preserves cartilage function by balancing chondrocyte anabolism and catabolism, effectively alleviating OA. These findings suggest skatole’s potential as a DMOAD.

## Introduction

Osteoarthritis (OA) is a degenerative joint disease that substantially impacts patients’ quality of life and results in an enormous socioeconomic burden. Typical features of OA include progressive cartilage degeneration, joint pain, and synovitis [1]. Current treatments for early OA typically involve oral nonsteroidal anti-inflammatory drugs (NSAIDs) or injections of glucocorticoids and sodium hyaluronate, which primarily alleviate pain but do not halt further joint structure deterioration and often lead to side effects. Ultimately, many OA patients require costly joint replacement surgery [2–4]. However, there is currently a lack of disease-modifying OA drugs (DMOADs), underscoring the urgent need for novel treatment approaches.

Traditionally, OA has been perceived as mechanical wear of cartilage. However, increasing studies indicate that low-grade chronic inflammation plays a crucial role in the OA development and joint pain, and preventing synovitis is the key for both OA prevention and treatment [5–7]. Macrophages play a critical role in determining the expression of inflammatory factors during synovial inflammation (synovitis). Within the synovium, macrophages can broadly be categorized as M1 macrophages and M2 macrophages. Activated M1 macrophages produce inflammatory factors such as interleukin-1β (IL1β) and IL6, alongside expressing matrix metalloproteinases (MMPs) and reactive oxygen species (ROS), fostering inflammation, cartilage degradation, and osteophyte formation, thereby exacerbating cartilage tissue. Conversely, M2 macrophages, also known as wound-healing macrophages, confer protective effects on OA by secreting anti-inflammatory factors like IL10 and transforming growth factor β (TGFβ) [8–10]. Studies have demonstrated a positive correlation between the ratio of M1/M2 macrophages and the Kellgren–Lawrence (KL) grade of knee OA patients. Compared to healthy controls, OA patients exhibit higher proportions of M1/M2 macrophages [11]. Promoting M2 polarization and inhibiting M1 polarization can mitigate OA inflammation, ameliorate pathological changes in OA, and retard OA progression [12,13]. Moreover, M1 and M2 polarization dynamically adapt to changes in the microenvironment, with M1 macrophages capable of repolarizing toward M2, underscoring the plasticity of macrophage polarization [14]. Hence, modulating macrophage M1/M2 polarization during OA presents a promising therapeutic avenue. We hypothesized that small molecules targeting macrophage polarization may be candidates for DMOADs.

Traditional Chinese medicine (TCM) boasts a history spanning thousands of years and has found wide-ranging clinical applications [15–17]. TCM monomers, active compounds from Chinese herbal medicine, have been recognized for various medicinal properties, including anti-oxidant, anti-apoptotic, anti-bacterial, and anti-inflammatory effects [18–20]. Numerous anti-inflammatory TCM monomers, such as artemisinin, have gained global recognition and serve as valuable resources for novel drug development [21]. In this study, we screened the Traditional Chinese Medicine Active Compound Library (MCE, HY-L065) and identified skatole as capable of promoting macrophage M2 polarization and inhibiting M1 polarization. Skatole, a microbial derivative of tryptophan and an aryl hydrocarbon receptor (AhR) ligand, is among the most malodorous compounds found in fecal matter. Despite its unpleasant odor, skatole has been found application in the perfume industry and has even been used as a flavor additive in ice cream [22,23]. Studies have indicated that skatole can modulate tumor necrosis factor α (TNFα) production and intestinal epithelial cellular functions by activating AhR and the mitogen-activated protein kinase (MAPK) signaling pathway [24,25]. An independent observational study revealed a correlation between gut tryptophan metabolites and symptomatic hand OA (SHOA), with lower skatole level in the plasma of SHOA patients compared to controls [26]. However, there remains limited research on skatole as a treatment for OA.

Here, we demonstrated that skatole regulated macrophage M1–M2 polarization. Further investigation into the molecular mechanisms of skatole revealed its ability to inhibit the nuclear factor-κB (NFκB)/MAPK signaling pathway activation, thereby reducing the release of inflammatory factors and MMPs. Furthermore, we identified that skatole enhanced oxidative phosphorylation (OXPHOS) and glutathione metabolism through the transcription factor p65, a downstream effector of NFκB signaling, thereby altering cellular metabolism and reducing intracellular ROS levels. Moreover, skatole demonstrated the ability to protect chondrocytes by suppressing catabolism and apoptosis and enhancing anabolism, thereby alleviating OA.

## Results

### Skatole promotes M2 macrophage polarization

**Fig. 1.**
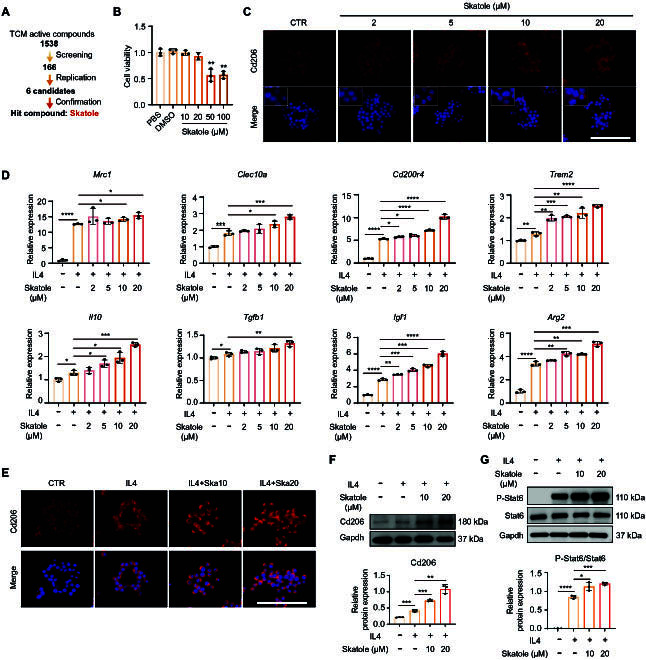
Skatole promotes macrophage M2 polarization. (A) Schematic diagram of high-throughput screening. (B) Cell viability test in macrophages treated with skatole. *n* = 3. (C) Immunofluorescence staining of Cd206 in macrophages treated with skatole. DMSO treatment as the control. The white box represents an enlarged image. Scale bar, 100 μm. (D) RT-qPCR analysis of M2 macrophage marker genes in cells treated with DMSO, IL4, and IL4 + skatole for 48 h. *n* = 3. (E) Immunofluorescence of Cd206 in cells treated with DMSO, IL4, and IL4 + skatole for 48 h. Scale bar, 100 μm. (F and G) Western blotting analysis of Cd206 (F), p-Stat6 (signal transducer and activator of transcription 6), and Stat6 (G) protein levels in cells treated with DMSO, IL4, and IL4 + skatole for 48 h. *n* = 3. *P < 0.05, **P < 0.01, ***P < 0.001, ****P < 0.0001.

Drawing upon our expertise in compound screening for cell fate reprogramming [27–29], we conducted a chemical screen using a TCM active compound library to identify natural compounds capable for promoting M2 macrophage polarization as potential candidates for DMOADs. The RAW264.7 cell line, commonly employed to study macrophage polarization and molecular mechanism, was utilized, with M1/M2 polarization induced by lipopolysaccharide (LPS) and IL4 treatment, respectively. We screened 1,538 TCM monomeric compounds at 5 μM and performed Cd206 immunostaining 48 h later (Fig. S1A to C). Following repeated screening, approximately 50 candidates were identified to promote Cd206 expression (Fig. S1D), some of which have been reported to possess anti-inflammatory effects, such as cardamonin, affirming the efficacy of our screening system [30]. Top 6 compounds were selected for further validation. Reverse transcription quantitative real-time PCR (RT-qPCR) demonstrated that, compared to the other 5 compounds, skatole most significantly increased the expression of *Mrc1* (Cd206) at the mRNA level and markedly inhibited the expression of LPS-induced inflammatory factors such as *Il6* and *Il1b*, suggesting its ability to promote M2 macrophage polarization and inhibit M1 macrophage polarization. Subsequently, we focused our studies on elucidating the roles of skatole in macrophage polarization (Fig. S1E and Fig. 1A).

Initially, we determined the optimal concentration of skatole and found that concentrations below 50 μM effectively promoted Cd206 expression in a concentration-dependent manner (Fig. 1B and C). In IL4-induced macrophage M2 polarization, skatole also demonstrated significant promoting effects. Skatole transcriptionally up-regulated M2 macrophage-related genes, including surface marker genes (*Mrc1*, *Clec10a*, *Cd200r4*, and *Trem2*) and anti-inflammatory factor genes (*Il10*, *Tgfb1*, *Igf1*, and *Arg2*) (Fig. 1D). In bone marrow-derived macrophages (BMDMs), skatole at concentrations of 50 to 200 μM similarly promoted the expression of genes associated with M2 macrophages (Fig. S2A and B). At the protein level, skatole significantly increased Cd206 expression (Fig. 1E and F). The Janus kinase/signal transducer and activator of transcription (JAK/STAT) pathway, a crucial intracellular signaling pathway, plays a pivotal role in inflammation and immune responses [31]. STAT6, a key transcription factor in M2 polarization, regulates the production of anti-inflammatory factors. We observed that skatole increased IL4-induced STAT6 phosphorylation (Fig. 1G). In summary, we identified a TCM monomer, skatole, capable of promoting M2 polarization from M0 macrophage and IL4-induced macrophages.

### Skatole promotes the polarization of macrophages from M1 to M2

**Fig. 2.**
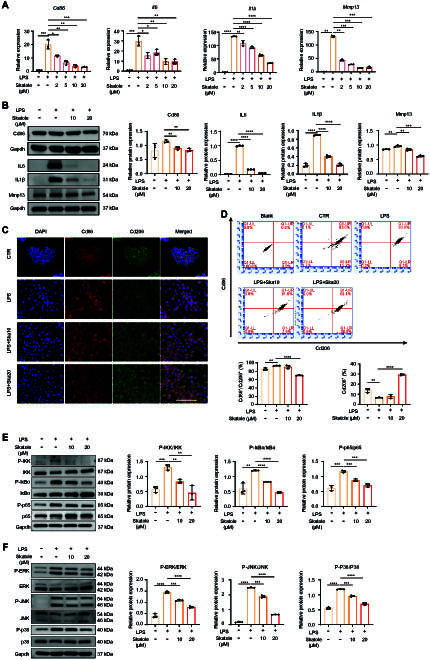
Skatole inhibits LPS-induced M1 macrophage polarization and increases M2/M1 macrophage ratio. (A) RT-qPCR analysis of the gene expression of Cd86, *Il6*, *Il1b*, and *Mmp13* in RAW264.7 cells treated with DMSO, LPS, and LPS + skatole for 24 h. *n* = 3. (B) Western blotting analysis of Cd86, IL6, IL1β, and Mmp13 protein levels in DMSO-, LPS-, and LPS + skatole-treated cells at 24 h. *n* = 3. (C) Co-immunofluorescence of Cd86 and Cd206 in DMSO-, LPS-, and LPS + skatole-treated cells at 24 h. Scale bar, 100 μm. (D) Flow cytometry analysis of Cd86 and Cd206 in macrophages treated with DMSO, LPS, and LPS + skatole for 24 h. *n* = 3. (E and F) Western blotting analysis of proteins in NFκB (E) and MAPK (F) signaling in RAW264.7 cells treated with DMSO, LPS, and LPS + skatole for 12 h. *n* = 3. *P < 0.05, **P < 0.01, ***P < 0.001, ****P < 0.0001.

Next, we further explored the effect of skatole on M1 macrophage polarization. In LPS-induced M1 macrophages, skatole notably reduced the expression of inflammatory factors Il6 and Il1β, and M1 surface marker Cd86 at both the mRNA and protein levels (Fig. 2A and B). Furthermore, skatole inhibited the expression of Mmp13, suggesting its potential to impede the degradation of chondrocyte extracellular matrix in an inflammatory environment. We also detected that skatole reduced the expression of inflammatory cytokines and *Mmp13* in BMDM cells (Fig. S2C). The co-immunofluorescence staining results showed that, compared to the control group, LPS-induced M1 macrophages expressed high levels of Cd86 and low levels of Cd206. Skatole treatment inhibited the expression of Cd86 and promoted the expression of Cd206 (Fig. 2C). Similar to the co-immunofluorescence staining results, flow cytometry showed that macrophages expressed both CD86 and CD206. LPS treatment decreased the number of CD206^+^ cells while increasing the number of CD86^+^/CD206^+^ cells. In contrast, skatole treatment significantly increased the number of CD206^+^ cells and reduced the number of CD86^+^/CD206^+^ cells, further indicating that skatole promotes macrophage repolarization from M1 to M2 (Fig. 2D). NFκB and MAPK signaling pathways play pivotal roles in regulating macrophage M1 polarization and the expression of genes associated with immune response and oxidative stress [32,33]. However, previous studies have reported that skatole can act as a MAPK activator, enhancing the expressions of p-p38, p-ERK (extracellular signal–regulated kinase), and p-JNK (c-Jun N-terminal kinase), and inducing the expression of inflammatory factors [24,25]. This inconsistency with the anti-inflammatory effects demonstrated in our study prompted further investigation. Moreover, there is a lack of studies reporting the regulatory effects of skatole on the NFκB signaling pathway. Therefore, our next step is to examine whether and how skatole regulates the NFκB/MAPK signaling pathways during macrophage polarization.

RAW264.7 macrophages were treated with LPS or LPS + skatole for 12 h and harvested for subsequent analysis. Western blot analysis revealed that skatole increased the expression but inhibited the phosphorylation of NFκB cascade signaling regulated by LPS, including upstream signals IκB kinase (IKK) and IκBα, and downstream transcription factor p65 (Fig. 2E). Additionally, we observed that LPS activated MAPK signaling, whereas skatole inhibited the phosphorylation of ERK, JNK, and p38 (Fig. 2F). These results suggested that skatole inhibits the activation of the nuclear factor κB (NF-κB)/MAPK signaling pathways, thereby suppressing M1 macrophage polarization.

We selected natural compounds reported in recent years that regulate macrophage polarization to alleviate OA by inhibiting the NFκB/MAPK signaling pathways [19,34,35], and tested them simultaneously with skatole (Fig. S3). The RT-qPCR results showed that 4 compounds exhibited the ability to inhibit M1 macrophage polarization and promote M2 polarization, although their effects varied. Among them, skatole demonstrated a stronger anti-inflammatory effect compared to the other compounds, while its ability to promote M2 polarization was similar to that of the other compounds. This suggests that skatole has potential therapeutic value for OA.

### Skatole regulates macrophage immune response

To further elucidate the molecular mechanisms underlying skatole’s effects on macrophage polarization, RAW264.7 macrophages treated with dimethyl sulfoxide (DMSO) (CTR), LPS, and skatole supplemented with LPS (LPS + Ska) for 24 h were subjected to RNA-sequencing (RNA-seq) analysis. We conducted differential expression analysis between the last 2 groups, identifying 505 up-regulated genes and 470 down-regulated genes after skatole treatment (Fig. S4A and B). Principal components analysis (PCA) demonstrated that DMSO-treated cells were transcriptionally similar to LPS + Ska-treated cells than to LPS-treated cells (Fig. S4C). Next, Kyoto Encyclopedia of Genes and Genomes (KEGG) pathway enrichment analysis revealed down-regulated genes associated with c-type lectin receptor signaling pathway, rheumatoid arthritis, TNF signaling pathway, Toll-like receptor signaling pathway, and cytokine–cytokine receptor interaction (Fig. S4D). These down-regulated genes included inflammatory factors such as *Il6*, *Il1a*, *Il1b*, and *Ccl5*, enzyme coding genes such as *Mmp9*, *Mapk11*, and *Mapk12*, and transcription factors such as *Stat1*, *Stat2*, *Nfatc1*, and *Nfatc2* (Fig. S4E). Gene Ontology (GO) analysis indicated down-regulated terms related to immune responds, including positive regulation of cytokine production, immune system development, response to virus, and response to cytokine (Fig. S4F).

### Skatole enhances glutathione metabolism and suppresses ROS production

**Fig. 3.**
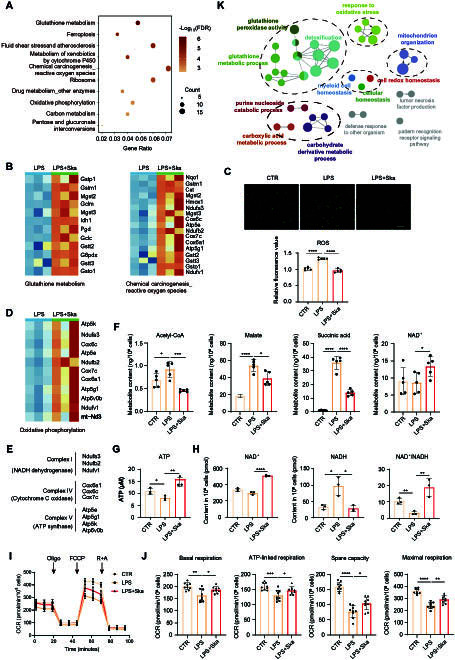
Skatole alters macrophage metabolism and regulates ROS production. (A) KEGG pathway enrichment of up-regulated genes in cells treated with LPS + skatole versus LPS. (B) Up-regulated genes involved in the terms of Glutathione metabolism and Chemical carcinogenesis_ reactive oxygen species in LPS + skatole-treated macrophages versus LPS-treated macrophages. (C) Fluorescence staining and quantification of ROS in cells treated with DMSO, LPS, and LPS + skatole for 24 h. Scale bar, 100 μm. *n* = 5. (D) Up-regulated genes involved in OXPHOS in LPS + skatole-treated macrophages versus LPS-treated macrophages. (E) Up-regulated genes involved in OXPHOS belong to respiratory chain complex I, IV, and V. (F) Metabolite content of acetyl-CoA, malate, succinic acid, and NAD^+^ in macrophages treated with DMSO, LPS, and LPS + Ska. *n* = 5. (G and H) Levels of ATP, NAD^+^, and NADH, as well as the ratio of NAD^+^/NADH in samples treated with DMSO, LPS, and LPS + Ska. *n* = 3. (I) Quantification of OCR of macrophages treated with DMSO, LPS, and LPS + Ska. (J) Indicated OCR parameters. *n* = 9. (K) Functional enrichment analysis of up-regulated genes in cells treated with LPS + skatole versus LPS. *P < 0.05, **P < 0.01, ***P < 0.001, ****P < 0.0001.

M1 macrophage activation is usually accompanied by the production of ROS. LPS and other pro-inflammatory factors can activate macrophages and promote ROS production. ROS can further enhance the expression of pro-inflammatory cytokines such as TNFα, IL1β, and IL6, which in turn exacerbates the inflammatory response [36,37]. Moreover, ROS production promotes cartilage matrix degradation and inhibits cartilage matrix synthesis, aggravating OA progression [38,39]. Scavenging synovial ROS and reactive nitrogen species (RNS) can promote macrophage M2 repolarization and modulate joint inflammation, thereby treating OA [40,41]. Glutathione (GSH) is a crucial substrate for antioxidant enzymes and an important anti-inflammatory agent. GSH and glutamate cysteine ligase (Gcl) are down-regulated in LPS-induced macrophage M1 polarization [42,43]. Our analysis revealed up-regulated genes associated with glutathione metabolism and chemical carcinogenesis_reactive oxygen species after skatole treatment. Notably, genes coding the catalytic (*Gclc*) and modifier (*gclm*) subunit of Gcl, as well as glutathione *S*-transferase family genes (*Gstp1*, *Gstm1*, *Gsto1*, *Gstt2*, and *Gstt3*), were up-regulated (Fig. 3A and B). The above results suggested that skatole may regulate cellular redox homeostasis by modulating glutathione metabolism. In addition, antioxidant coding genes *Nqo1* and *Cat* were up-regulated (Fig. 3B). Then, we detected the production of ROS after skatole treatment in M1 macrophages. As expected, skatole reduced the ROS level increased by LPS (Fig. 3C).

### Skatole promotes OXPHOS and increased ATP generation

Furthermore, skatole up-regulated the expression of OXPHOS-related genes, including NADH [reduced form of nicotinamide adenine dinucleotide (oxidized form) (NAD^+^)] dehydrogenase (mitochondrial complex I) coding genes *Ndufa3*, *Ndufb2*, and *Ndufv1*, cytochrome C oxidase (mitochondrial complex IV) coding genes *Cox6c*, *Cox7c*, and *Cox6a1*, and adenosine triphosphate (ATP) synthase (mitochondrial complex V) coding genes *Atp5k*, *Atp5e*, and *Atp5g1* (Fig. 3D and E). To further verify the enhancement of OXPHOS by skatole, we conducted targeted metabolomics to measure the levels of metabolites related to central carbon metabolism. The results showed that after skatole treatment, the levels of NAD^+^, a product of the electron transport chain, increased, while the levels of succinate, a substrate of the electron transport chain, as well as acetyl-CoA (coenzyme A) and malate, which are intermediate metabolites of the tricarboxylic acid (TCA) cycle, decreased (Fig. 3F and Fig. S5A and B). In addition, we used assay kits to measure intracellular ATP, NAD^+^, and NADH levels. After skatole treatment, intracellular ATP levels and NAD^+^ content significantly increased, while NADH, a substrate of the electron transport chain, decreased, leading to an elevated NAD^+^/NADH ratio (Fig. 3G and H). The oxygen consumption rate (OCR) results similarly showed that skatole treatment partially restored the basal respiration, maximal respiration, ATP-linked respiration, and spare capacity that were reduced by LPS (Fig. 3I and J and Fig. S5C and D). In summary, skatole promotes macrophage M1-to-M2 polarization by enhancing OXPHOS and ATP production.

GO analysis showed up-regulated terms related to glutathione metabolic process, response to oxidative stress, mitochondrion organization, carbohydrate derivative metabolic, and detoxification (Fig. 3K).

Overall, these findings suggested that skatole modulated the metabolic profile of macrophages.

### Skatole can transcriptionally regulate genes involved in inflammatory response, glutathione metabolism, and OXPHOS through p65 protein

**Fig. 4.**
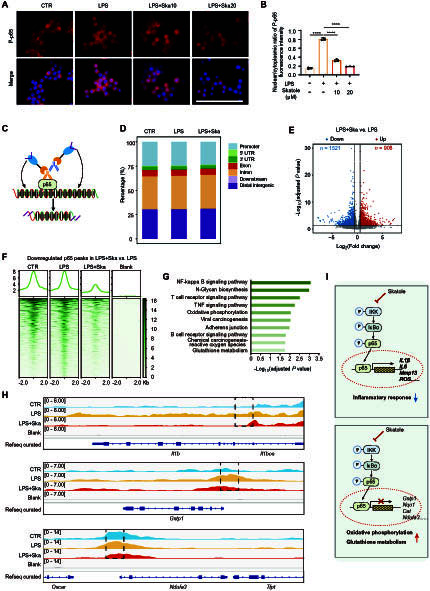
Skatole inhibits p65 nuclear localization and transcriptionally regulates genes involved in inflammatory response, OXPHOS, and glutathione metabolism. (A and B) Immunofluorescence of p-p65 in DMSO-, LPS-, and LPS + skatole-treated cells at 12 h and statistics on the rate of p65 nuclear translocation. Scale bar, 100 μm. *n* = 3. (C) Schematic of CUT&Tag experiments to map the genomic occupancy of p65. (D) Distribution of p65 peaks in the genome of macrophages treated with DMSO, LPS, and LPS + skatole for 12 h. (E) Volcano plot of differential p65 peaks in cells treated with LPS and LPS + skatole. Red dots represent up-regulated peaks, and blue dots represent down-regulated peaks in LPS + skatole-treated cells. Dashed lines represent 1.5-fold change, adjusted *P* value < 0.05. (F) Down-regulated p65 peaks in LPS + skatole sample versus LPS sample. (G) KEGG analysis of down-regulated p65 peak-related genes in LPS + skatole sample versus LPS sample. (H) p65 occupancy at *Il1b*, *Gstp1*, and *Ndufa3* gene loci in cells treated with DMSO, LPS, LPS + skatole, and no-antibody control. (I) Schematic diagram showing that skatole inhibits p65 nuclear import, and p65 positively regulates inflammation-related genes and negatively regulates genes involved in OXPHOS and glutathione metabolism.

The p65 protein, downstream of the NFκB signaling pathway, is an important transcription factor that regulates the secretion of inflammatory factors and macrophage polarization. We previously found that skatole can inhibit the activation of the NFκB signaling pathway (Fig. 2E). Immunofluorescence staining showed that skatole can inhibit the nuclear translocation of p65 (Fig. 4A and B). Therefore, we speculate whether skatole transcriptionally regulates the expression of inflammation-related genes and cellular metabolism-related genes through p65.

To verify this hypothesis, we conducted CUT&Tag (cleavage under targets and tagmentation) assay, a novel method used to study protein–DNA interactions [44] (Fig. 4C). High-throughput sequencing analysis showed no significant difference in the genomic distribution of p65 peaks among the 3 groups of samples (Fig. 4D). Compared to the LPS group, skatole resulted in 908 up-regulated p65 peaks and 1,521 down-regulated p65 peaks (Fig. 4E). Given the reduced nuclear translocation of p65 associated with skatole treatment, we conducted KEGG analysis on the genes corresponding to the down-regulated peak sites. The results indicated that these genes are involved in various signaling pathways, including inflammation-related signaling pathways, OXPHOS, and glutathione metabolism (Fig. 4F and G). Notable genes identified include Il1b, Gstp1, and Ndufa3, among others (Fig. 4H). Based on the previous experimental results, we concluded that P65 can directly bind and transcriptionally promote inflammation-related genes while transcriptionally suppressing the expression of OXPHOS and glutathione metabolism-related genes. This regulatory effect was inhibited by skatole, thereby suppressing macrophage M1 polarization (Fig. 4I).

### Skatole inhibits macrophage conditional culture medium-induced chondrocyte damage

**Fig. 5.**
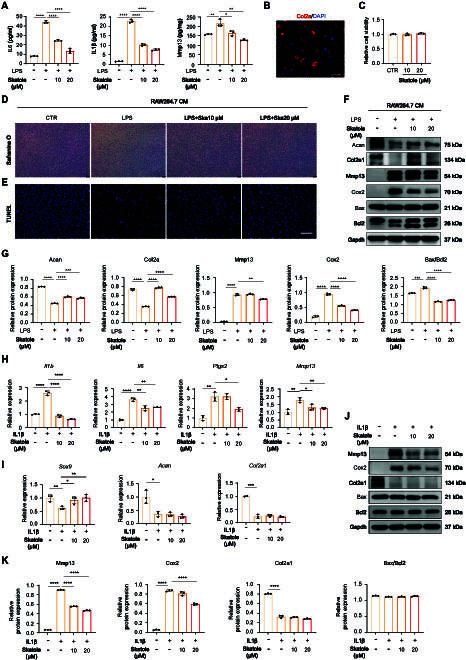
Skatole-treated macrophage CM and skatole small-molecule inhibits chondrocyte apoptosis, inflammation, and catabolism and promotes chondrocyte anabolism. (A) ELISA to detect the secretion of IL6, IL1β, and Mmp13 in LPS- and LPS + skatole-treated macrophage CM. *n* = 3. (B) Immunofluorescence staining of Col2a1 in primary chondrocytes. Scale bar,100 μm. (C) Cell viability of chondrocytes treated with or without skatole. *n* = 3. (D and E) Safranin O staining (D) and TUNEL staining (E) of chondrocytes treated with macrophage CM for 48 h. Scale bar, 100 μm. (F and G) Western blotting analysis of Acan, Col2a1, Mmp13, Cox2, Bax, and Bcl2 protein levels in chondrocytes treated with macrophage CM for 48 h. *n* = 3. (H and I) RT-qPCR analysis of *Il1b*, *Il6*, *Ptgs2*, *Mmp13* (H), *Sox9*, *Acan*, and *Col2a1* (I) gene expression in chondrocytes treated with DMSO, IL1β, and IL1β + skatole for 24 h. *n* = 3. (J and K) Western blotting analysis of Mmp13, Cox2, Col2a1, Bax, and Bcl2 protein levels in chondrocytes treated with DMSO, IL1β, and IL1β + skatole for 24 h. *n* = 3. *P < 0.05, **P < 0.01, ***P < 0.001, ****P < 0.0001.

In OA joint, activated macrophages release inflammatory factors such as cytokines, ROS, and MMPs, leading to chondrocyte apoptosis and disrupting the balance between anabolism and catabolism by degrading native collagen and aggrecan [45]. To investigate the protective effects of skatole, we harvested conditional culture medium (CM) of macrophages treated with LPS and LPS + skatole, and detected the secretion of inflammation factors using an enzyme-linked immunosorbent assay (ELISA). The result revealed that LPS stimulation induced high expression of IL6, IL1β, and Mmp13, while skatole treatment significantly down-regulated the secretion of these factors (Fig. 5A). Next, we treated mouse chondrocytes with the macrophage CM. Primary chondrocytes were identified by immunofluorescence for Col2a1, and skatole treatment did not affect chondrocyte viability (Fig. 5B and C). After 48 h of treatment, LPS-CM reduced the number of chondrocytes, which was partially restored by LPS + skatole-CM in a dose-dependent manner (Fig. 5D). TUNEL (terminal deoxynucleotidyl transferase–mediated deoxyuridine triphosphate nick end labeling) staining showed that LPS-CM induced cell apoptosis, which was inhibited by LPS + skatole-CM (Fig. 5E). Moreover, the anti-apoptosis-related marker Bcl2 was up-regulated (Fig. 5F and G). The above results indicated that LPS + skatole-CM inhibited chondrocyte apoptosis.

Furthermore, coculture with LPS-CM increased cartilage matrix degrading protein Mmp13 expression and reduced the expression of Acan and Col2a1, main components of extracellular matrix of chondrocytes. High expression of Mmp13 further promoted extracellular matrix degradation. LPS + skatole-CM reduced the level of Mmp13 and improved the expression of Acan and Col2a1. Additionally, the expression of cyclooxygenase 2 (Cox2), which has a pro-inflammatory effect, significantly decreased with LPS + skatole-CM (Fig. 5F and G). Similar results were observed at the transcriptional level in the ATDC5 cell line, where LPS + skatole-CM treatment resulted in decreased expression of *Mmp13* and *Ptgs2* (Cox2), and increased expression of *Col2a1* and *Sox9* (Fig. S6).

Overall, LPS + skatole-CM inhibited the expression of inflammation factors, MMPs, and the ratio of Bax/Bcl2 in chondrocytes, thereby protecting chondrocytes from apoptosis and extracellular matrix degradation. The CM increased the intrinsic expression of Acan and Col2a1, maintaining a balance between catabolism and anabolism of chondrocytes.

### Skatole inhibits IL1β-induced inflammation and extracellular matrix degradation in chondrocytes

IL1β plays a pivotal role in the pathophysiology of OA, as chondrocytes treated with IL1β release inflammatory mediators and MMPs, leading to altered chondrocyte function and further degradation of the cartilage matrix [46,47]. To further investigate the direct effect of skatole on chondrocytes, we treated mouse chondrocytes with IL1β in the presence or absence of skatole. Skatole inhibited the expression of inflammation-related genes induced by IL1β, including *Il1b*, *Il6*, and *Ptgs2*, and *Mmp13* expression was also repressed by skatole (Fig. 5H). Interestingly, skatole promoted the expression of *Sox9*, a gene related to chondrocyte differentiation and matrix synthesis, but had no effect on the expression of *Acan* and *Col2a1* (Fig. 5I). These results were consistent at the protein level, with Mmp13 and Cox2 notably down-regulated by skatole compared to IL1β-treated cells, while the protein level of Col2a1 was not restored (Fig. 5J and K). Moreover, IL1β and skatole had no effect on the expression of Bax and Bcl (Fig. 5J and K). Therefore, these experiments indicate that skatole can inhibit IL1β-induced inflammation and extracellular matrix degradation in chondrocytes, but had no effect on apoptosis and anabolism of chondrocytes.

### Intra-articular injection of skatole reduces DMM-induced synovitis

**Fig. 6.**
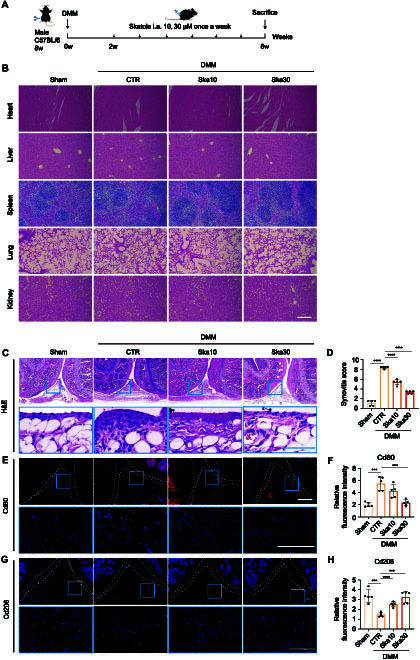
Skatole regulates the polarization of synovial macrophages and reduces synovitis in DMM-induced OA mice. (A) Schematic diagram of the experimental design for intra-articular injection of skatole in a DMM-induced OA mouse model. (B) H&E staining sections of major organs. Scale bar, 100 μm. (C) H&E staining of synovial tissue. The portion encircled in blue in the above image is magnified further below. Scale bar, 100 μm. (D) Histopathologic scoring of synovial tissue. *n* = 5. (E) Immunofluorescence staining of Cd80. The portion encircled in blue in the above image is magnified further below. Scale bar, 100 μm. (F) Quantitative analysis of Cd80 immunofluorescence intensity. *n* = 5. (G) Immunofluorescence staining of Cd206. The portion encircled in blue in the above image is magnified further below. Scale bar, 100 μm. (H) Quantitative analysis of Cd206 immunofluorescence intensity. *n* = 5. ***P < 0.001, ****P < 0.0001.

Next, we assessed the role of skatole in macrophage polarization and synovitis in vivo. Destabilization of medial meniscus (DMM)-induced OA mice were treated with intra-articular injection of skatole at doses of 10 and 30 μM from week 2 after operation. After 6 treatments, the mice were sacrificed (Fig. 6A). Examination of the heart, liver, spleen, lung, and kidney structure revealed that DMM and skatole treatment did not affect these organs (Fig. 6B). Macrophage infiltration in the synovium plays an essential role in triggering inflammation in OA. We evaluated the infiltration and phenotypic characterization of macrophages in the synovium. Hematoxylin and eosin (H&E) staining demonstrated that, in the control group, cell infiltration led to a marked increase in synovial thickness, whereas both low and high doses of skatole ameliorated macrophage infiltration and synovial thickness (Fig. 6C). Synovitis scores corroborated these findings (Fig. 6D). Cd80 is a surface marker for M1 macrophages [48,49], and RT-qPCR result showed that Cd80 is highly expressed in M1 macrophages, with skatole treatment significantly reducing the expression (Fig. S7). Immunofluorescence staining revealed a significant increase in Cd80-positive cells and a decrease in Cd206-positive cells in the synovial tissue of the DMM group, indicating an elevated M1/M2 macrophage ratio, consistent with previous reports [9,10]. Skatole dose-dependently reduced the number of Cd80-positive cells and increased Cd206-positive cells (Fig. 6E to H). These results confirmed that skatole increased the ratio of M2/M1 macrophages and alleviated synovitis.

### Skatole alleviates structural degeneration and modulates the balance of catabolism and anabolism of articular cartilage in DMM-induced OA mice

**Fig. 7.**
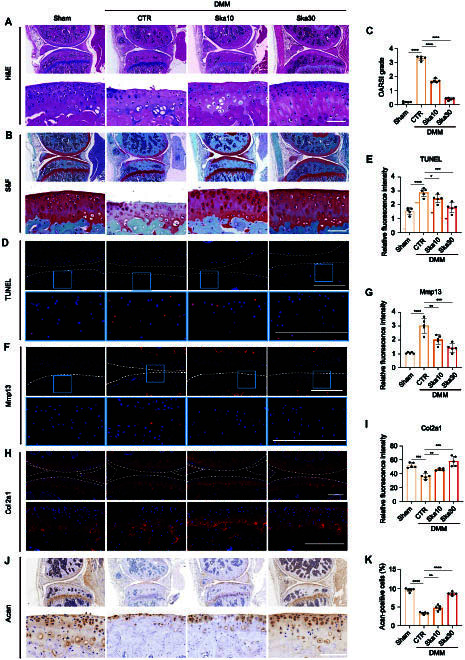
Skatole suppresses apoptosis of articular chondrocytes and maintains cartilage morphology in DMM-induced OA mice. (A) H&E staining of the joint tissue after 8 weeks of DMM surgery. The overall view and enlarged image. Scale bar, 100 μm. (B) Safranin O and Fast Green staining of the joint tissue after 8 weeks of DMM surgery. The overall view and enlarged image. Scale bar, 100 μm. (C) Quantitative analysis of OARSI scores. *n* = 5. (D) TUNEL staining of the joint tissue after 8 weeks of DMM surgery. The portion encircled in blue in the above image is magnified further below. Scale bar, 100 μm. (E) Quantitative analysis of TUNEL immunofluorescence intensity. *n* = 5. (F) Immunofluorescence staining of Mmp13 in the joint tissue after 8 weeks of DMM surgery. The portion encircled in blue in the above image is magnified further below. Scale bar, 100 μm. (G) Quantitative analysis of Mmp13 immunofluorescence intensity. *n* = 5. (H) Immunofluorescence staining of Col2a1 in the joint tissue after 8 weeks of DMM surgery. The overall view and enlarged image. Scale bar, 100 μm. (I) Quantitative analysis of Col2a1 immunofluorescence intensity. *n* = 5. (J) Immunohistochemistry of Acan in the joint tissue after 8 weeks of DMM surgery. The overall view and enlarged image. Scale bar, 100 μm. (K) Statistical results of Acan-positive cell rate. *n* = 5. *P < 0.05, **P < 0.01, ***P < 0.001, ****P < 0.0001.

To further confirm that skatole could reduce cartilage destruction, mouse joints were sectioned for histological analysis at week 8 after operation. H&E and Safranin O and fast green (S&F) staining demonstrated reduced proteoglycans and exacerbated cartilage surface erosion in the control group. In contrast, the degeneration of cartilage was markedly inhibited in skatole-treated DMM-induced OA mice (Fig. 7A to C). Similarly, TUNEL staining results showed that skatole treatment reduced the cell apoptosis rate induced by DMM (Fig. 7D and E). Additionally, compared to the control group, skatole treatment resulted in a reduction in cartilage degradation, as evidenced by immunofluorescence and immunohistochemical staining, indicating a decrease in the expression of the matrix proteinase Mmp13 (Fig. 7F and G), while the expression of Col2a1and Acan was restored (Fig. 7H to K).

### Skatole mitigates synovial inflammation and cartilage damage in MIA-induced OA rats

We also established a monosodium iodoacetate (MIA)-induced OA rat model to evaluate the therapeutic effects of skatole on synovial inflammation and articular cartilage damage. A single injection of 1 mg of MIA was administered into the joints of Sprague–Dawley (SD) rats, followed by a treatment injection of skatole (30 μM) the following day. The drug was administered every 2 d, and the joints were assessed 1 and 2 weeks after treatment (Fig. S8A). H&E staining showed remarkable synovial hyperplasia 1 week after MIA injection, and synovitis persisted 2 weeks later. Skatole treatment inhibited synovial inflammation (Fig. S8B and C). The results of H&E staining and S&F staining indicated that 1 week after modeling, there was mild damage to the articular cartilage, and the therapeutic effect of skatole was not significant. However, 2 weeks after modeling, the articular cartilage exhibited more severe degeneration, while treatment with skatole markedly inhibited this degeneration in the OA rats. (Fig. S8D and E).

## Discussion

In this study, through chemical screening and experimental verification, we identified skatole as a promising compound capable of modulating macrophage repolarization and attenuating the progression of OA, thus providing a potential candidate drug for DMOADs. The therapeutic effects of skatole in other inflammatory disease models beyond OA merit further investigation. We also conducted in vitro and in vivo experiments to validate other candidate compounds, among which there were also compounds with significant therapeutic effects (the results are not shown). In the future, we will combine these compounds to treat OA and search for the optimal treatment strategy.

After further mechanistic investigations, we have identified a previously undiscovered connection between skatole and NFκB/MAPK signaling pathway. Our experiments demonstrated that skatole can inhibit the phosphorylation of IKK, IκBα, and p65, which has not been reported in literature. Skatole can suppress the MAPK signaling pathway by inhibiting the phosphorylation of JNK, ERK, and p38, which contradicts previous reports indicating that skatole activates MAPK to regulate apoptosis [24,25]. These contradictory findings could be attributed to variations in experimental conditions, including skatole concentration and cell types used.

Skatole, a tryptophan microbial derivative, similar to other indole derivatives, can bind and activate AhR, thereby initiating downstream signaling pathways [50]. Consequently, it is plausible that skatole regulates the NFκB/MAPK signaling pathway through AhR, ultimately modulating gene transcription [51,52]. On the other hand, AhR serves as a transcription factor [53], and extensive data suggest that endogenous metabolites can reduce the expression of inflammatory factors by activating AhR [54–57]. We hypothesize that following activation by skatole, AhR may directly bind to the promoter region of the target gene, thereby initiating gene transcription. Further validation of these hypotheses is warranted in future studies.

Further analysis of differentially expressed genes showed that skatole not only inhibits the expression of inflammatory factors but also up-regulates genes involved in cellular metabolism, such as glutathione metabolism, ROS regulation, OXPHOS, and carbon metabolism. Multiple experimental approaches have validated that skatole reduces intracellular ROS levels and promotes the generation of more ATP through OXPHOS. This suggests that as a tryptophan metabolite, skatole also plays a role in cellular metabolism. ‌NFκB signaling is a classic signaling pathway involved in macrophage polarization. Skatole inhibits the activation of NFκB signaling and prevents the nuclear translocation of the downstream transcription factor p65. Is the reduction of inflammatory factor secretion and the increase in expression of glutathione metabolism-related genes and OXPHOS caused by skatole regulated through p65?‌ CUT&Tag assay indicated that p65 directly binds to the loci of the aforementioned genes, and skatole reduces the binding abundance of p65, confirming the above hypothesis. In summary, skatole inhibits inflammatory gene expression and promotes the expression of genes related to OXPHOS and glutathione metabolism by inhibiting the NFκB signaling pathway, thereby regulating macrophage M1–M2 polarization.‌

Additionally, we observed that the conditioned medium from skatole treatment inhibits chondrocyte apoptosis, suppresses extracellular matrix degradation, and promotes anabolism. Direct treatment of chondrocytes with skatole exhibits marked anti-inflammatory effects, which may share the same mechanism as its action in macrophages.

In DMM-induced OA mouse model and MIA-induced OA rat model, skatole treatment effectively reduces synovitis and protects cartilage, thus mitigating the progression of OA. However, in post-traumatic OA models, merely adjusting the immune microenvironment is insufficient to reverse cartilage degeneration. This necessitates a combination with other agents that promote cartilage regeneration, such as drugs, stem cells, or stem cell-derived exosomes. In the advanced stages of OA, where cartilage structure is further compromised, future strategies—beyond traditional joint replacement surgery—may include using biomaterials to support collapsed structures. These biomaterials could be designed to deliver growth factors and immunomodulatory drugs, simulating a regenerative microenvironment and correcting the pathological one, while stem cells could be employed to achieve in situ cartilage regeneration [58,59].

In summary, our research not only provides new strategies for treating OA but also unveils a novel mechanism by which skatole regulates macrophage polarization, offering fresh insights into the role of tryptophan metabolites in modulating inflammatory responses. Moreover, macrophage polarization is crucial in various inflammatory diseases and tumor environments, making it a notable therapeutic target. This suggests that skatole shows promise as a candidate drug for treating a broad spectrum of diseases (Fig. S9).

## Materials and Methods

### Reagents

Information of antibodies, chemical compounds, assay kits, cell and mouse lines, and other reagents is listed in Table S1.

### Mice

All C57BL/6J mice were purchased from SiPeiFu Laboratory Animals (Henan, China). Mice were fed under specific pathogen-free conditions. All animal-related procedures were performed under an approved protocol by Experimental Animal Ethics Committee of Xinxiang Medical University (XYLL-20220079).

### Cell culture

RAW264.7 macrophages and passaged generation of chondrocytes were cultured with high-glucose Dulbecco’s minimum essential medium (DMEM; WISENT, Canada) containing 10% fetal bovine serum (FBS; WISENT, Canada) at 37 °C with 5% CO_2_, and ATDC5 cell line was cultured with low-glucose DMEM (WISENT, Canada) containing 10% FBS. The eighth generation of RAW264.7 macrophages and ATDC5 cell line was used for subsequent progression of the experiment.

### Cell viability

The effect of skatole on cell viability was assessed by MTS kit (Promega, USA). RAW264.7 macrophages (3 × 10^3^ cells/well) and primary chondrocytes (2 × 10^3^ cells/well) were seeded onto 96-well plates and treated with skatole for 48 h. The 100-μl mix containing 90 μl of DMEM and 10 μl of 3-(4,5-dimethylthiazol-2-yl)-5-(3-carboxymethoxyphenyl)-2-(4-sulfophenyl)-2H-tetrazolium (MTS) solution was added to wells. After incubation for 1 to 4 h, the absorbance was measured at 490 nm.

### RAW264.7 macrophage polarization

LPS (100 ng/ml) (Sigma-Aldrich, USA) was used to stimulate M1 polarization, and IL4 (20 ng/ml) (Peprotech, USA) was used to induce M2 polarization. RT-qPCR and immunofluorescence staining were performed to assess macrophage polarization.

### Differentiation and polarization of murine BMDMs

Bone marrow was obtained by flushing the femoral and tibial marrow cavities of C57BL/6J mice. Following centrifugation, the bone marrow cells were resuspended and cultured in RPMI 1640 medium (Gibco, USA) containing 12% FBS and 40 ng/ml of recombinant macrophage colony-stimulating factor (M-CSF) (Sino Biological, China) at 37 °C with 5% CO_2_ for 7 d. LPS (100 ng/ml) and interferon γ (IFNγ) (40 ng/ml) (Sino Biological, China) or IL4 (20 ng/ml) were used to stimulate M1 polarization or M2 polarization.

### Extraction and identification of mouse primary chondrocytes

Rib cartilage was isolated from C57BL/6J suckling mice of 5 to 7 d old and cut into pieces under aseptic conditions. The cartilage was digested with 0.2% collagenase II at 37 °C for 4 h. The digestive fluid was then filtered through a 100-μm cell filter and then centrifuged for 5 min to collect the isolated cells. Primary mouse chondrocytes were cultured in DMEM/F12 (WISENT, Canada) with 10% FBS, which was refreshed every 2 to 3 d. Primary chondrocytes were identified by Col2a1 immunofluorescence staining for subsequent experiments. The second generation of chondrocytes was used for subsequent progression of the experiment.

### Conditioned CM collection and coculture with mouse chondrocytes

LPS and various concentrations of skatole treated RAW264.7 macrophages for 24 h. CM supernatants were collected and filtered. The CM was diluted with DMEM at a 1:1 ratio and added to chondrocytes, which were harvested 48 h later for further analyses.

### RT-qPCR

Total RNAs of macrophages, primary chondrocytes, and ATDC5 cell line were isolated using RNA-Quick Purification Kit (ES Science, China) and reverse transcribed to cDNA by using Hiscript III 1st Strand cDNA Synthesis Kit (Vazyme, China). RT-qPCR was performed using Taq Pro Universal SYBR qPCR Master Mix (Vazyme, China). Data were normalized to glyceraldehyde-3-phosphate dehydrogenase (Gapdh) mRNA levels. The primers used in this study are listed in Table S2.

### Cell immunofluorescence staining

Cd86: Cells were fixed with methanol for 5 min on ice, washed with phosphate-buffered saline (PBS) for 3 times, and blocked with 3% bovine serum albumin (BSA; YEASEN, China) (blocking buffer) for 1 h. Primary antibody (1:200) was diluted in the blocking buffer.

Cd206 and p-p65 cells were fixed with 4% paraformaldehyde (PFA) for 10 min, washed for 3 times, permeated, and blocked with 0.1% Tween-blocking buffer for 30 min. Primary antibody (Cd206, 1:3,000; p-p65, 1:500) was diluted in the blocking buffer.

Col2a1: Cells were fixed with 4% PFA, washed for 3 times, permeated, and blocked with 0.1% Triton-1% BSA for 30 min. Primary antibody (1:100) was diluted in the blocking buffer.

Co-immunofluorescence staining of Cd206 and Cd86: Cells were fixed with methanol for 5 min on ice, washed for 3 times, and blocked with blocking buffer for 30 min. Primary antibody (Cd206, 1:200; Cd86, 1:200) was diluted in 0.1% Tween-blocking buffer.

Cells were incubated with primary antibodies overnight at 4 °C and then washed for 3 times. Cells were then incubated with secondary antibody (goat anti-rabbit, 1:1,000; donkey anti-goat, 1:50) for 1 h at 37 °C. Information of all the antibodies used is listed in Table S1.

### Flow cytometry

RAW264.7 cells (1 × 10^6^) were harvested for flow cytometry. Cd86 antibody (1:200) and Alexa Fluor 555-conjugated goat anti-rabbit immunoglobulin G (IgG) (H&L) were used to mark M1 macrophages, and Cd206 antibody (1:200) and CoraLite488-conjugated goat anti-mouse IgG (H+L) were used to mark M2 macrophages.

### Western blot assay

Cells were lysed on ice for 30 min using radioimmunoprecipitation assay (RIPA) buffer (Beyotime, China), and the total protein was isolated by centrifugation at 10,000*g* × 20 min. Proteins were separated by sodium dodecyl sulfate–polyacrylamide gel electrophoresis and then transferred to a polyvinylidene difluoride membrane (Millipore, USA). The membranes were further blocked with blocking buffer (5% skim milk) and incubated with primary antibodies (Gapdh, 1:5,000; Cd206, STAT6, p-STAT6, Cd86, IL6, IL1β, Mmp13, IκBα, IKK, p65, ERK, JNK, p38, p-IκBα, p-IKK, p-p65, p-ERK, p-JNK, p-p38, Acan, and Cox2, 1:1,000; Col2a1, 1:200; Bax and Bcl2, 1:2,000) overnight at 4 °C. Colors were developed with ECL Basic Plus Kit (Beyotime, China), and signals were measured using a chemiluminescent imaging system. Information of all the antibodies used is listed in Table S1.

### ELISA

RAW264.7 macrophage CM was collected, and the concentrations of IL1β, IL6, and Mmp13 were detected by ELISA kit (IL1β, R&D Systems, USA; IL6 and Mmp13, Proteintech, China) according to the manufacturer’s recommendation.

### ROS detection

The Reactive Oxygen Species Assay Kit (Beyotime, China) was used to measure the intracellular ROS generation. After treatment with LPS and skatole, RAW264.7 macrophages were washed with PBS for 3 times and incubated in DMEM with 10 μM 2',7'-dichlorodihydrofluorescein diacetate (DCFH-DA) for 30 min in the dark at 37 °C. After washing with PBS for 3 times, cells were observed and imaged immediately with inverted fluorescence microscopy (Zeiss). Fluorescence intensity was measured using a microplate reader of multi-wavelength measurement system (Thermo).

### RNA-seq analysis

The total RNA of RAW264.7 macrophages treated with DMSO, LPS, and LPS + Ska for 48 h was extracted. The construction of cDNA library of samples and high-throughput sequencing were completed by Novogene. Sequencing data were analyzed as described previously [27].

### OCR assay

Cells were plated at 8,000 cells per well in an XF24 Cell Culture Miniplate (Agilent Technologies, USA) and incubated overnight. After 24-h treatment, OCR assay was performed in a Seahorse XFe24 Flux Analyzer as previously described [27]. Before detection, CM was changed to XF DMEM supplemented with 2 mM l-glutamine, 1 mM sodium pyruvate, and 10 mM glucose (Sigma-Aldrich, USA). In the OCR assay, cells were stimulated with 1.5 μM oligomycin, 1.5 μM carbonyl cyanide 4-(trifluoromethoxy)phenylhydrazone (FCCP), and 0.5 μM rotenone/antimycin A.

### Targeted metabolomics

About 1 × 10^7^ cells treated with DMSO, LPS, and LPS + Ska for 24 h were harvested and frozen by liquid nitrogen for 15 min. Targeted metabolomics analysis of central carbon metabolism was performed by Novogene based on liquid chromatography–tandem mass spectrometry.

### ATP detection

The intracellular ATP level was measured using Enhanced ATP Assay Kit (Beyotime, China) according to the manufacturer’s instructions. Chemiluminescence intensity was measured with a microplate reader of multi-wavelength measurement system (Thermo).

### NAD^+^/NADH detection

The intracellular NAD content and the total content of NAD and NADH were detected using Enhanced NAD+/NADH Assay Kit with WST-8 (Beyotime, China) according to the manufacturer’s instructions, and the NADH content and NAD/NADH ratio were calculated. Absorbance at 450 nm was measured with a microplate reader of multi-wavelength measurement system (Thermo).

### CUT&Tag assay

CUT&Tag assay was performed using NovoNGS CUT&Tag 4.0 High-Sensitivity Kit (Novoprotein, China) according to the manufacturer’s instructions. Briefly, after treatment with LPS and skatole for 12 h, 2 × 10^5^ cells were harvested and fixed on the surface of concanavalin A (ConA)-coated magnetic beads. Then, cells were incubated with p-p65 antibody (1:50) or without primary antibody (Blank) overnight at 4 °C. Goat anti-rabbit IgG antibody (1:100) was added into the sample and incubated with cells for 1.5 h at room temperature. After washing, ChiTag Transposome was added and incubated with cells for 1 h at room temperature, followed with tagmentation by Tagmentation Buffer. DNA was isolated by Tagment DNA Extract Beads, amplified with N5 and N7 primers, and purified with DNA Clean Beads for high-throughput sequencing. Paired-end sequencing was performed by Novogene.

### DMM-induced mouse model of OA

Surgical destabilization of the medial meniscus (DMM) was used to induce mouse model of OA referring to previous research [60]. Male C57BL/6J mice (8 weeks old, 22 to 24 g) were anesthetized with isoflurane, and the medial meniscus anterior tibial ligament of the right knee joints was incised with a scalpel. The sham operation only opens the joint capsule on the same knee without damaging the ligament or meniscus (*n* = 5). The incision was then closed using sutures. Two weeks after surgery, the DMM mice were randomly divided into 3 groups: DMM, DMM + Ska 10 μM, DMM + Ska 30 μM (*n* = 5 per group). Drugs (saline containing 0.1% DMSO for sham group and DMM group) were injected via intra-articular injection at a dose of 8 μl, once a week, for a total of 6 times. All mice were executed at 8 weeks postoperatively for further evaluation.

### MIA induced rat model of OA

The rat OA model was induced by intra-articular injection of sodium iodoacetate (MIA). Usually, after isoflurane anesthesia, the rat OA model was induced by intra-articular injection of 1 mg of MIA in 8-week-old male SD rats. The day after the OA model construction, rats were randomly divided into 3 groups (*n* = 6): Sham group, MIA group, and MIA + Ska group. Drugs (saline containing 0.1% DMSO for sham group and MIA group, saline containing 30 μM skatole for MIA + Ska group) were injected via intra-articular injection at a dose of 50 μl every 2 d for 1 and 2 weeks.

### Safranin O staining

Safranin O staining was performed using Modified Saffron-O and Fast Green Stain Kit (Solarbio, China) according to the manufacturer’s instruction. After staining, chondrocytes were observed and imaged using a fluorescence microscope.

### TUNEL staining

For CM-treated chondrocytes, TUNEL staining was performed using the In Situ Cell Death Detection Kit (Roche, Switzerland) according to the manufacturer’s instruction. For mouse tissue, cells were deparaffinized and treated with freshly configured TUNEL reaction mixture for 1 h in the dark at 37 °C, then sealed with 4′,6-diamidino-2-phenylindole (DAPI) containing an anti-fluorescence quencher, and observed under a fluorescence microscope (Zeiss). Cells were quantitatively analyzed using ImageJ software.

### Histological analysis

Mice were sacrificed at 8 weeks, and the right joints were fixed in 4% PFA, decalcified and dehydrated, and embedded in paraffin wax. The specimens were sectioned coronal to the specimen into sections of approximately 4-μm thickness for histological and immunohistochemical examination using a sectioning machine (LEIKA). Sections were stained with Cole’s Hematoxylin Solution (Solarbio, China) and Eosin Y Stain Solution (Solarbio, China) (H&E) and Modified Saffron-O and Fast Green Stain Kit (Solarbio, China) (S&F) for further histological analysis according to the manufacturer’s instruction. For H&E staining, the sections were stained in hematoxylin solution for 1 min and then returned to blue in tap water, and incubated in eosin solution for 1 min, rinsed in tap water, and dehydrated and sealed transparently. For S&F staining, the sections were stained with freshly prepared Weigert’s stain for 3 to 5 min, washed with water, immersed in solid green stain for 5 min and in Saffron’s stain for 30 s, and then dehydrated and sealed transparently. Cartilage degeneration was graded using the Osteoarthritis Research Society International (OARSI) score, and synovitis was graded using the synovitis score.

### Immunohistochemical staining

Sections were deparaffinized and antigenically repaired using 0.25% trypsin for 1 h at 37 °C. Endogenous peroxidase activity was stopped with 3% hydrogen peroxide solution, followed by blocking with 5% BSA for 30 min. Sections were incubated with primary antibody (Col2a1, 1:200; Acan, 1:200) overnight at 4 °C and then in secondary antibody (1:1,000) for 1 h at 37 °C. Immunohistochemical staining was performed using 3,3′-diaminobenzidine substrate. Subsequently, hematoxylin restaining was performed, and finally, the sections were dehydrated and transparently sealed.

### Tissue immunofluorescence

Sections were deparaffinized and antigenically repaired using 0.25% trypsin for 1 h at 37 °C and blocked with 5% BSA for 30 min. Cells were incubated with primary antibodies (Mmp13, 1:500; Cd80, 1:500; Cd206, 1:200) overnight at 4 °C, followed by an immunofluorescent secondary antibody (1:250) for 1.5 h at 37 °C. Finally, the sections were sealed with anti-fluorescence quencher containing DAPI. Sections of knee tissues were quantitatively analyzed using ImageJ.

### Statistical analysis

Statistical analysis was performed with GraphPad Prism version 9. All data are presented as the mean ± SD. Statistical significance was assessed by the 2-tailed Student’s *t* test: *P < 0.05, **P < 0.01, ***P < 0.001, ****P < 0.0001.

## Data Availability

The raw data of RNA-seq and CUT&Tag-seq generated in this study have been deposited in the NCBI Gene Expression Omnibus (GEO) database under accession code GSE261400 (RNA-seq) and GSE283748 (CUT&Tag-seq). Data are available from the corresponding author upon request. The original full-length Western blot images are shown in Figs. S10 to S13.
