# Supplementary material for: Skatole Alleviates Osteoarthritis by Reprogramming Macrophage Polarization and Protecting Chondrocytes
Source: Research (Wash D C). 2025 Feb 3;8:0604. doi: 10.34133/research.0604 (PMC11788598; doi:10.34133/research.0604)
Supplement: Supplementary 1 — Figs. S1 to S13 Tables S1 and S2 [file research.0604.f1.zip › Supplemental Materials.docx]

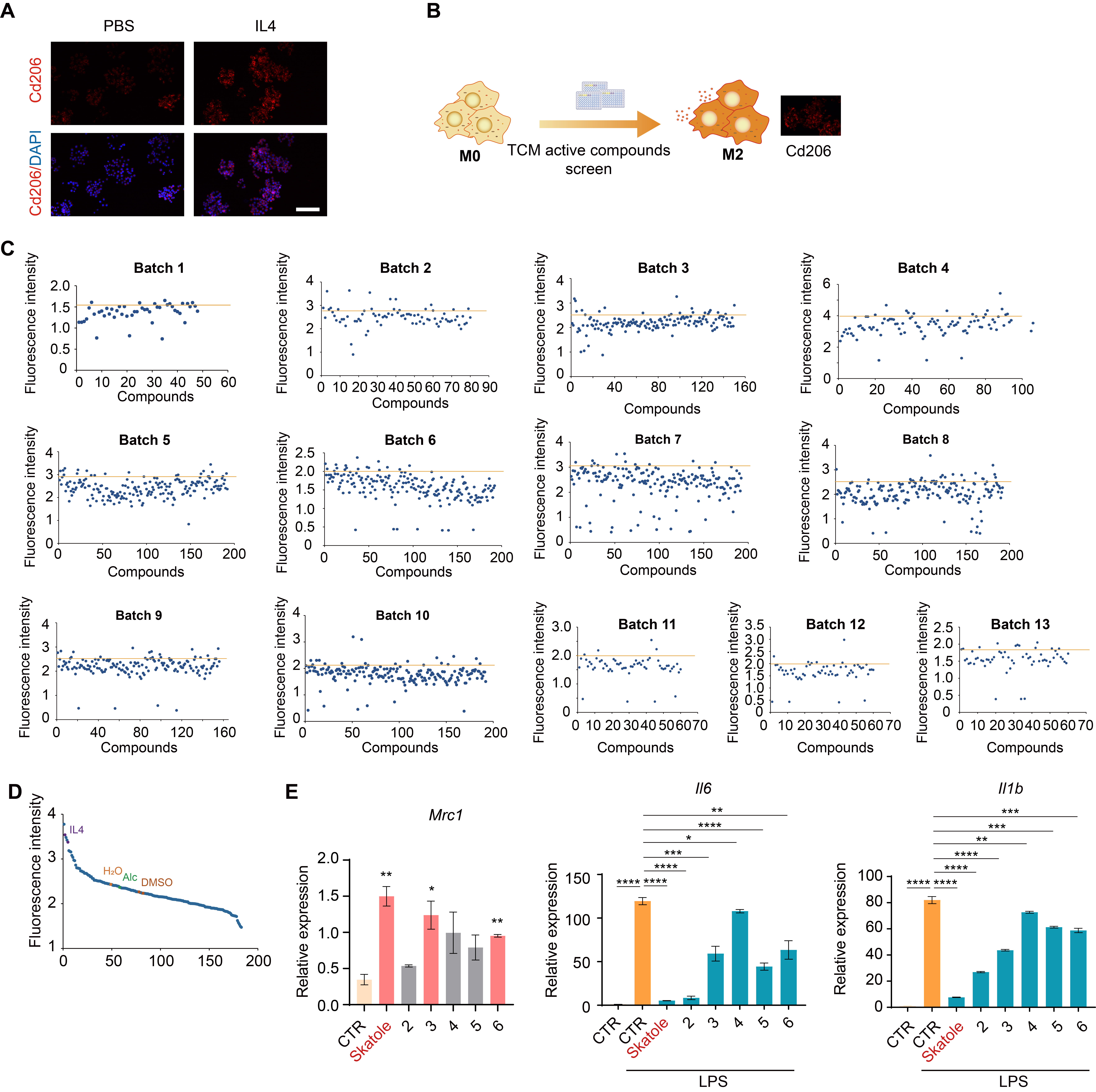


Fig. S1. Traditional Chinese medicine active compounds screen. (A) Immunofluorescence staining of Cd206 in macrophages treated with PBS or IL4. Scale bar, 100 μm. (B) Schematic diagram of compound screen. (C) The compounds were divided into 13 batches for screen. After 48 h, Cd206 immunofluorescence staining was performed, and quantify the fluorescence intensity by ImageJ. The orange line represents a fluorescence intensity 1.5 times higher than that of the control group. The compounds above the orange line were candidates for the second round of screen. (D) Secondary screening of candidate compounds evaluated by Cd206 immunofluorescence at 48 h. (E) RT-qPCR evaluation of Top 6 candidates on the expression of *Cd206* as well as inflammatory factors induced by LPS. Skatole most stably and significantly enhances *Cd206* and inhibits the expression of *Il6* and *Il1b*. n = 3.

*P < 0.05, **P < 0.01, ***P < 0.001, ****P < 0.0001.
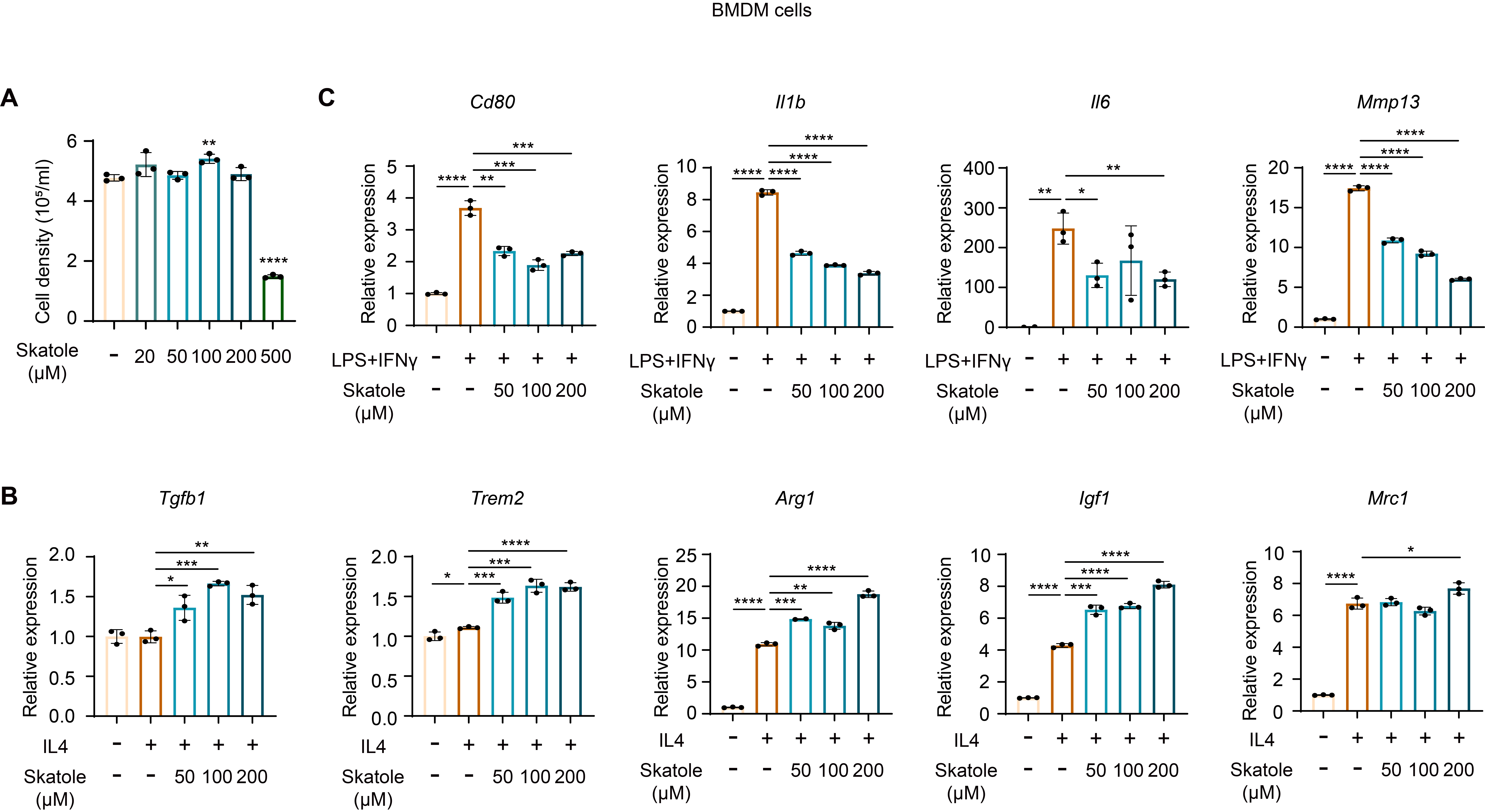


Fig. S2. Skatole promotes M2 polarization and inhibits M1 polarization of bone marrow derived macrophages (BMDMs). (A) The effect of different concentrations of skatole on the density of BMDM cells. n=3. (B) RT–qPCR analysis of *Tgfb1*, *Trem2*, *Arg1*, *Igf1* and *Mrc1* gene expression in BMDM cells treated with IL4 and Skatole. n=3. (C) RT–qPCR analysis of Cd80, Il1b, Il6, and Mmp13 in BMDM cells treated with LPS, IFNγ, and Skatole. n=3.

*P < 0.05, **P < 0.01, ***P < 0.001, ****P < 0.0001.
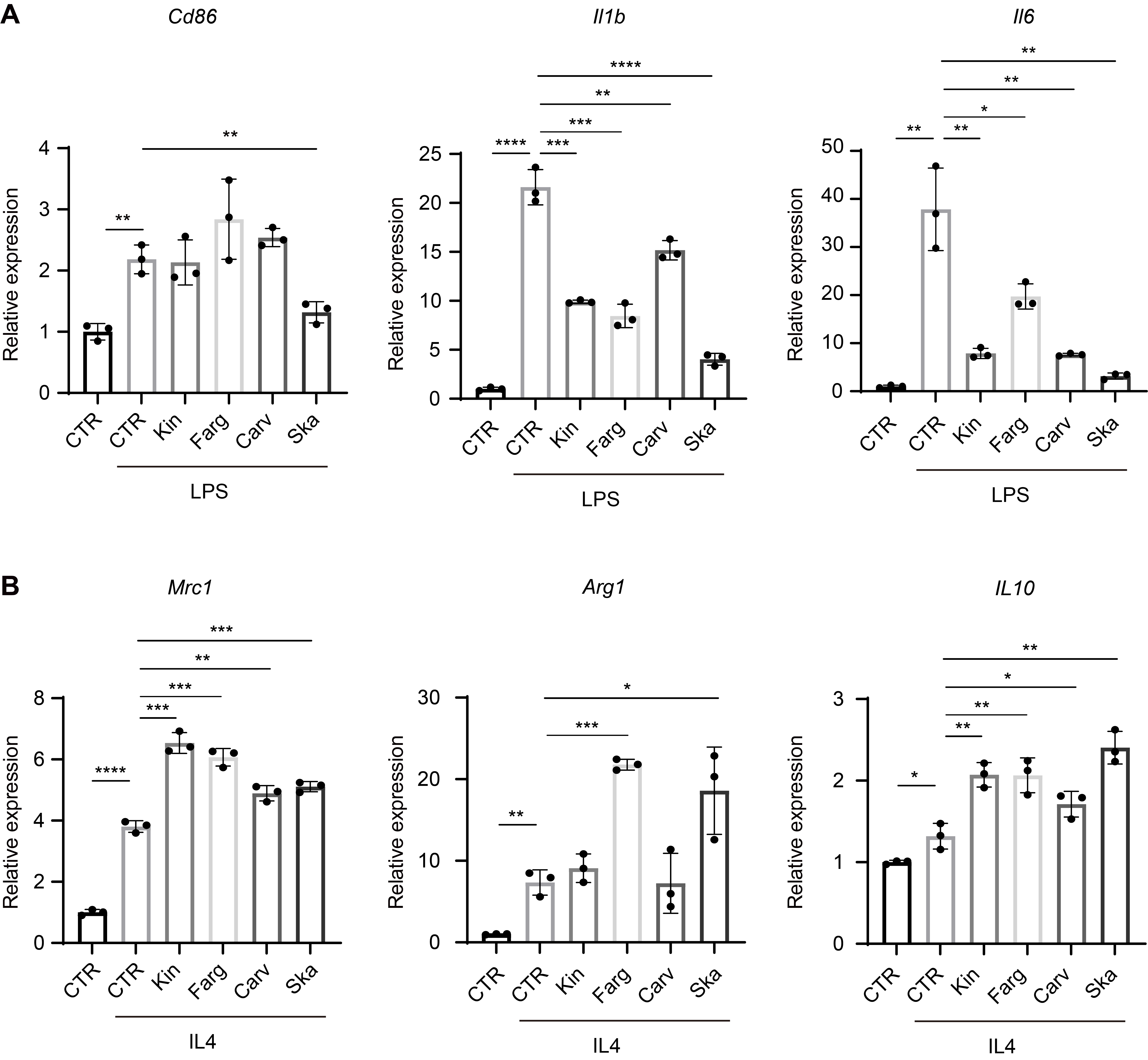


Fig. S3. A comparison of the effects of Skatole and 3 recently reported NFκB/MAPK signaling pathway targeting new drugs on macrophage polarization regulation. RT-qPCR evaluation of 4 drugs on the expression of M1 macrophage markers induced by LPS (A) and the expression of M2 macrophage markers induced by IL4 (B). n=3. Kin: Kinsenoside, 25μg/mL; Farg: Fargesin, 20 μM; Carv: Carveol, 50μg/mL; Ska: Skatole, 20 μM.

*P < 0.05, **P < 0.01, ***P < 0.001, ****P < 0.0001.


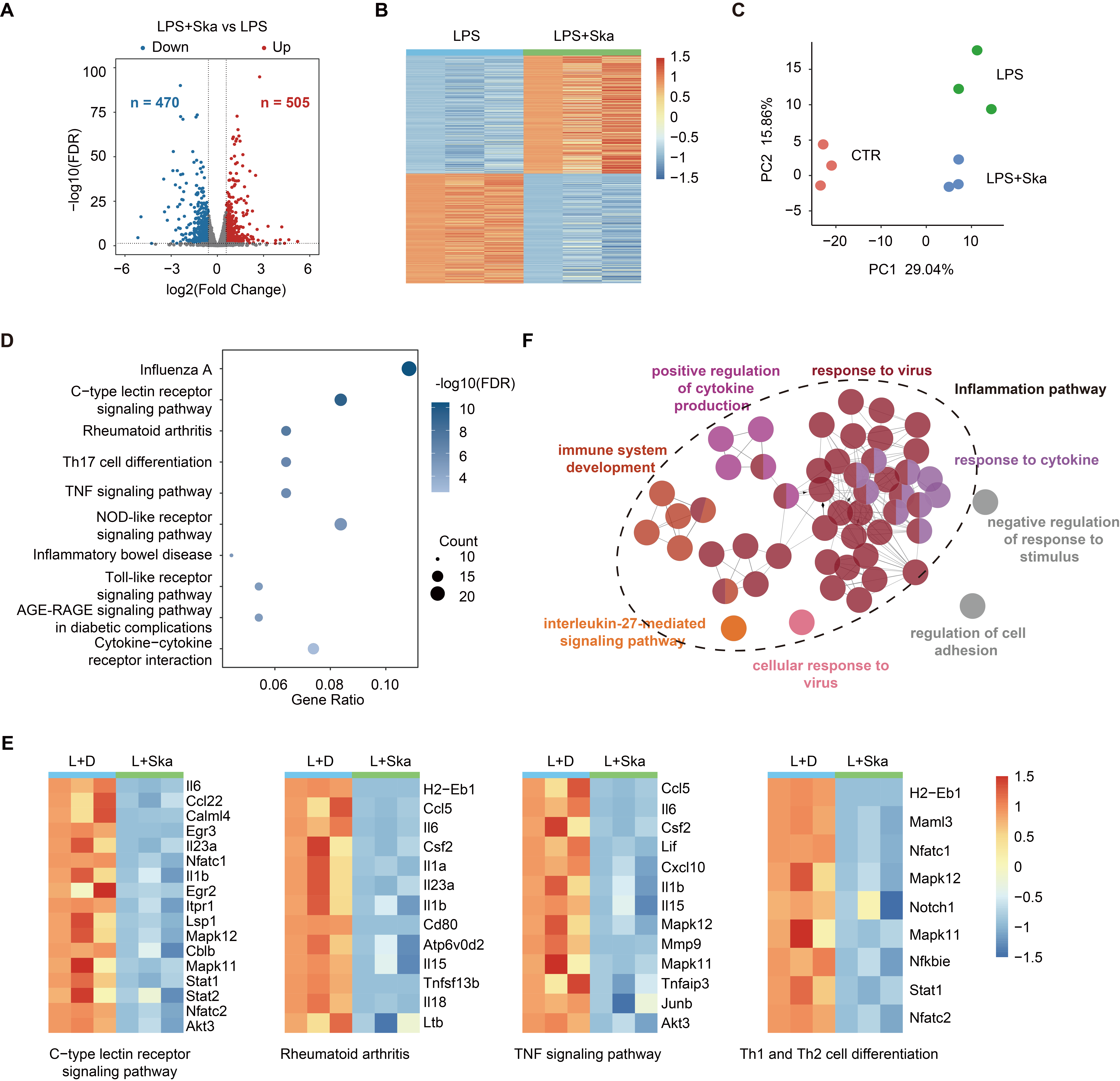


Fig. S4. Skatole regulates the transcriptomic changes in macrophages induced by LPS. (A) Volcano plot of differentially expressed genes (DEGs) in RAW264.7 cells treated with LPS vs. LPS + Skatole for 24 hours. Red dots and blue dots represent upregulated (Fold change > 1.5) and downregulated (Fold change < 0.67) genes. (B) Heatmap of DEGs in RAW264.7 cells treated with LPS vs. LPS + Skatole. (C) Principal component analysis (PCA) of gene expression from DMSO-, LPS- and LPS + Skatole-treated cells. (D) KEGG pathways enriched in down-regulated genes in LPS + Skatole-treated cells vs. LPS-treated cells. (E) Heatmap of downregulated genes involved in the terms of C-type lectin receptor signaling pathway, TNF signaling pathway, Rheumatoid arthritis, and Th1 and Th2 cell differentiation in LPS + Skatole-treated macrophages vs. LPS-treated macrophages. (F) Functional enrichment analysis of down-regulated genes in LPS + Skatole-treated cells vs. LPS-treated cells.


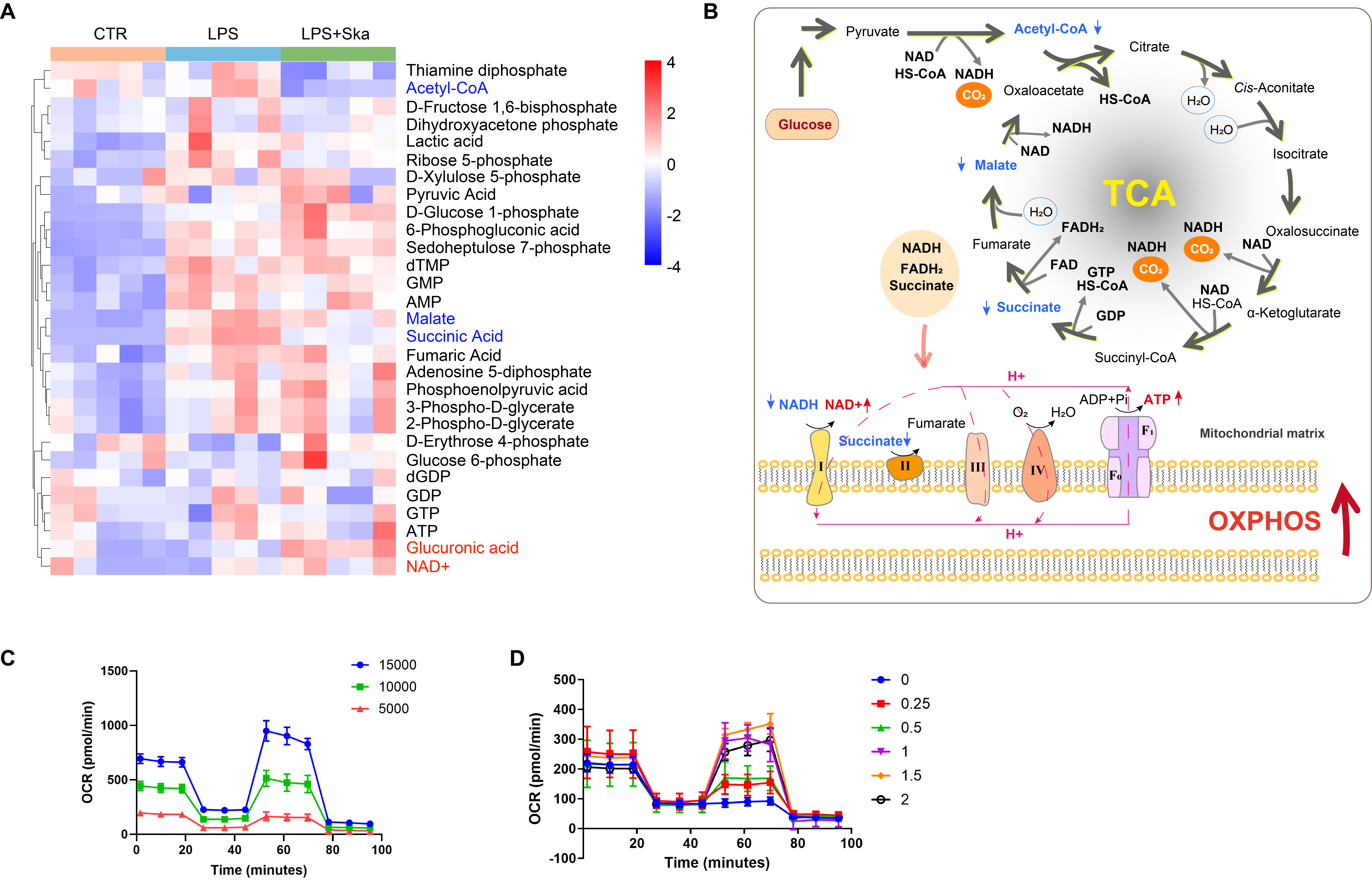
Fig. S5. Skatole modulates cellular metabolism and enhanced cellular oxidative phosphorylation. (A) Heatmap showing the normalized abundance of central carbon metabolites in RAW264.7 cells treated with DMSO, LPS and LPS + Skatole for 24 h. Red indicates upregulated metabolites, whereas blue indicates downregulated metabolites. (B) Schematic diagram showing TCA cycle and OXPHOS in cells treated with Skatole. (C) Optimal cell seeding density test for OCR assay. n=3. (D) Optimal FCCP concentration test for OCR assay. n=3.


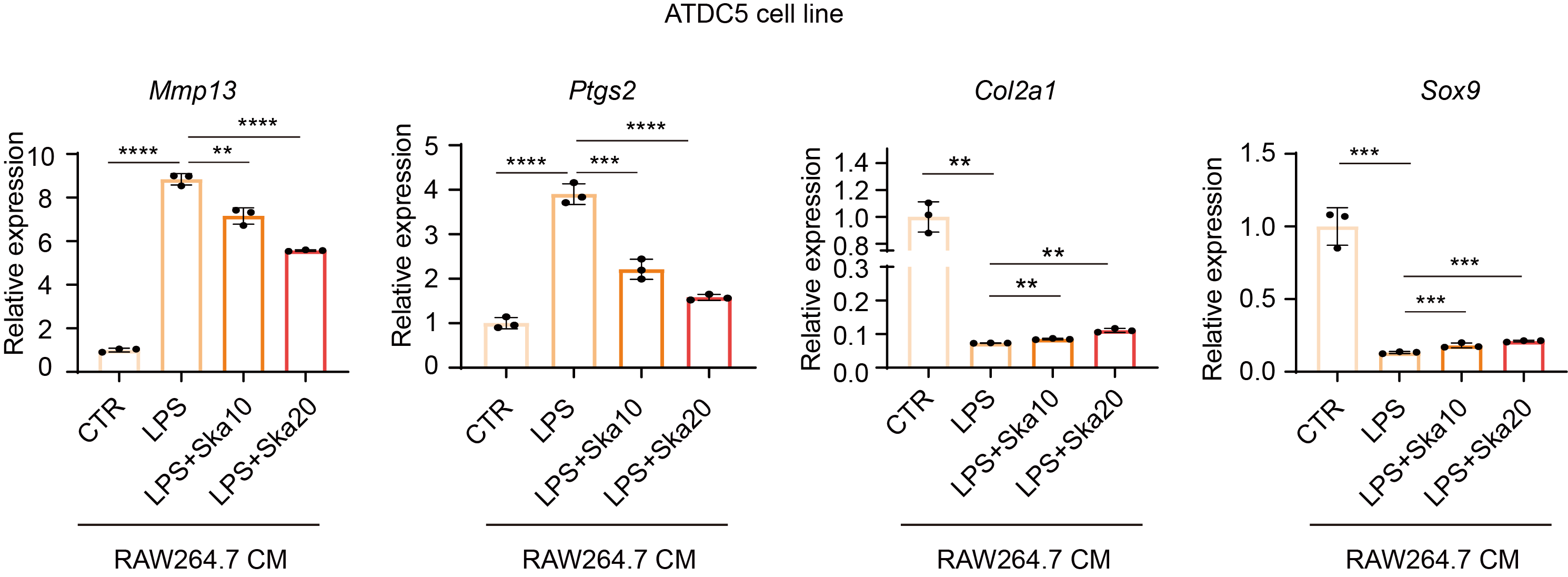


Fig. S6. Skatole treated-macrophage CM inhibits inflammation and catabolism and promotes anabolism in ATDC5 cell line. RT–qPCR analysis of *Mmp13*, *Ptgs2*, *Col2a1*, and *Sox9* gene expression in ATDC5 cells treated with macrophage CM for 48 h. n = 3.

**P < 0.01, ***P < 0.001, ****P < 0.0001.


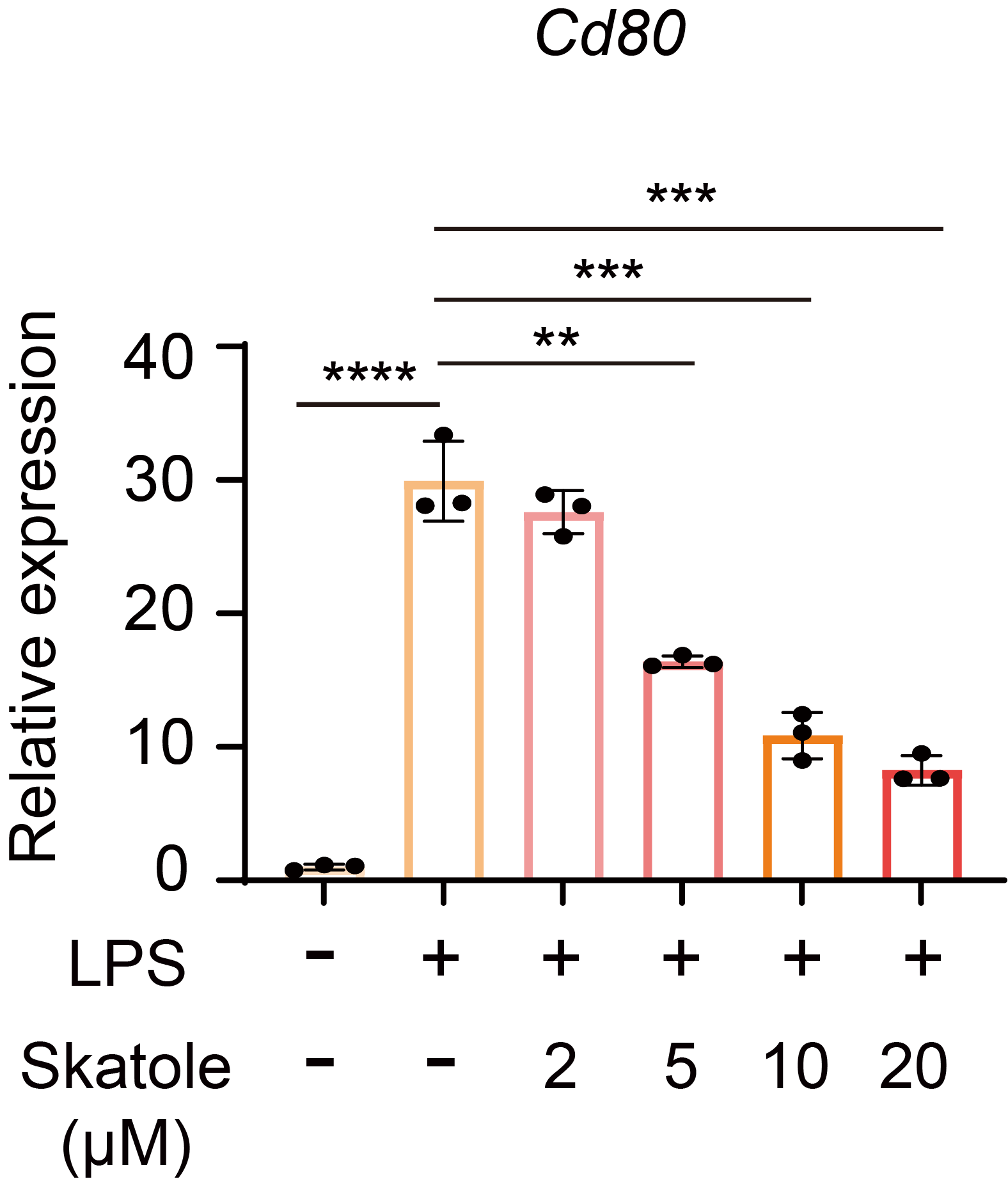


Fig. S7. *Cd80* is highly expressed in M1 macrophages and is inhibited by Skatole.


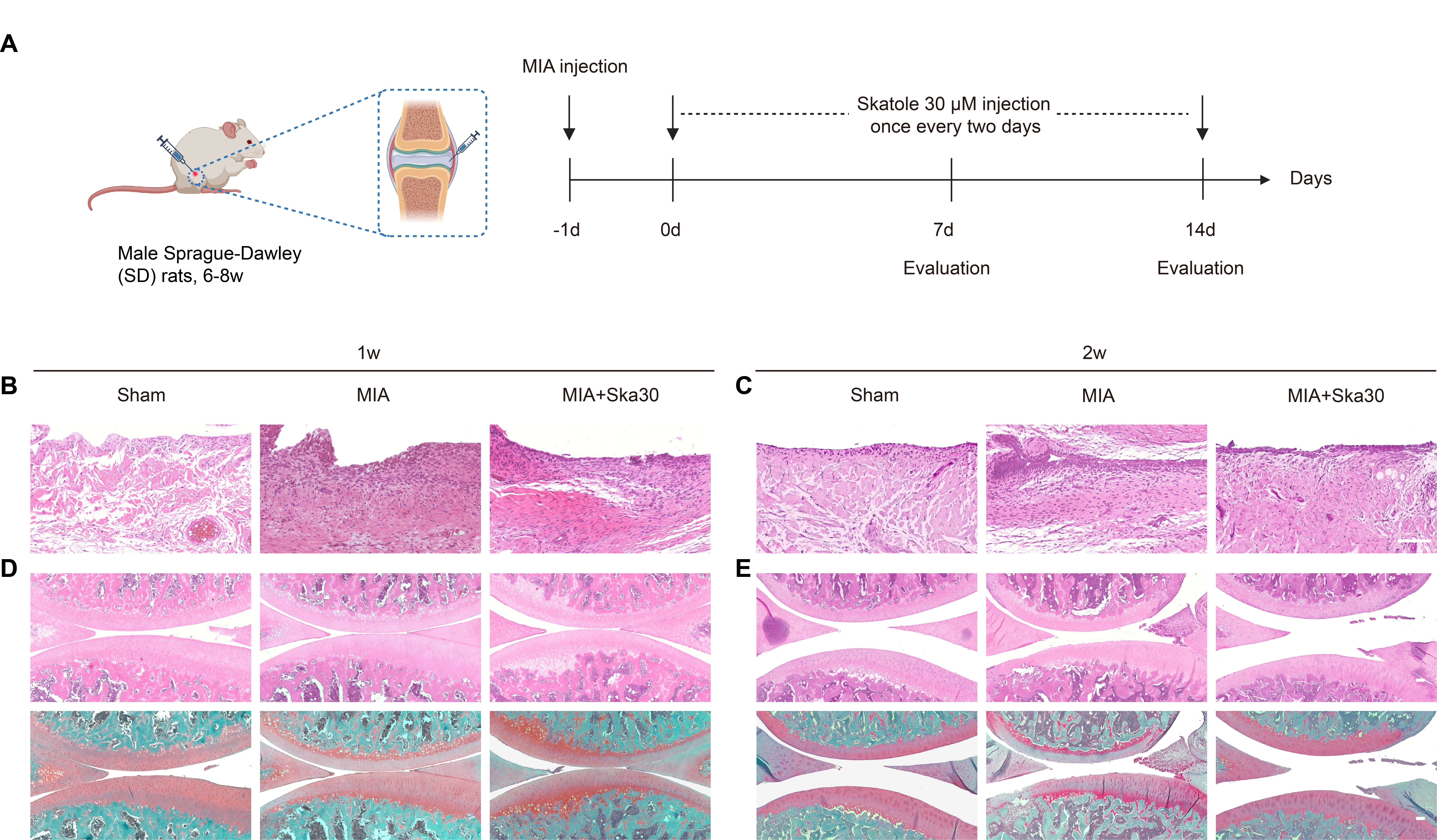


Fig. S8. Skatole inhibits MIA-induced synovial inflammation and further alleviates cartilage damage. (A) Schematic diagram of the experimental design for intra-articular injection of Skatole in a MIA-induced OA rat model. H&E staining of synovial tissue at 1 week (B) and 2 weeks (C). Scale bar, 100 μm. H&E staining and S&F staining of the joints at 1 week (D) and 2 weeks (E) after treatment.


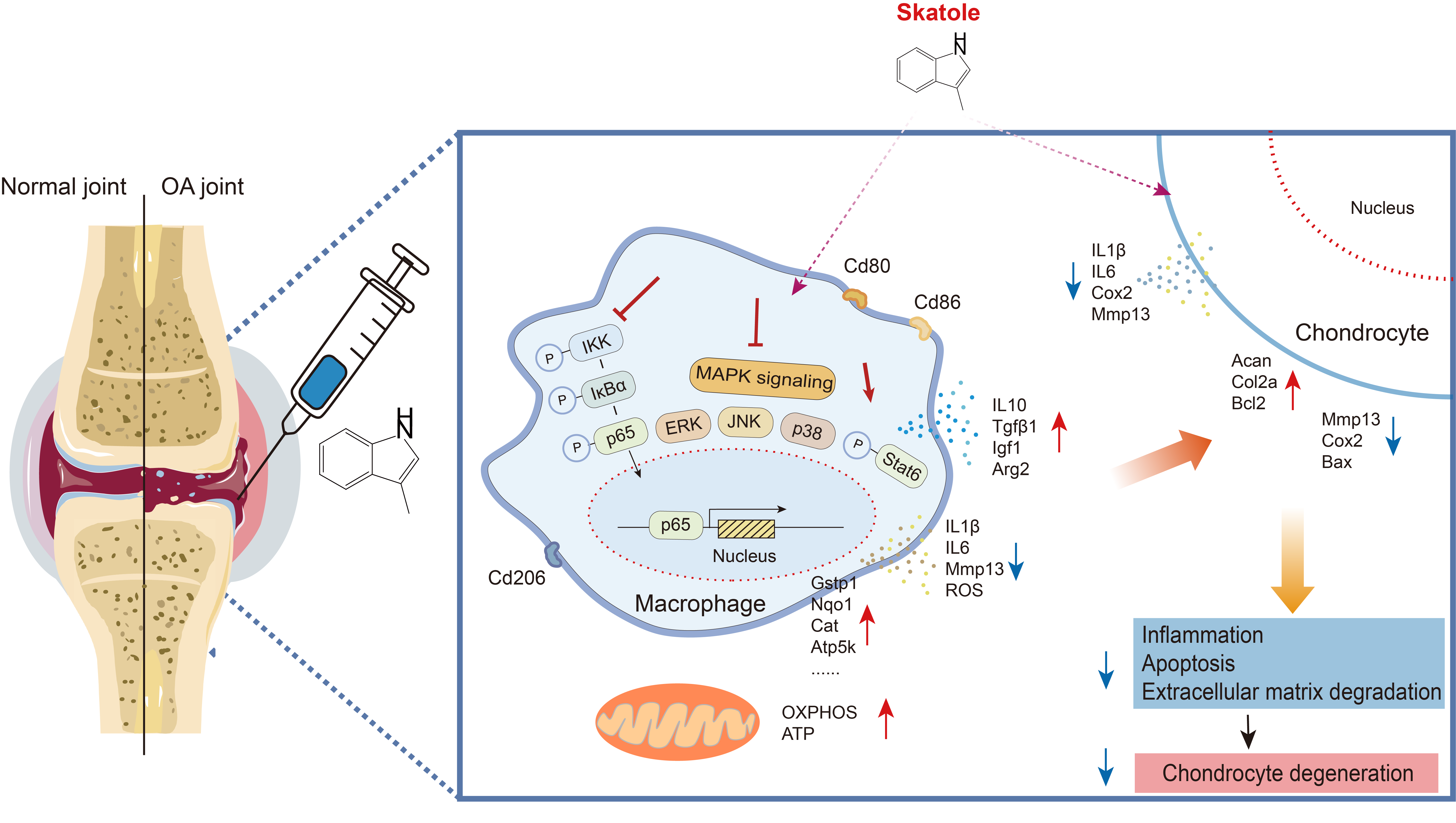
 Fig. S9. Pattern diagram of Skatole treating osteoarthritis by regulating macrophage polarization, maintaining joint immune microenvironment homeostasis, and protecting chondrocytes.


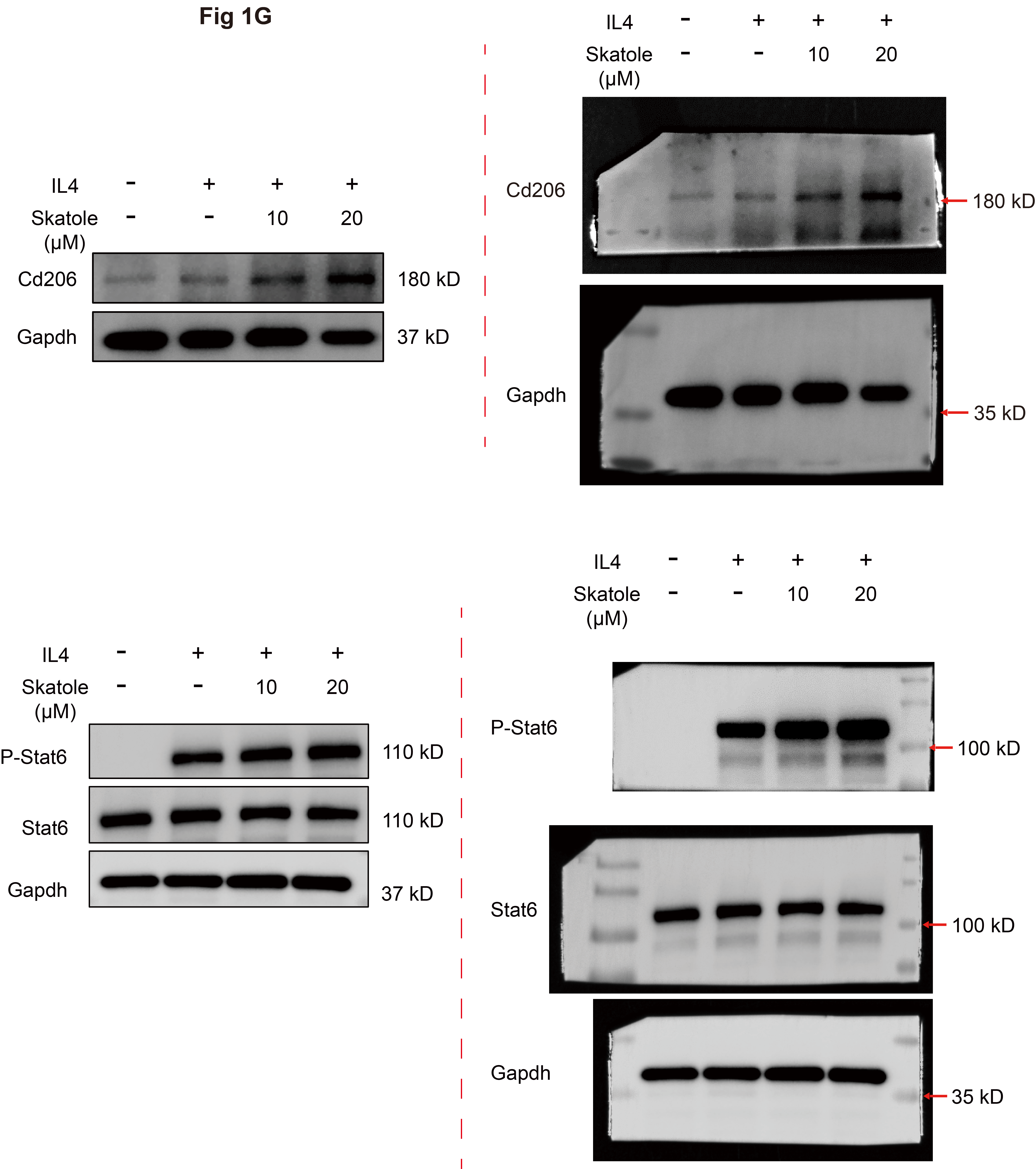


Fig. S10. Original full length western blots corresponding to Figure 1G.


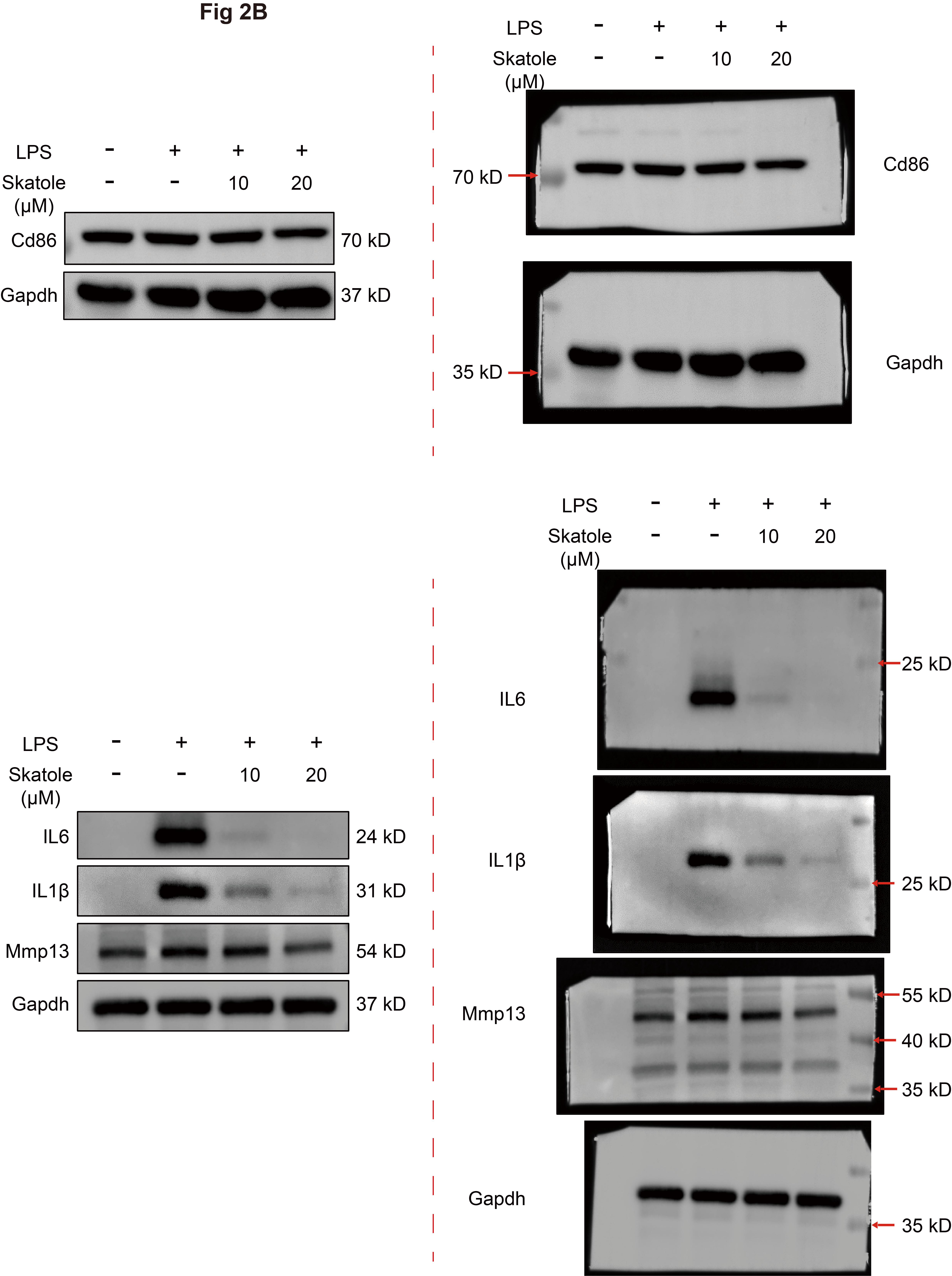


Fig. S11. Original full length western blots corresponding to Figure 2B.



 Fig. S12. Original full length western blots corresponding to Figure 3A and Figure 3E.



 Fig. S13. Original full length western blots corresponding to Figure 6F and Figure 6J.

Ethical approval statement


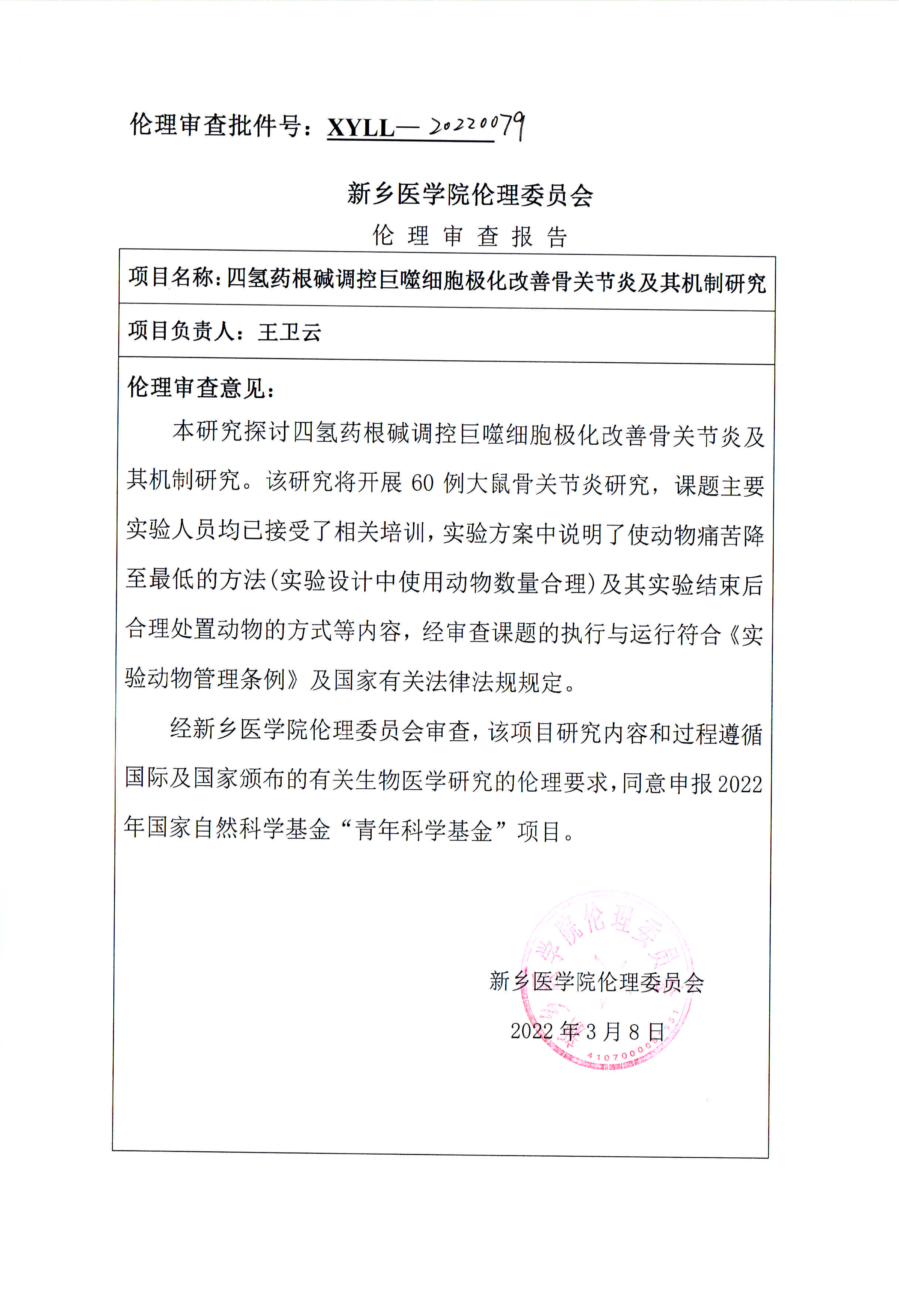


**Table S1:** Reagent or resource

| **Reagent or resource** | **Source** | **Identifier** |
| --- | --- | --- |
| **Antibodies** | | |
| Anti-Cd206 (IF) | Abcam | Cat#ab64693 |
| Anti-Cd206 (Co-IF, FACS) | Proteintech | Cat#60143-1-Ig |
| Anti-Stat6 | Cell Signaling Technology | Cat#5397 |
| Anti-P-Stat6 | Cell Signaling Technology | Cat#56554 |
| Anti-Cd86 | HUABIO | Cat#ET1606 |
| Anti-Gapdh | Affinity | Cat#AF7021 |
| Anti-Il6 | Cell Signaling Technology | Cat#12912 |
| Anti-Il1β | Cell Signaling Technology | Cat#12426 |
| Anti-Mmp13 | Proteintech | Cat#18165-1-AP |
| Anti-P-IKK | Cell Signaling Technology | Cat#2697 |
| Anti-IKKβ | Cell Signaling Technology | Cat#8943 |
| Anti-P-IκBα | Cell Signaling Technology | Cat#2859 |
| Anti-IκBα | Cell Signaling Technology | Cat#4814 |
| Anti-P-p65 (WB, CUT&Tag) | Cell Signaling Technology | Cat#3033 |
| Anti-P-p65 (IF) | Zenbio | Cat#310013 |
| Anti-p65 | Cell Signaling Technology | Cat#8242 |
| Anti-P-ERK | Cell Signaling Technology | Cat#4370 |
| Anti-ERK | Cell Signaling Technology | Cat#4695 |
| Anti-JNK | Cell Signaling Technology | Cat#9252 |
| Anti-P-JNK | Cell Signaling Technology | Cat#[4668](https://www.cellsignal.com/products/primary-antibodies/phospho-sapk-jnk-thr183-tyr185-81e11-rabbit-mab/4668) |
| Anti-P-p38 | Cell Signaling Technology | Cat#4511 |
| Anti-p38 | Cell Signaling Technology | Cat#8690 |
| Anti-Bax | ABclonal | Cat#A15633 |
| Anti-Bcl2 | ABclonal | Cat#A0208 |
| Anti-Cox2 | Proteintech | Cat#66351-1-Ig |
| Anti-Acan | Abcam | Cat#ab313636 |
| Anti-Collagen II | Arigo | Cat#ARG20787 |
| Anti-Cd80 | Proteintech | Cat#14292-1-AP |
| Goat Anti-Rabbit IgG H&L (Alexa Fluor® 555) | Abcam | Cat#ab150078 |
| Cy3–conjugated Affinipure Donkey Anti-Goat IgG(H+L) | Proteintech | Cat# SA00009-3 |
| HRP, Goat Anti-Mouse IgG | Abbkine | Cat#A21010 |
| HRP, Goat Anti-Rabbit IgG | Abbkine | Cat#A21020 |
| Rabbit Anti-Goat IgG H&L (HRP) | ZSGB-BIO | Cat#ZB-2306 |
| CoraLite488-conjugated Goat Anti-Mouse IgG(H+L) | Proteintech | Cat#SA00013-1 |
| Goat anti-Rabbit IgG | Proteintech | Cat#B900210 |
| **Chemicals, peptides, and recombinant proteins** | | |
| Skatole | MCE | Cat#HY-W007355 |
| Kinsenosid | TargetMol | T3849 |
| Carveol | TargetMol | TN3604 |
| Fargesin | TargetMol | T5S2178 |
| DMSO | Sigma-[Aldrich](https://www.baidu.com/other.php?url=0f00000gBrgDLroP9ssR6BFfg_NWIV1f7qhDBbjGX5111hM2pULd-eU8czEgFV27yceSIIaDaZy2gB48vEbeyAN2AcCwyR1D2ACX7oO02N3Mm7ckr_rj_w26mhEJ4Z9td18a4UxBYlMI06IbJ5omLr7zRH-fUpHloAFImN2jNNcPXJD2IAiKkesCz4mkCWckk2IZfoXtidJY0gkUgYJm_OgwR_rh.7Y_NR2Ar5Od663rj6tCLlVQQPAlutPgFKt54mrAJa7MHWuxJBW6YECYgsGCYTluCtOZ5uM8xJxfuC4OCIwCrJYlRYD1It7jHzs_lTUQqRHZon17I9zyxzZwFs4QnSZFutPH3hX8a9G4I2UM3PQDUqy-Mr1w8hxjNzL1nkgTQCbqT7jHzk8sHfGmEukmr8a9G4mgwRDkRA2b_LRgkFl32AM-CFhY_pgYpQ3RYprInYXxWM_1L5d5CnYTOYrCYZNpI2pau-2s1f_TTh1Wm.TLFWgv-b5HDkrfK1ThPGujYknHb0THY0IAYqTv-MUyD0IgP-T-qYXgK-5H00mywxIZ-suHYv0ZIEThfqTv-MUyD0ThPv5H00IgF_gv-b5HDdPWfsrjcLnWc0UgNxpyfqnHm1PHmznjD0UNqGujYkn1mvrHD1PsKVIZK_gv-b5HDzrjcv0ZKvgv-b5H00pywW5R9awfKspyfqnHf0mv-b5HnkPsKWThnqPWnLrjf&ck=3033.13.1725807142073.0.0.516.151.0&dt=1725807133&wd=Sigma&tpl=tpl_12826_36977_0&l=1564082722&ai=0_55695685_1_0&us=linkVersion%3D1%26compPath%3D10036.0-10032.0%26label%3D%25E4%25B8%25BB%25E6%25A0%2587%25E9%25A2%2598%26linkType%3D%26linkText%3DMillicell%2520ERS%25203.0%2520TE) | Cat#D2650-100ML |
| LPS | Sigma-[Aldrich](https://www.baidu.com/other.php?url=0f00000gBrgDLroP9ssR6BFfg_NWIV1f7qhDBbjGX5111hM2pULd-eU8czEgFV27yceSIIaDaZy2gB48vEbeyAN2AcCwyR1D2ACX7oO02N3Mm7ckr_rj_w26mhEJ4Z9td18a4UxBYlMI06IbJ5omLr7zRH-fUpHloAFImN2jNNcPXJD2IAiKkesCz4mkCWckk2IZfoXtidJY0gkUgYJm_OgwR_rh.7Y_NR2Ar5Od663rj6tCLlVQQPAlutPgFKt54mrAJa7MHWuxJBW6YECYgsGCYTluCtOZ5uM8xJxfuC4OCIwCrJYlRYD1It7jHzs_lTUQqRHZon17I9zyxzZwFs4QnSZFutPH3hX8a9G4I2UM3PQDUqy-Mr1w8hxjNzL1nkgTQCbqT7jHzk8sHfGmEukmr8a9G4mgwRDkRA2b_LRgkFl32AM-CFhY_pgYpQ3RYprInYXxWM_1L5d5CnYTOYrCYZNpI2pau-2s1f_TTh1Wm.TLFWgv-b5HDkrfK1ThPGujYknHb0THY0IAYqTv-MUyD0IgP-T-qYXgK-5H00mywxIZ-suHYv0ZIEThfqTv-MUyD0ThPv5H00IgF_gv-b5HDdPWfsrjcLnWc0UgNxpyfqnHm1PHmznjD0UNqGujYkn1mvrHD1PsKVIZK_gv-b5HDzrjcv0ZKvgv-b5H00pywW5R9awfKspyfqnHf0mv-b5HnkPsKWThnqPWnLrjf&ck=3033.13.1725807142073.0.0.516.151.0&dt=1725807133&wd=Sigma&tpl=tpl_12826_36977_0&l=1564082722&ai=0_55695685_1_0&us=linkVersion%3D1%26compPath%3D10036.0-10032.0%26label%3D%25E4%25B8%25BB%25E6%25A0%2587%25E9%25A2%2598%26linkType%3D%26linkText%3DMillicell%2520ERS%25203.0%2520TE) | Cat#L4516-1MG |
| Recombinant Murine IL-4 | Peprotech | Cat#214-14-20UG |
| Recombinant Murine IL-1β | Peprotech | Cat#211-11B-10UG |
| Recombinant Mouse lFN gamma Protein | Sino Biological | Cat#50709-MNAH |
| Recombinant Mouse M-CSF/CSF1 Protein | Sino Biological | Cat#51112-MNAH |
| DMEM-high | WISENT | Cat#319-005-CL |
| DMEM-low | WISENT | Cat#319-010-CL |
| DMEM/F12 | WISENT | Cat# 319-075-CL |
| RPMI Medium 1640 basic | Gibco | Cat#C11875500BT |
| XF DMEM Medium, pH 7.4 | Agilent Technologies | Cat#103575-100 |
| L-glutamine | Sigma-[Aldrich](https://www.baidu.com/other.php?url=0f00000gBrgDLroP9ssR6BFfg_NWIV1f7qhDBbjGX5111hM2pULd-eU8czEgFV27yceSIIaDaZy2gB48vEbeyAN2AcCwyR1D2ACX7oO02N3Mm7ckr_rj_w26mhEJ4Z9td18a4UxBYlMI06IbJ5omLr7zRH-fUpHloAFImN2jNNcPXJD2IAiKkesCz4mkCWckk2IZfoXtidJY0gkUgYJm_OgwR_rh.7Y_NR2Ar5Od663rj6tCLlVQQPAlutPgFKt54mrAJa7MHWuxJBW6YECYgsGCYTluCtOZ5uM8xJxfuC4OCIwCrJYlRYD1It7jHzs_lTUQqRHZon17I9zyxzZwFs4QnSZFutPH3hX8a9G4I2UM3PQDUqy-Mr1w8hxjNzL1nkgTQCbqT7jHzk8sHfGmEukmr8a9G4mgwRDkRA2b_LRgkFl32AM-CFhY_pgYpQ3RYprInYXxWM_1L5d5CnYTOYrCYZNpI2pau-2s1f_TTh1Wm.TLFWgv-b5HDkrfK1ThPGujYknHb0THY0IAYqTv-MUyD0IgP-T-qYXgK-5H00mywxIZ-suHYv0ZIEThfqTv-MUyD0ThPv5H00IgF_gv-b5HDdPWfsrjcLnWc0UgNxpyfqnHm1PHmznjD0UNqGujYkn1mvrHD1PsKVIZK_gv-b5HDzrjcv0ZKvgv-b5H00pywW5R9awfKspyfqnHf0mv-b5HnkPsKWThnqPWnLrjf&ck=3033.13.1725807142073.0.0.516.151.0&dt=1725807133&wd=Sigma&tpl=tpl_12826_36977_0&l=1564082722&ai=0_55695685_1_0&us=linkVersion%3D1%26compPath%3D10036.0-10032.0%26label%3D%25E4%25B8%25BB%25E6%25A0%2587%25E9%25A2%2598%26linkType%3D%26linkText%3DMillicell%2520ERS%25203.0%2520TE) | Cat#G7513 |
| Sodium pyruvate | Sigma-[Aldrich](https://www.baidu.com/other.php?url=0f00000gBrgDLroP9ssR6BFfg_NWIV1f7qhDBbjGX5111hM2pULd-eU8czEgFV27yceSIIaDaZy2gB48vEbeyAN2AcCwyR1D2ACX7oO02N3Mm7ckr_rj_w26mhEJ4Z9td18a4UxBYlMI06IbJ5omLr7zRH-fUpHloAFImN2jNNcPXJD2IAiKkesCz4mkCWckk2IZfoXtidJY0gkUgYJm_OgwR_rh.7Y_NR2Ar5Od663rj6tCLlVQQPAlutPgFKt54mrAJa7MHWuxJBW6YECYgsGCYTluCtOZ5uM8xJxfuC4OCIwCrJYlRYD1It7jHzs_lTUQqRHZon17I9zyxzZwFs4QnSZFutPH3hX8a9G4I2UM3PQDUqy-Mr1w8hxjNzL1nkgTQCbqT7jHzk8sHfGmEukmr8a9G4mgwRDkRA2b_LRgkFl32AM-CFhY_pgYpQ3RYprInYXxWM_1L5d5CnYTOYrCYZNpI2pau-2s1f_TTh1Wm.TLFWgv-b5HDkrfK1ThPGujYknHb0THY0IAYqTv-MUyD0IgP-T-qYXgK-5H00mywxIZ-suHYv0ZIEThfqTv-MUyD0ThPv5H00IgF_gv-b5HDdPWfsrjcLnWc0UgNxpyfqnHm1PHmznjD0UNqGujYkn1mvrHD1PsKVIZK_gv-b5HDzrjcv0ZKvgv-b5H00pywW5R9awfKspyfqnHf0mv-b5HnkPsKWThnqPWnLrjf&ck=3033.13.1725807142073.0.0.516.151.0&dt=1725807133&wd=Sigma&tpl=tpl_12826_36977_0&l=1564082722&ai=0_55695685_1_0&us=linkVersion%3D1%26compPath%3D10036.0-10032.0%26label%3D%25E4%25B8%25BB%25E6%25A0%2587%25E9%25A2%2598%26linkType%3D%26linkText%3DMillicell%2520ERS%25203.0%2520TE) | Cat#S8636 |
| Glucose | Sigma-[Aldrich](https://www.baidu.com/other.php?url=0f00000gBrgDLroP9ssR6BFfg_NWIV1f7qhDBbjGX5111hM2pULd-eU8czEgFV27yceSIIaDaZy2gB48vEbeyAN2AcCwyR1D2ACX7oO02N3Mm7ckr_rj_w26mhEJ4Z9td18a4UxBYlMI06IbJ5omLr7zRH-fUpHloAFImN2jNNcPXJD2IAiKkesCz4mkCWckk2IZfoXtidJY0gkUgYJm_OgwR_rh.7Y_NR2Ar5Od663rj6tCLlVQQPAlutPgFKt54mrAJa7MHWuxJBW6YECYgsGCYTluCtOZ5uM8xJxfuC4OCIwCrJYlRYD1It7jHzs_lTUQqRHZon17I9zyxzZwFs4QnSZFutPH3hX8a9G4I2UM3PQDUqy-Mr1w8hxjNzL1nkgTQCbqT7jHzk8sHfGmEukmr8a9G4mgwRDkRA2b_LRgkFl32AM-CFhY_pgYpQ3RYprInYXxWM_1L5d5CnYTOYrCYZNpI2pau-2s1f_TTh1Wm.TLFWgv-b5HDkrfK1ThPGujYknHb0THY0IAYqTv-MUyD0IgP-T-qYXgK-5H00mywxIZ-suHYv0ZIEThfqTv-MUyD0ThPv5H00IgF_gv-b5HDdPWfsrjcLnWc0UgNxpyfqnHm1PHmznjD0UNqGujYkn1mvrHD1PsKVIZK_gv-b5HDzrjcv0ZKvgv-b5H00pywW5R9awfKspyfqnHf0mv-b5HnkPsKWThnqPWnLrjf&ck=3033.13.1725807142073.0.0.516.151.0&dt=1725807133&wd=Sigma&tpl=tpl_12826_36977_0&l=1564082722&ai=0_55695685_1_0&us=linkVersion%3D1%26compPath%3D10036.0-10032.0%26label%3D%25E4%25B8%25BB%25E6%25A0%2587%25E9%25A2%2598%26linkType%3D%26linkText%3DMillicell%2520ERS%25203.0%2520TE) | Cat#G8769 |
| Fetal bovine serum | WISENT | Cat#086-150 |
| Penicillin - Streptomycin Solution | Cytiva | Cat#SV30010 |
| monosodium iodoacetate (MIA) | Sigma-[Aldrich](https://www.baidu.com/other.php?url=0f00000gBrgDLroP9ssR6BFfg_NWIV1f7qhDBbjGX5111hM2pULd-eU8czEgFV27yceSIIaDaZy2gB48vEbeyAN2AcCwyR1D2ACX7oO02N3Mm7ckr_rj_w26mhEJ4Z9td18a4UxBYlMI06IbJ5omLr7zRH-fUpHloAFImN2jNNcPXJD2IAiKkesCz4mkCWckk2IZfoXtidJY0gkUgYJm_OgwR_rh.7Y_NR2Ar5Od663rj6tCLlVQQPAlutPgFKt54mrAJa7MHWuxJBW6YECYgsGCYTluCtOZ5uM8xJxfuC4OCIwCrJYlRYD1It7jHzs_lTUQqRHZon17I9zyxzZwFs4QnSZFutPH3hX8a9G4I2UM3PQDUqy-Mr1w8hxjNzL1nkgTQCbqT7jHzk8sHfGmEukmr8a9G4mgwRDkRA2b_LRgkFl32AM-CFhY_pgYpQ3RYprInYXxWM_1L5d5CnYTOYrCYZNpI2pau-2s1f_TTh1Wm.TLFWgv-b5HDkrfK1ThPGujYknHb0THY0IAYqTv-MUyD0IgP-T-qYXgK-5H00mywxIZ-suHYv0ZIEThfqTv-MUyD0ThPv5H00IgF_gv-b5HDdPWfsrjcLnWc0UgNxpyfqnHm1PHmznjD0UNqGujYkn1mvrHD1PsKVIZK_gv-b5HDzrjcv0ZKvgv-b5H00pywW5R9awfKspyfqnHf0mv-b5HnkPsKWThnqPWnLrjf&ck=3033.13.1725807142073.0.0.516.151.0&dt=1725807133&wd=Sigma&tpl=tpl_12826_36977_0&l=1564082722&ai=0_55695685_1_0&us=linkVersion%3D1%26compPath%3D10036.0-10032.0%26label%3D%25E4%25B8%25BB%25E6%25A0%2587%25E9%25A2%2598%26linkType%3D%26linkText%3DMillicell%2520ERS%25203.0%2520TE) | Cat#57858 |
| DAPI Staining Solution | Beyotime | Cat#C1005 |
| Antifade Mounting Medium with DAPI | Beyotime | Cat#P0131 |
| Erythrocytes lysate | Biosharp | Cat#BL503A |
| Collagenase from Clostridium histolyticum | Sigma-[Aldrich](https://www.baidu.com/other.php?url=0f00000gBrgDLroP9ssR6BFfg_NWIV1f7qhDBbjGX5111hM2pULd-eU8czEgFV27yceSIIaDaZy2gB48vEbeyAN2AcCwyR1D2ACX7oO02N3Mm7ckr_rj_w26mhEJ4Z9td18a4UxBYlMI06IbJ5omLr7zRH-fUpHloAFImN2jNNcPXJD2IAiKkesCz4mkCWckk2IZfoXtidJY0gkUgYJm_OgwR_rh.7Y_NR2Ar5Od663rj6tCLlVQQPAlutPgFKt54mrAJa7MHWuxJBW6YECYgsGCYTluCtOZ5uM8xJxfuC4OCIwCrJYlRYD1It7jHzs_lTUQqRHZon17I9zyxzZwFs4QnSZFutPH3hX8a9G4I2UM3PQDUqy-Mr1w8hxjNzL1nkgTQCbqT7jHzk8sHfGmEukmr8a9G4mgwRDkRA2b_LRgkFl32AM-CFhY_pgYpQ3RYprInYXxWM_1L5d5CnYTOYrCYZNpI2pau-2s1f_TTh1Wm.TLFWgv-b5HDkrfK1ThPGujYknHb0THY0IAYqTv-MUyD0IgP-T-qYXgK-5H00mywxIZ-suHYv0ZIEThfqTv-MUyD0ThPv5H00IgF_gv-b5HDdPWfsrjcLnWc0UgNxpyfqnHm1PHmznjD0UNqGujYkn1mvrHD1PsKVIZK_gv-b5HDzrjcv0ZKvgv-b5H00pywW5R9awfKspyfqnHf0mv-b5HnkPsKWThnqPWnLrjf&ck=3033.13.1725807142073.0.0.516.151.0&dt=1725807133&wd=Sigma&tpl=tpl_12826_36977_0&l=1564082722&ai=0_55695685_1_0&us=linkVersion%3D1%26compPath%3D10036.0-10032.0%26label%3D%25E4%25B8%25BB%25E6%25A0%2587%25E9%25A2%2598%26linkType%3D%26linkText%3DMillicell%2520ERS%25203.0%2520TE) | Cat#C6885-500MG |
| DAB | ZSGB-BIO | Cat#ZLI-9018 |
| **Assay kits** | | |
| Reactive Oxygen Species Assay Kit | Beyotime | Cat#S0033 |
| Enhanced BCA Protein Assay Kit | Beyotime | Cat#P0010 |
| SDS-PAGE Gel Quick Preparation Kit | Beyotime | Cat#P0012AC |
| ECL Basic Plus Kit | ABclonal | Cat#RM00020P |
| RNA-Quick Purification Kit | ES Science | Cat#RN001 |
| Hiscript Ⅲ 1st Strand cDNA Synthesis Kit (+gDNA wiper) | Vazyme | Cat#R312-02 |
| Taq Pro Universal SYBR qPCR Master Mix | Vazyme | Cat#Q712-02 |
| Mouse IL-1β Immunoassay | R&D Systems | Cat#201-LB-010/CF |
| Mouse IL-6 ELISA Kit | Proteintech | Cat#KE10007 |
| Mouse MMP-13(Matrix Metalloproteinase 13) ELISA Kit | Elabscience | Cat#48366 |
| Modified Saffron-O and Fast Green Stain Kit（For Bone） | Solarbio | Cat#G1371 |
| Cole's Hematoxylin Solution (For Conventional Stain) | Solarbio | Cat#G1140 |
| Eosin Y Stain Solution, For HE | Solarbio | Cat#G1100 |
| Alcian Blue Stain Kit | Solarbio | Cat#G1560 |
| [CellTiter 96® AQueous One Solution Cell Proliferation Assay](https://www.promega.com.cn/products/cell-health-assays/cell-viability-and-cytotoxicity-assays/celltiter-96-aqueous-one-solution-cell-proliferation-assay-_mts_/?catNum=G3580) | Promega | Cat#G3580 |
| In Situ Cell Death Detection Kit, TMR red | Roche | Cat#12156792910 |
| XF Cell Mito Stress Test Kit | Agilent Technologies | Cat#103015-100 |
| Enhanced ATP Assay Kit | Beyotime | Cat#S0027 |
| Enhanced NAD+/NADH Assay Kit with WST-8 | Beyotime | Cat#S0176S |
| NovoNGS® CUT&Tag® 4.0 High-Sensitivity Kit (for Illumina®) | Novoprotein | CatN259-YH01 |
| **Deposited data** | | |
| RNA-seq data | This paper | GEO: GSE261400 |
| CUT&Tag seq data | This paper | GEO: GSE283748 |
| **Experimental models: Cell & mouse lines** | | |
| RAW264.7 cell line | Procell | CL-0190 |
| Mouse: C57BL/6J | SiPeiFu | N/A |
| **Oligonucleotides** | | |
| Primers for RT–qPCR, see Tables S2 | This paper | N/A |
| **Software and algorithms** | | |
| GraphPad Prism | GraphPad Software | http://www.graphpad.com |
| ImageJ | ImageJ Software | [https://imagej.net/](https://link.zhihu.com/?target=https://imagej.net/Welcome) |

**Table S2:** List of primers used for RT-qPCR

| *Gapdh*-F | CCTTCATTGACCTCAACTACA |
| --- | --- |
| *Gapdh*-R | TAGACTCCACGACATACTCA |
| *Arg1*-F | CTGAGCTTTGATGTCGACGG |
| *Arg1*-R | TCCTCTGCTGTCTTCCCAAG |
| *Tnf*-F | TGAGGTCAATCTGCCCAAGT |
| *Tnf*-R | GGGGTCAGAGTAAAGGGGTC |
| *Mrc1*-F | CTCTGTTCAGCTATTGGACGC |
| *Mrc1*-R | CGGAATTTCTGGGATTCAGCTTC |
| *Il6*-F | CAACGATGATGCACTTGCAGA |
| *Il6*-R | TGTGACTCCAGCTTATCTCTTGG |
| *Il1b*-F | GCAACTGTTCCTGAACTCAACT |
| *Il1b*-R | ATCTTTTGGGGTCCGTCAACT |
| *Clec10a*-F | TGAGAAAGGCTTTAAGAACTGGG |
| *Clec10a*-R | GACCACCTGTAGTGATGTGGG |
| *Cd200r4*-F | TGCCACTGGGAGCAGAACAATG |
| *Cd200r4*-R | AGAGAATGGTCAGCAAGGAACGG |
| *Trem2*-F | CTGGAACCGTCACCATCACTC |
| *Trem2*-R | CGAAACTCGATGACTCCTCGG |
| *Il10*-F | ACCTGGTAGAAGTGATGCCC |
| *Il10*-R | AGGGTCTTCAGCTTCTCACC |
| *Tgfb1*-F | CTCCCGTGGCTTCTAGTGC |
| *Tgfb1*-R | GCCTTAGTTTGGACAGGATCTG |
| *Igf1*-F | CACATCATGTCGTCTTCACACC |
| *Igf1*-R | GGAAGCAACACTCATCCACAATG |
| *Arg2*-F | TCCTCCACGGGCAAATTCC |
| *Arg2*-R | GCTGGACCATATTCCACTCCTA |
| *Cd86*-F | TCAATGGGACTGCATATCTGCC |
| *Cd86*-R | GCCAAAATACTACCAGCTCACT |
| *Cd80*-F | ACCCCCAACATAACTGAGTCT |
| *Cd80*-R | TTCCAACCAAGAGAAGCGAGG |
| *Mmp13*-F | TTCTGGTCTTCTGGCACACG |
| *Mmp13*-R | TCGCCTGGACCATAAAGAAACT |
| *Mmp9*-F | CTGGACAGCCAGACACTAAAG |
| *Mmp9*-R | CTCGCGGCAAGTCTTCAGAG |
| *Ptgs2*-F | TGAGCAACTATTCCAAACCAGC |
| *Ptgs2*-R | GCACGTAGTCTTCGATCACTATC |
| *Acan*-F | AGGTGTCGCTCCCCAACTAT |
| *Acan*-R | CTTCACAGCGGTAGATCCCAG |
| *Col2a1*-F | GGGTCACAGAGGTTACCCAG |
| *Col2a1*-R | ACCAGGGGAACCACTCTCAC |
| *Sox9*-F | AGTACCCGCATCTGCACAAC |
| *Sox9*-R | ACGAAGGGTCTCTTCTCGCT |
