# Supplementary figures and images for: Skatole Alleviates Osteoarthritis by Reprogramming Macrophage Polarization and Protecting Chondrocytes
Source: Research (Wash D C). 2025 Feb 3;8:0604. doi: 10.34133/research.0604 (PMC11788598; doi:10.34133/research.0604)

**A**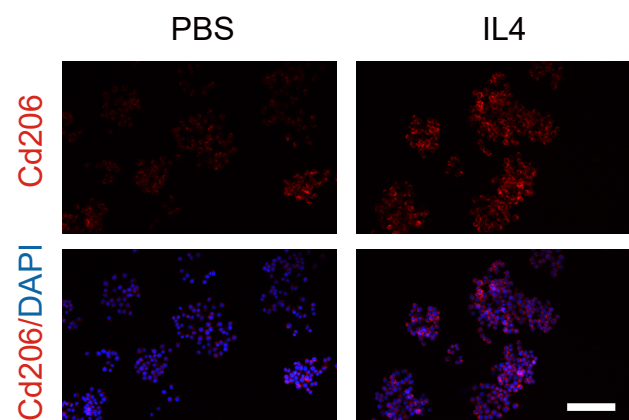**B**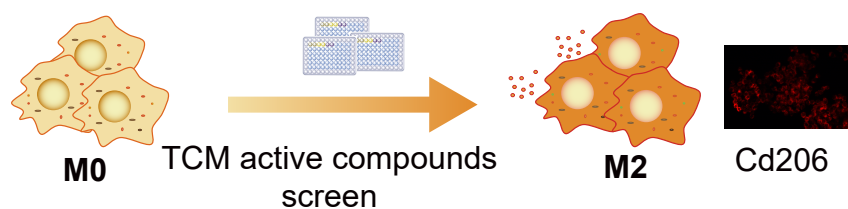**C**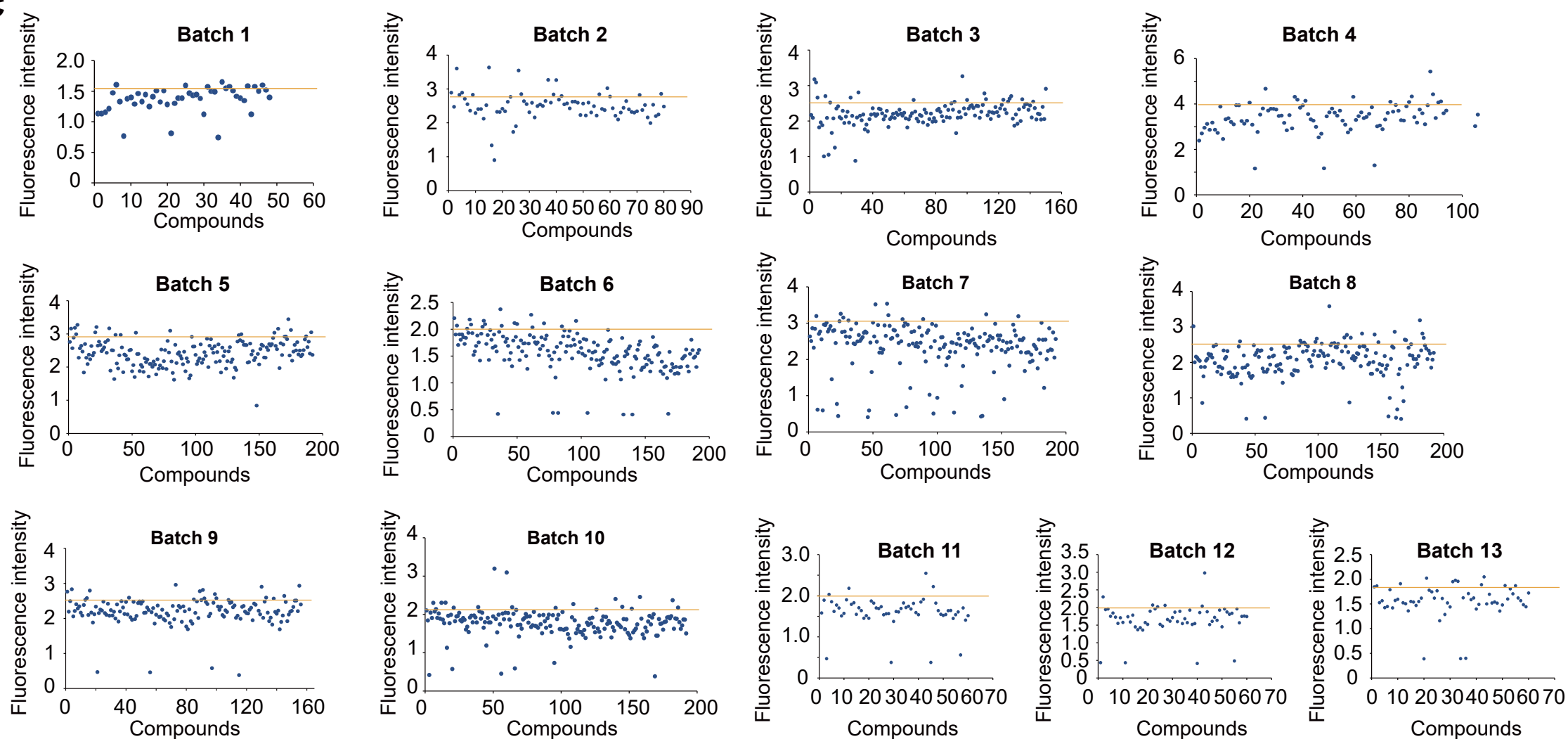**D**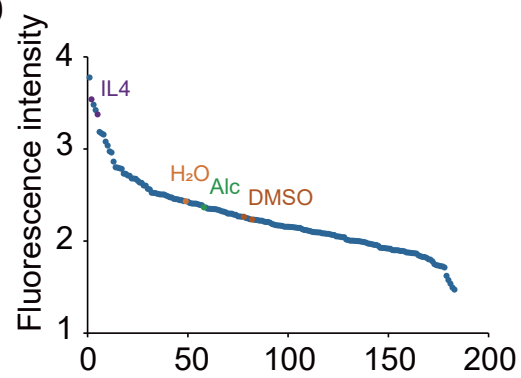**E**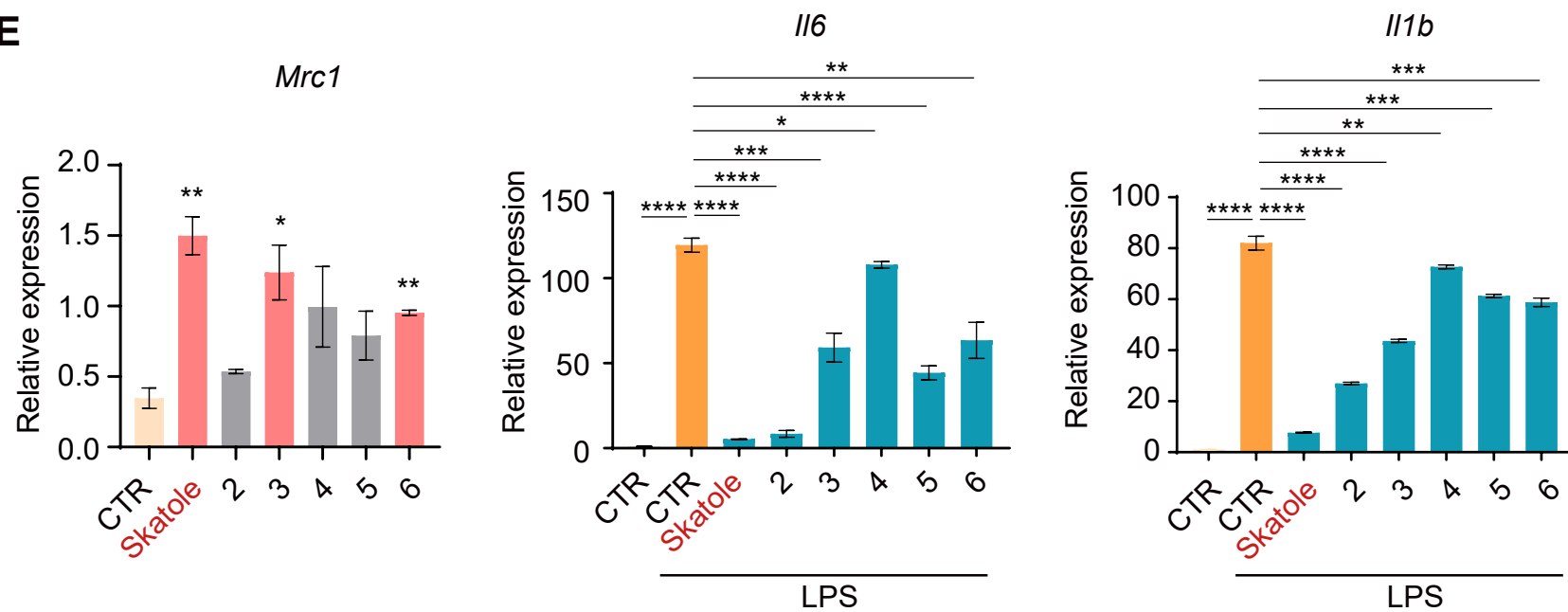

Supplement: Supplementary 1 — Figs. S1 to S13 Tables S1 and S2 [file research.0604.f1.zip › Fig S1.pdf]

**Fig 1G**

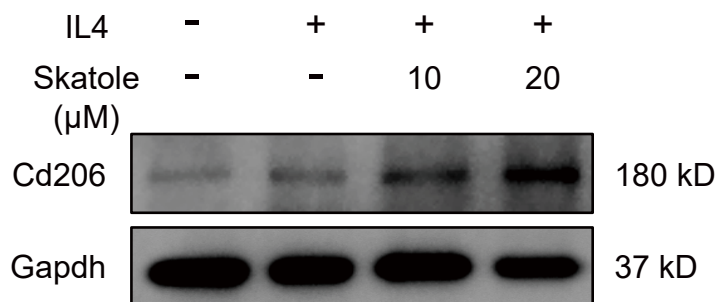

Cd206

Gapdh

| IL4          | - | + | +  | +  |
|--------------|---|---|----|----|
| Skatole (μM) | - | - | 10 | 20 |

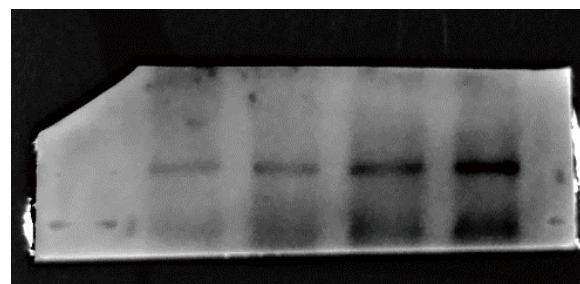

180 kD

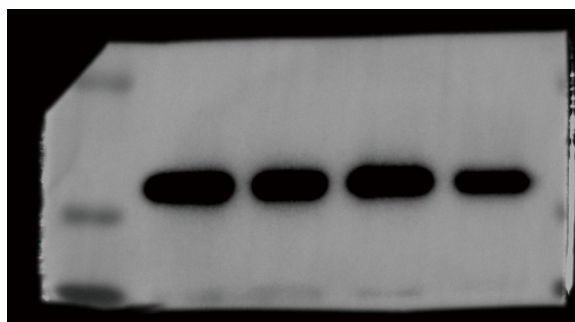

35 kD

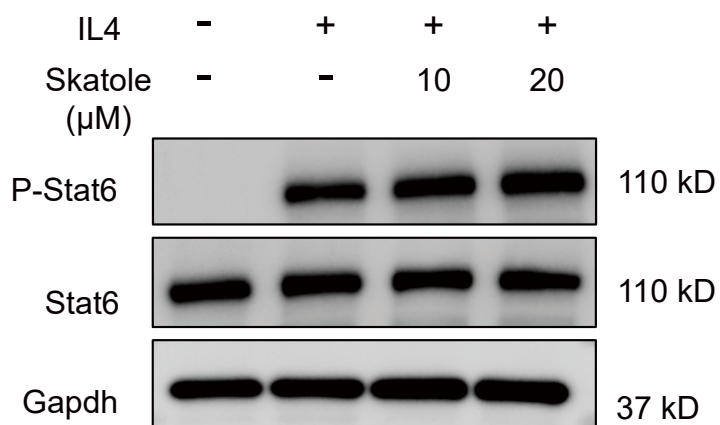

P-Stat6

Stat6

Gapdh

| IL4          | - | + | +  | +  |
|--------------|---|---|----|----|
| Skatole (μM) | - | - | 10 | 20 |

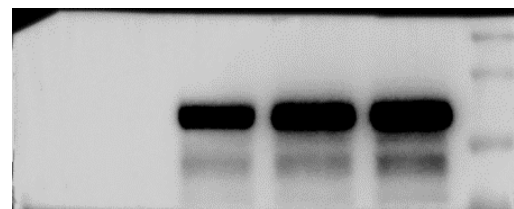

100 kD

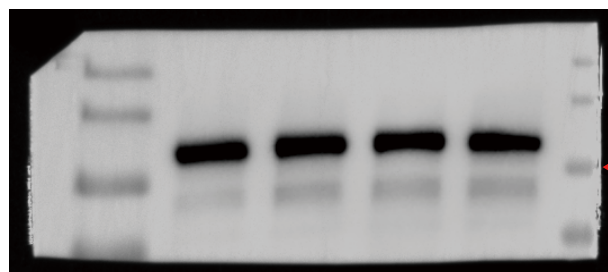

100 kD

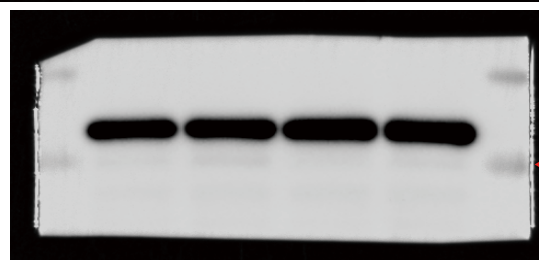

35 kD

Supplement: Supplementary 1 — Figs. S1 to S13 Tables S1 and S2 [file research.0604.f1.zip › Fig S10.pdf]

Fig 2B

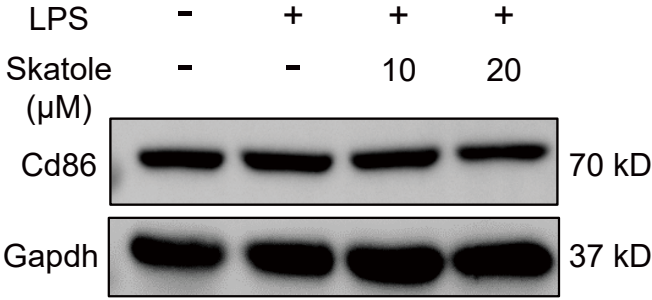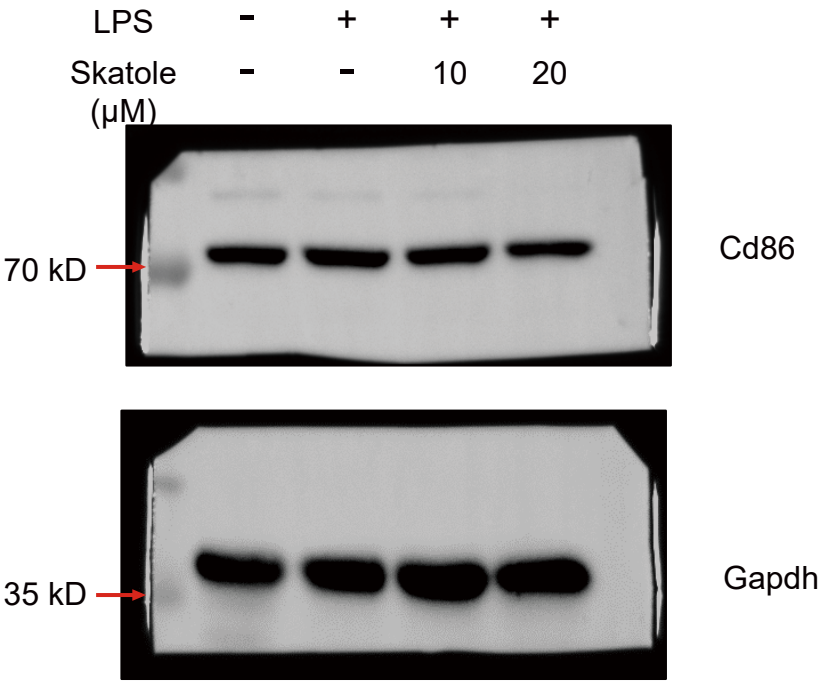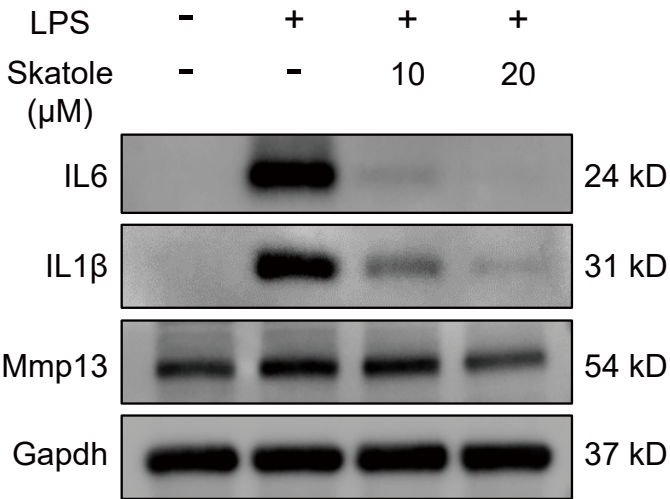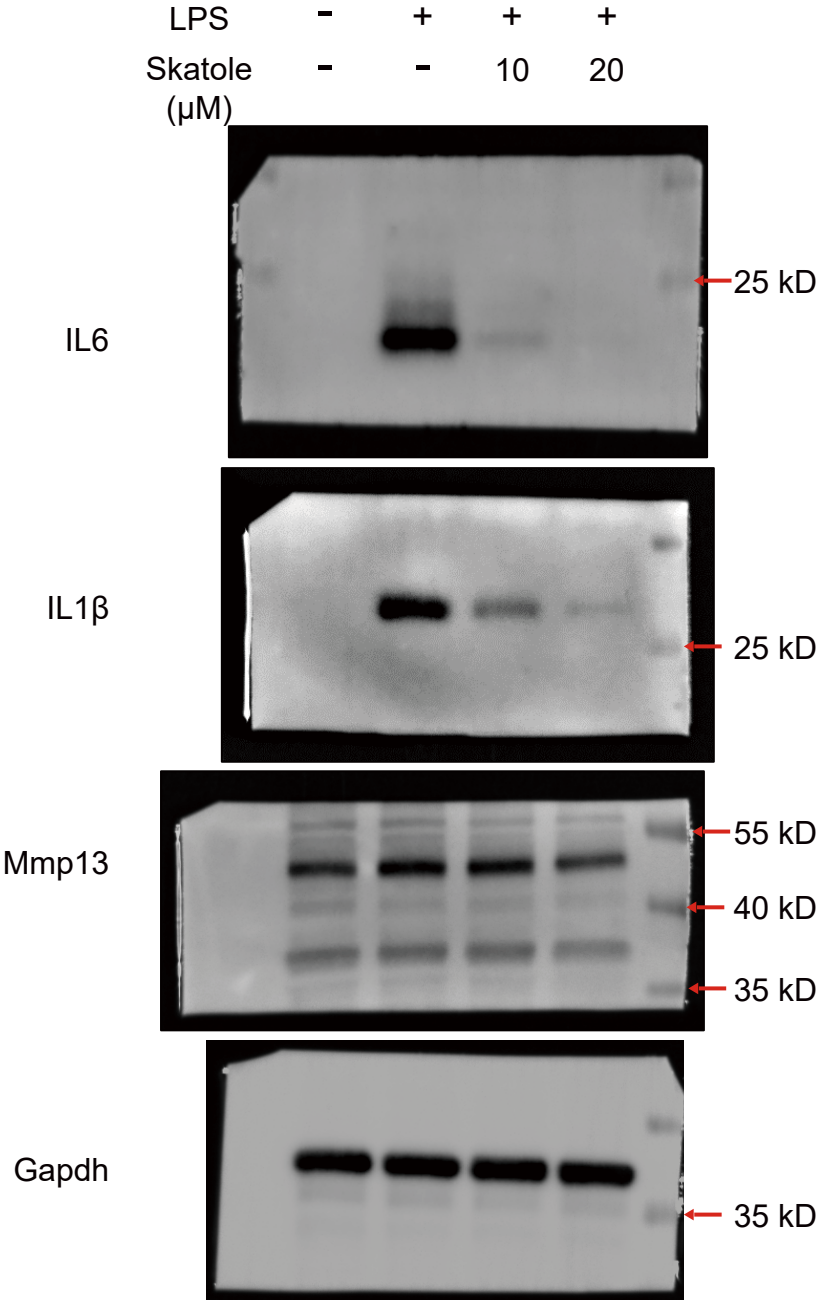

Supplement: Supplementary 1 — Figs. S1 to S13 Tables S1 and S2 [file research.0604.f1.zip › Fig S11.pdf]

Fig 3A

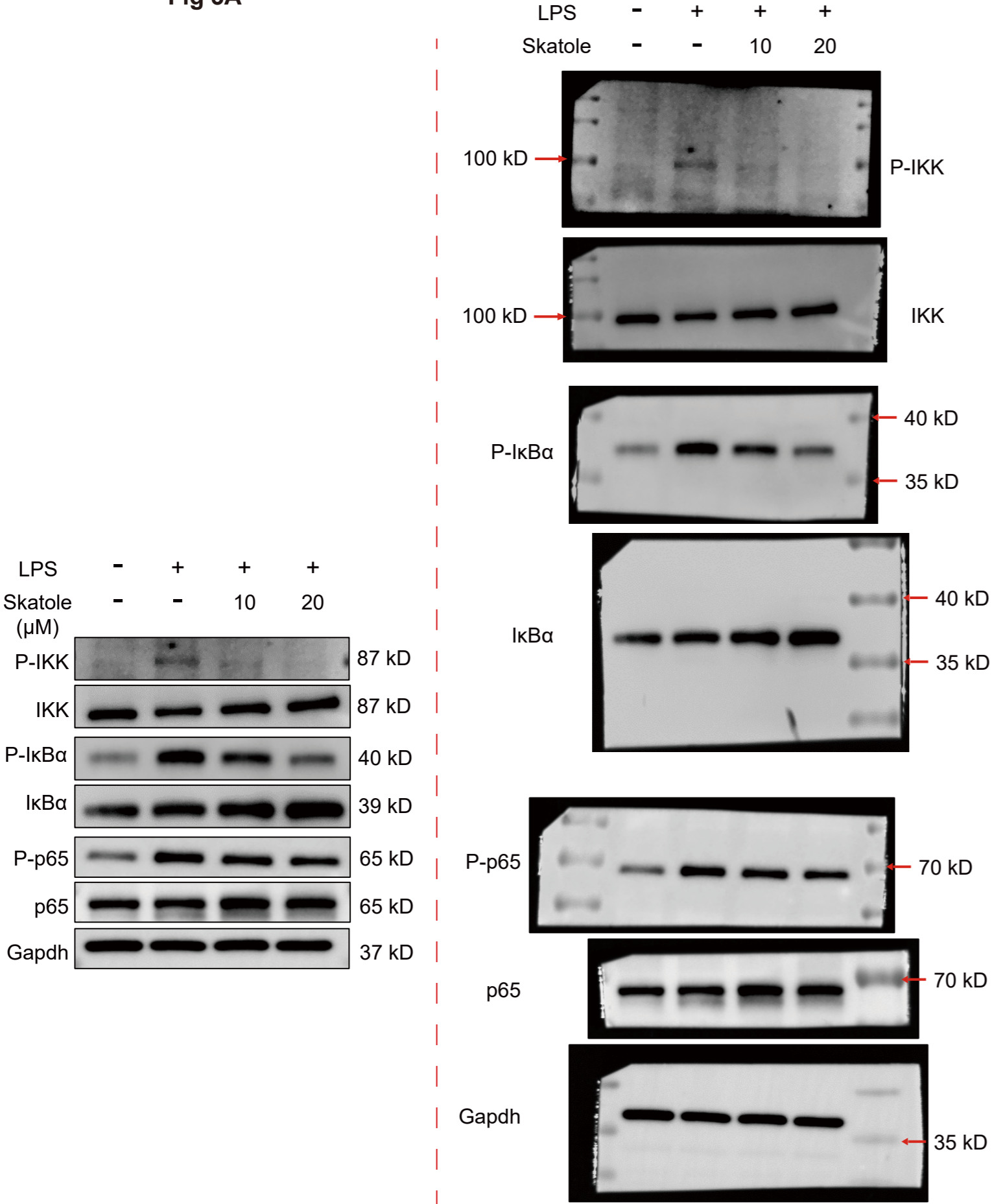

Fig 3E

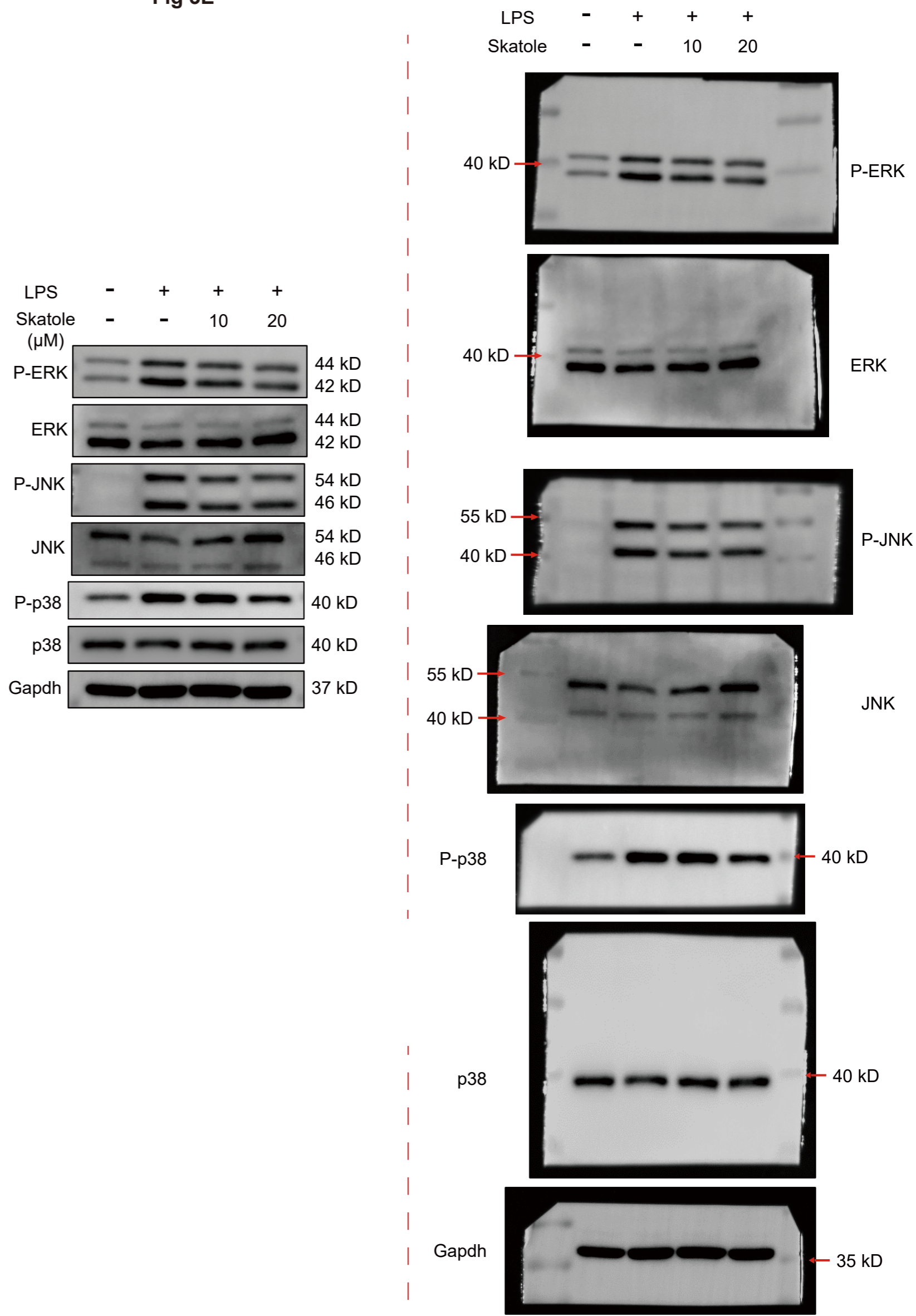

Supplement: Supplementary 1 — Figs. S1 to S13 Tables S1 and S2 [file research.0604.f1.zip › Fig S12.pdf]

Fig 6F

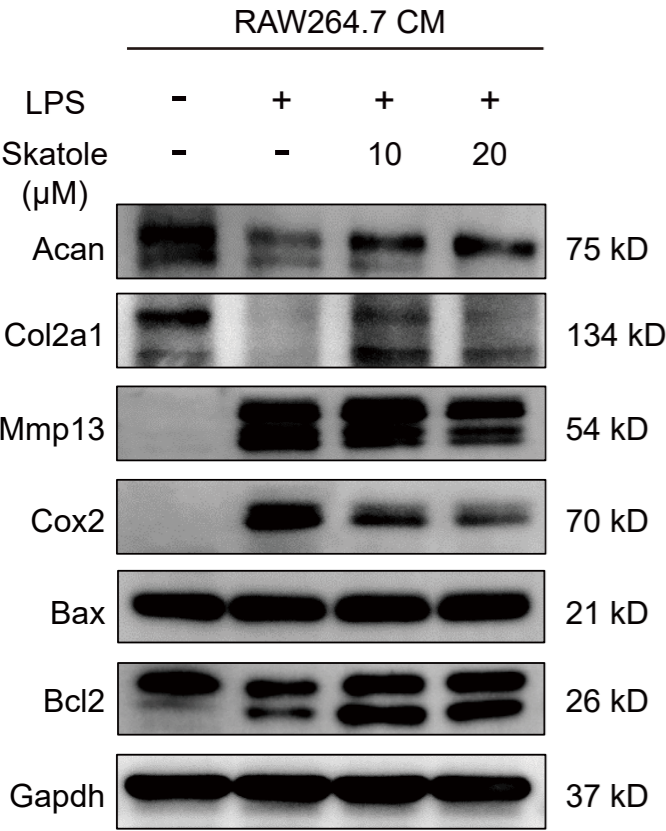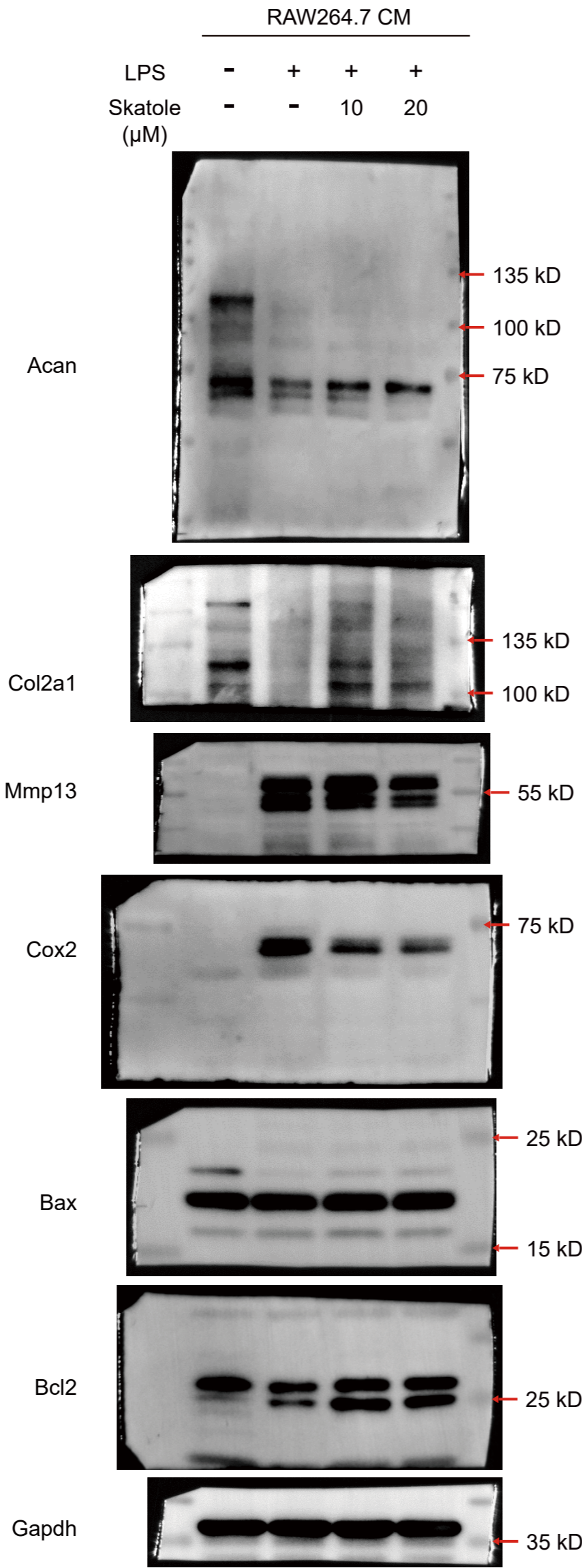

Fig 6J

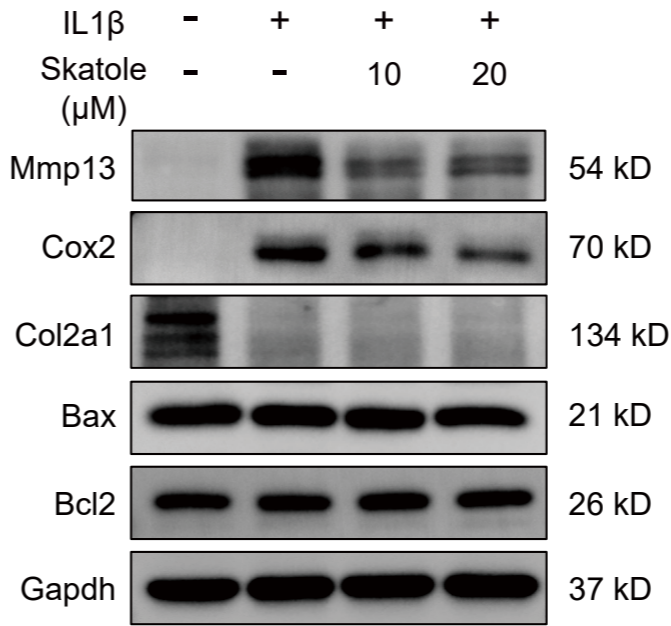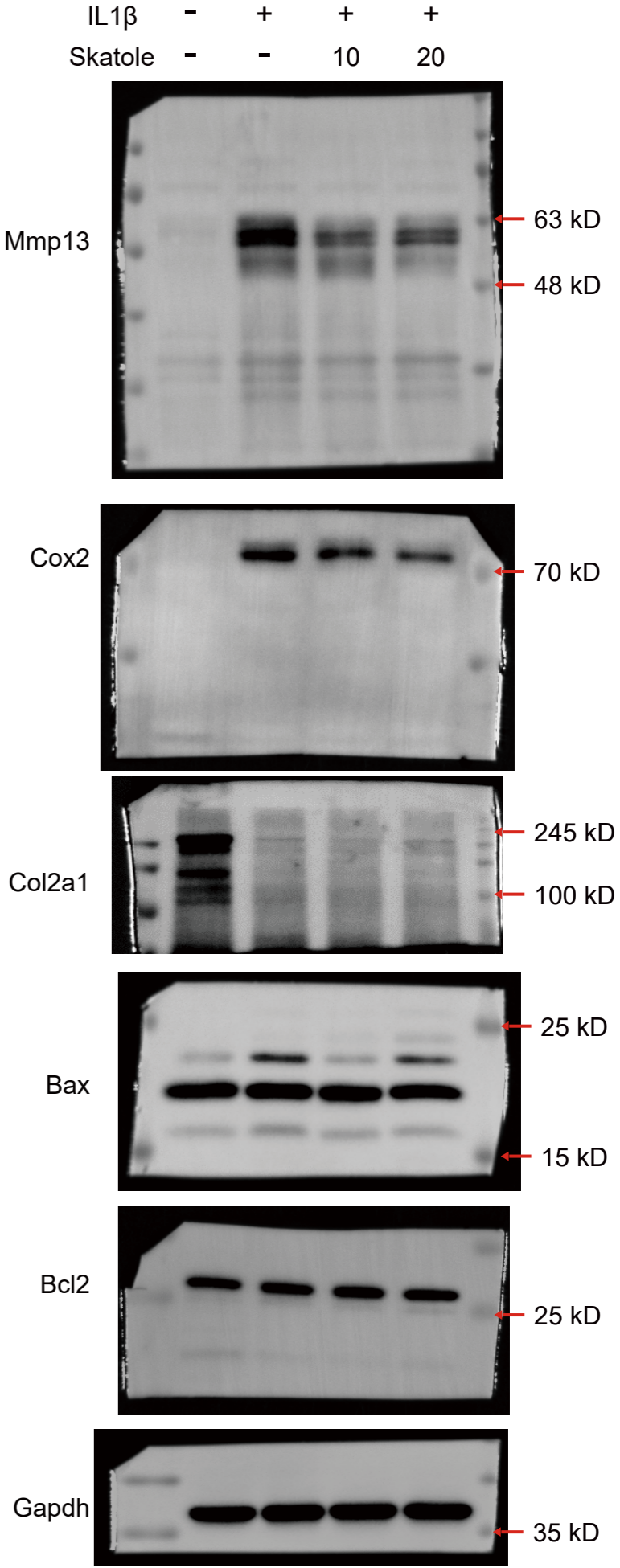

Supplement: Supplementary 1 — Figs. S1 to S13 Tables S1 and S2 [file research.0604.f1.zip › Fig S13.pdf]

BMDM cells

**A**

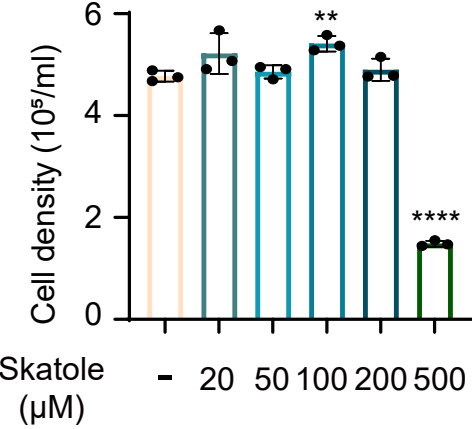

**C**

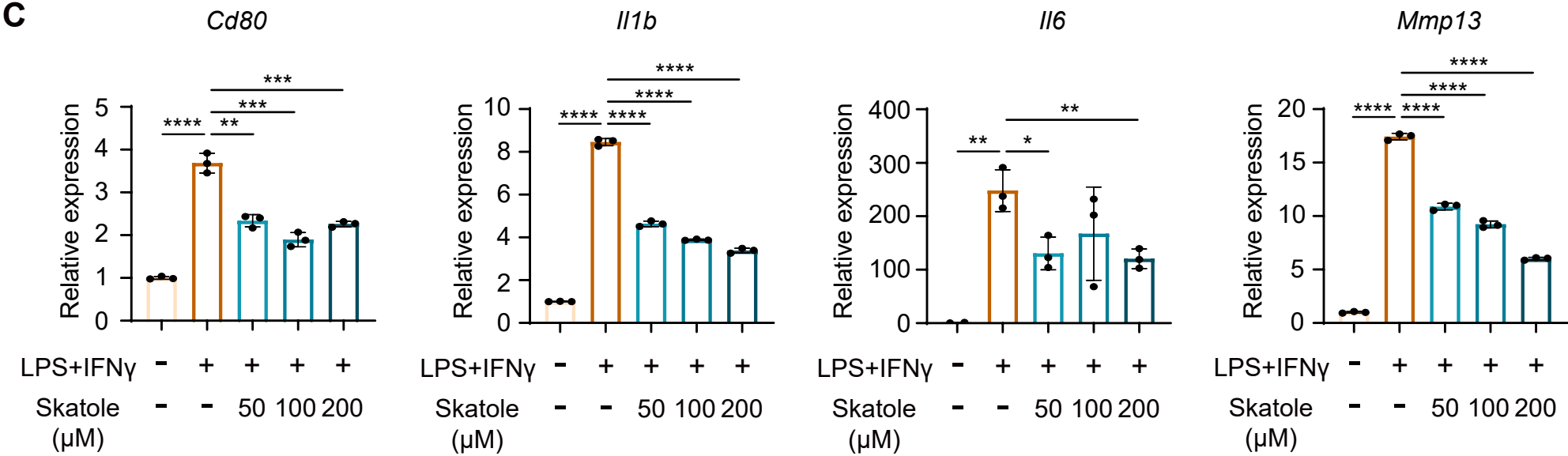

**B**

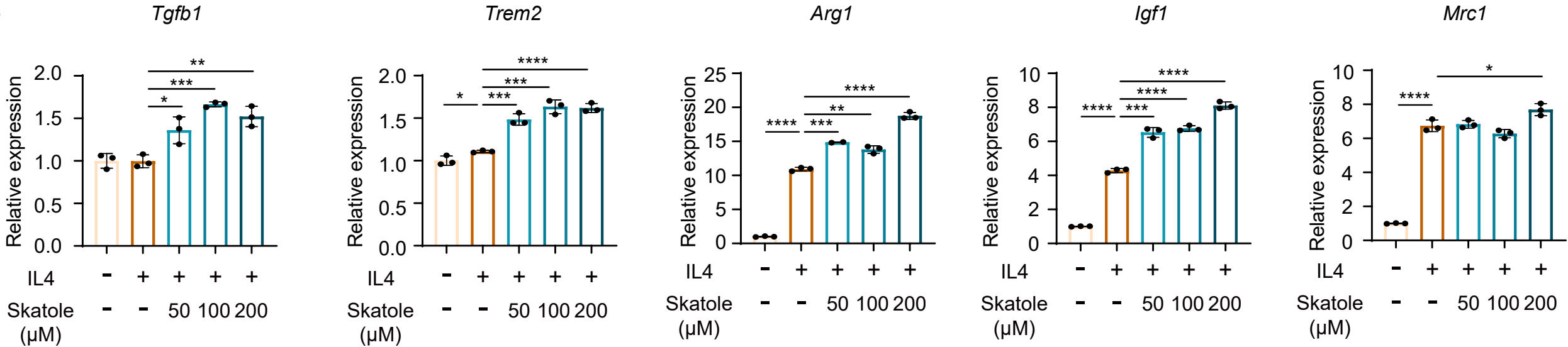

Supplement: Supplementary 1 — Figs. S1 to S13 Tables S1 and S2 [file research.0604.f1.zip › Fig S2.pdf]

**A***Cd86*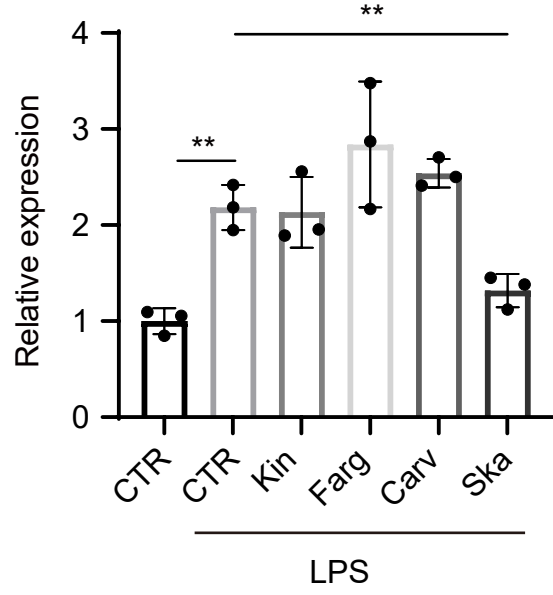*Il1b*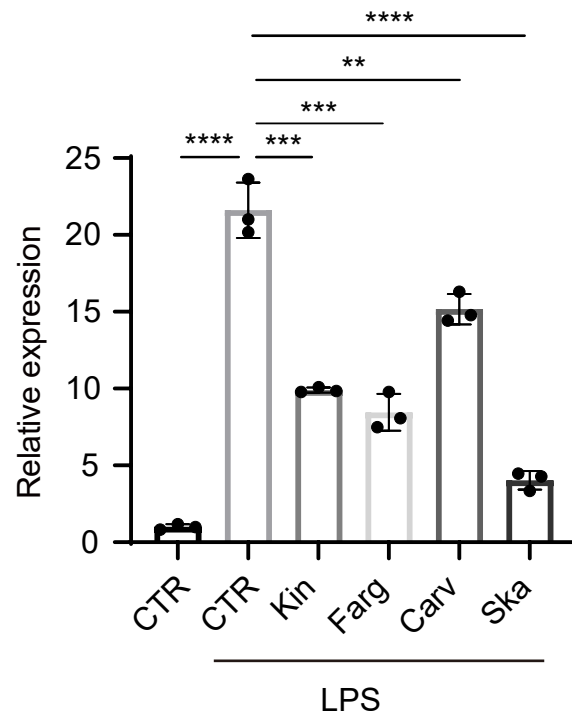*Il6*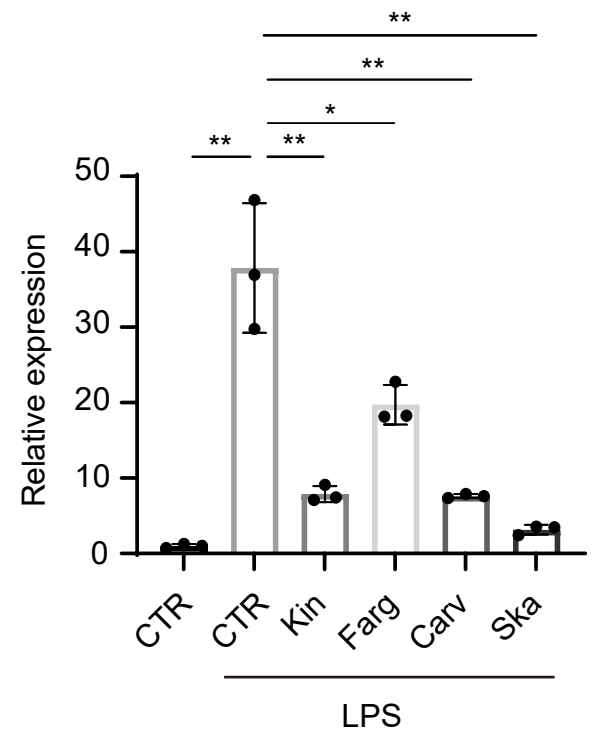**B***Mrc1*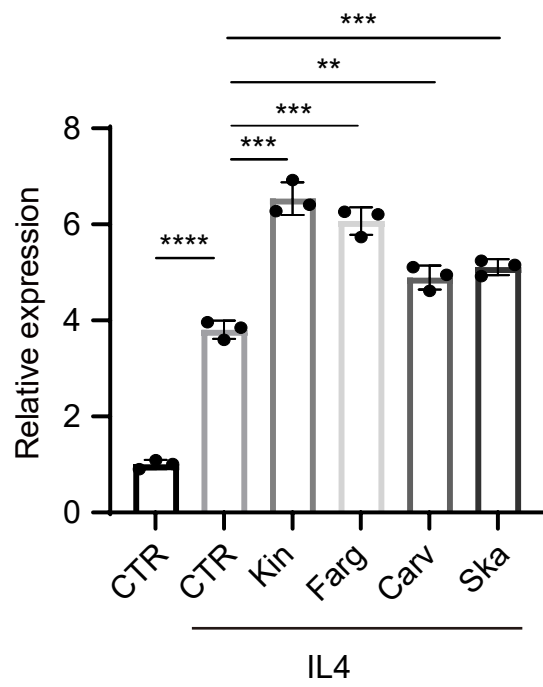*Arg1*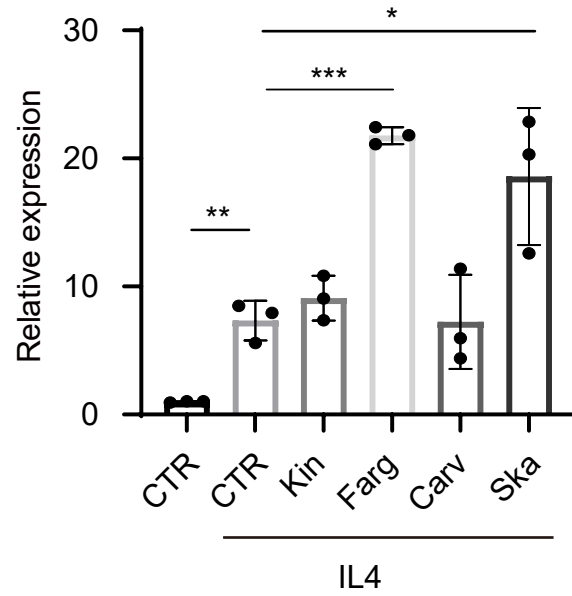*IL10*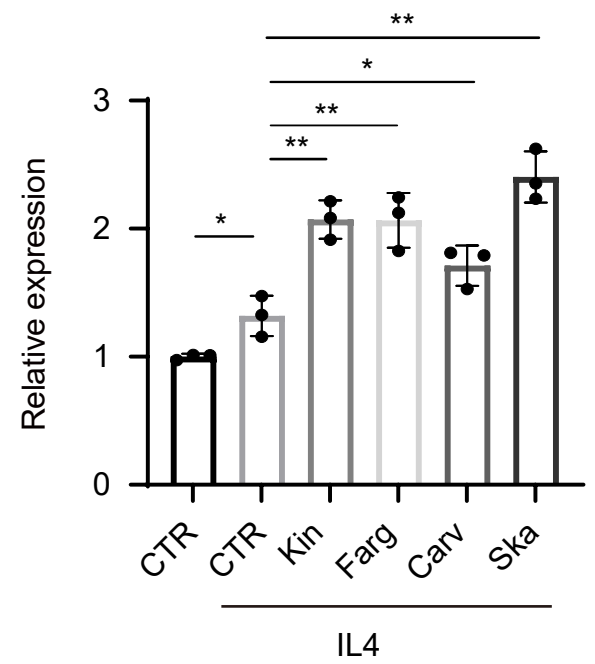

Supplement: Supplementary 1 — Figs. S1 to S13 Tables S1 and S2 [file research.0604.f1.zip › Fig S3.pdf]

**A**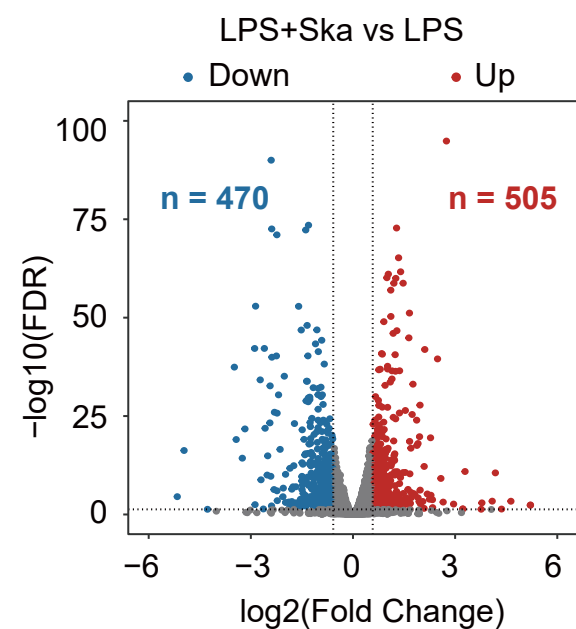**B**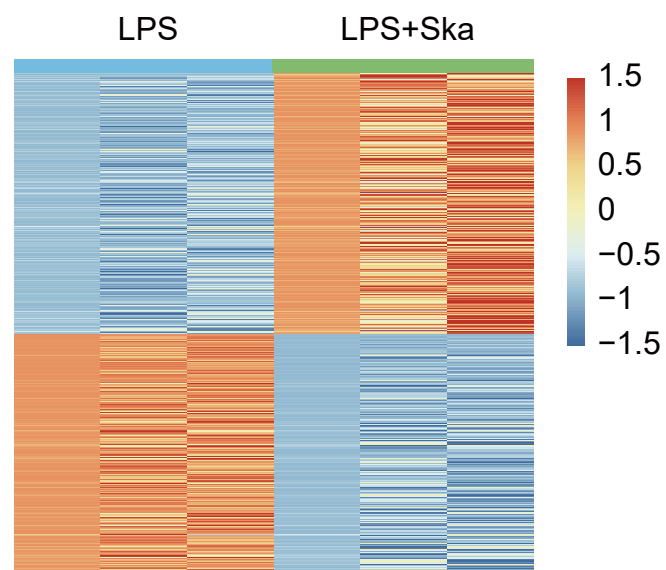**C**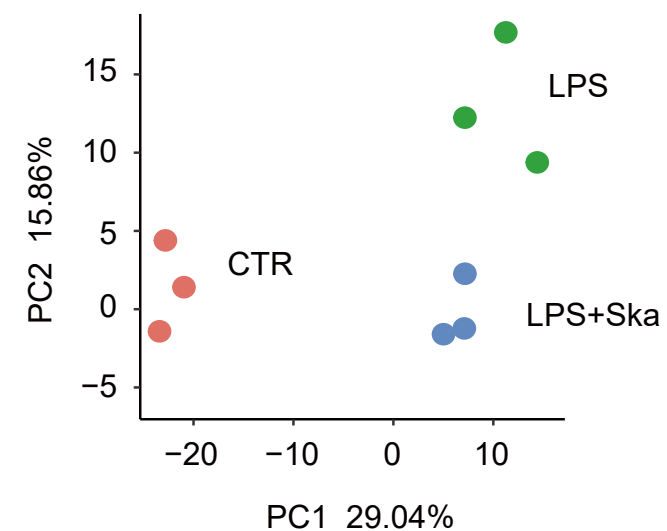**D**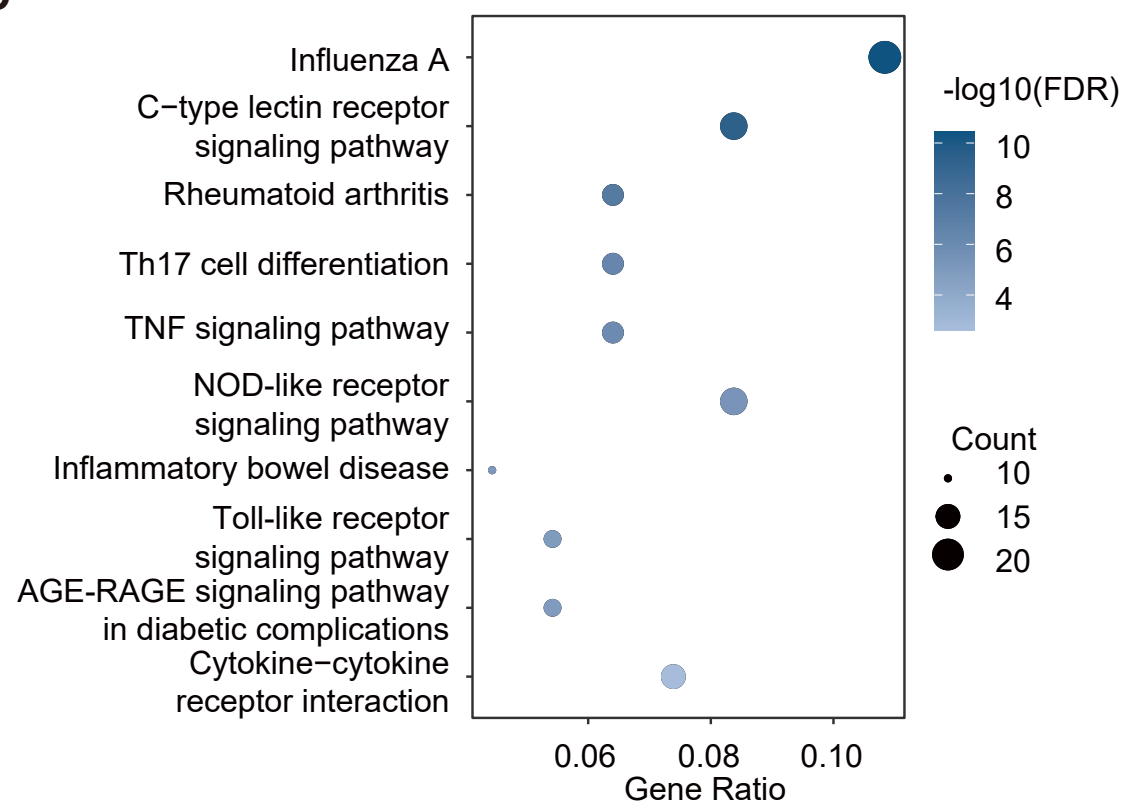**F**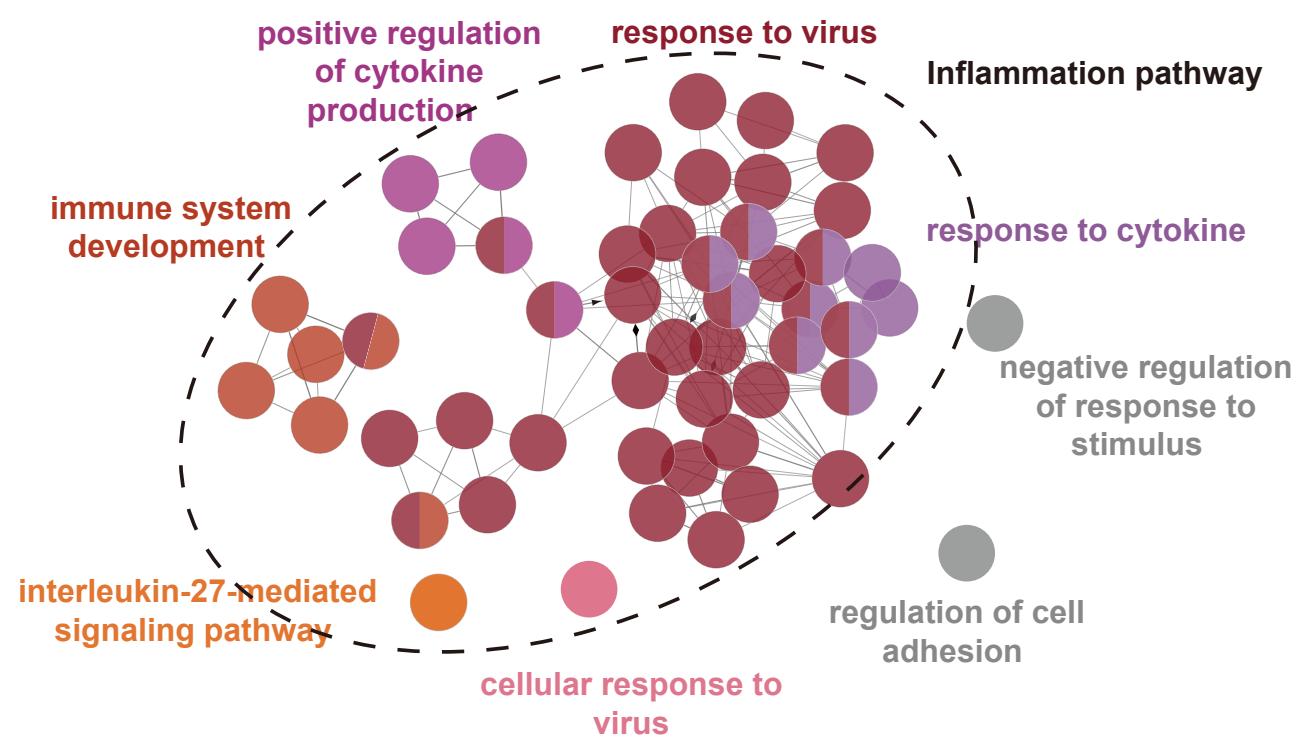**E**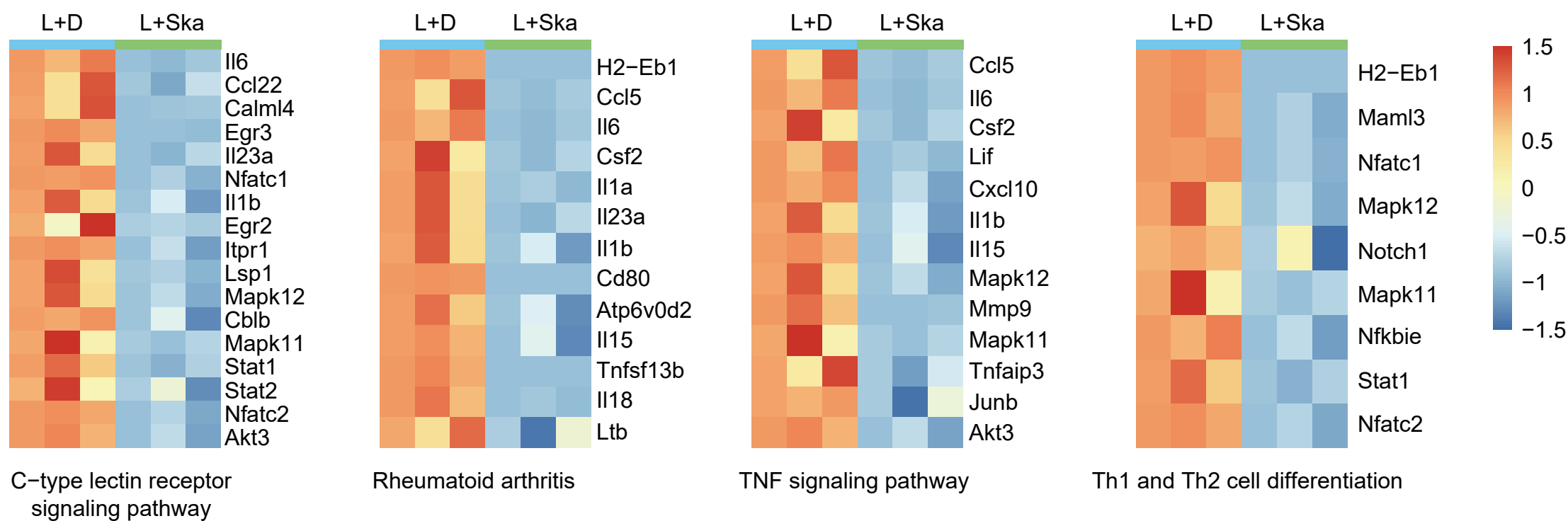

Supplement: Supplementary 1 — Figs. S1 to S13 Tables S1 and S2 [file research.0604.f1.zip › Fig S4.pdf]

**A**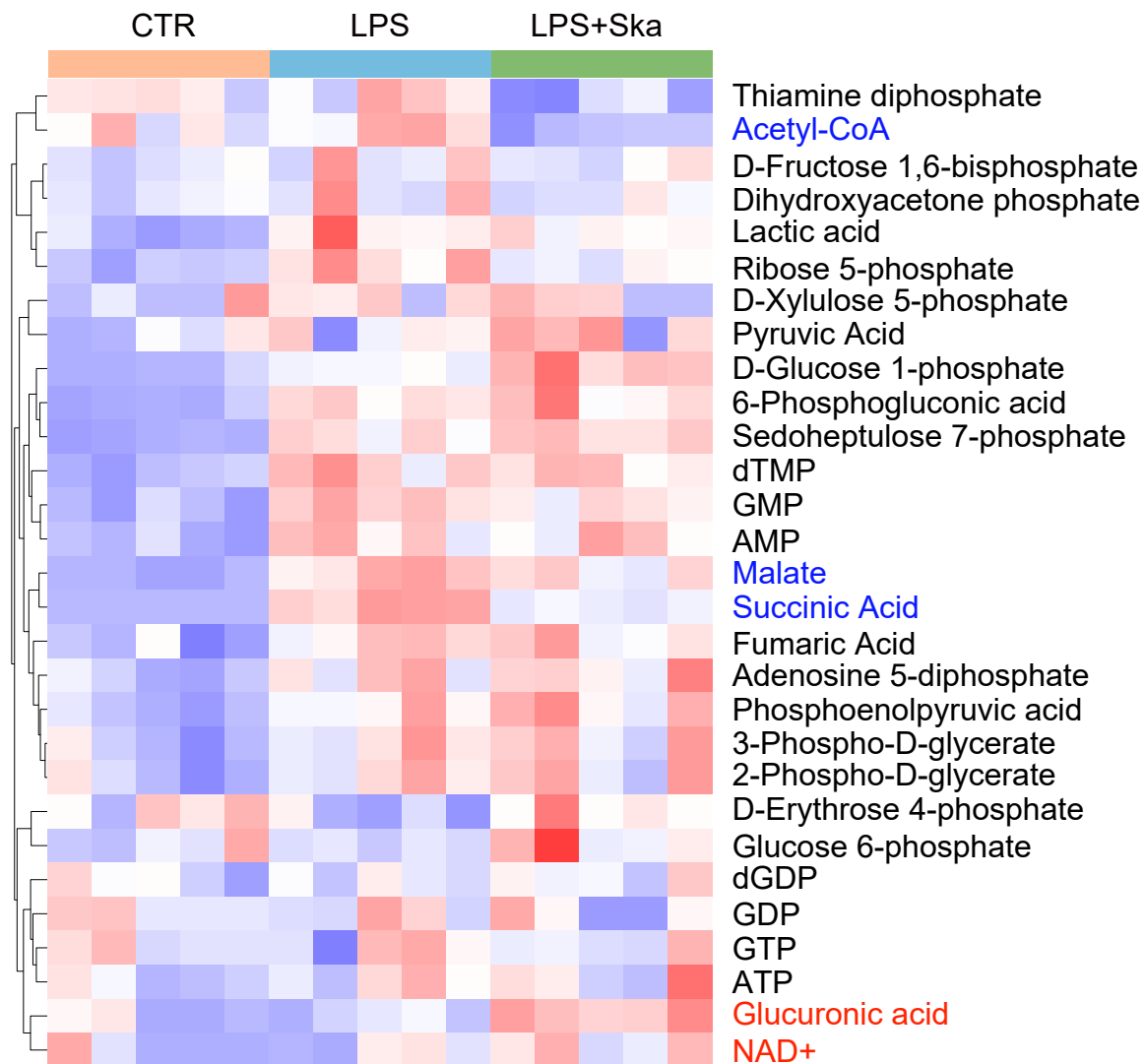**B**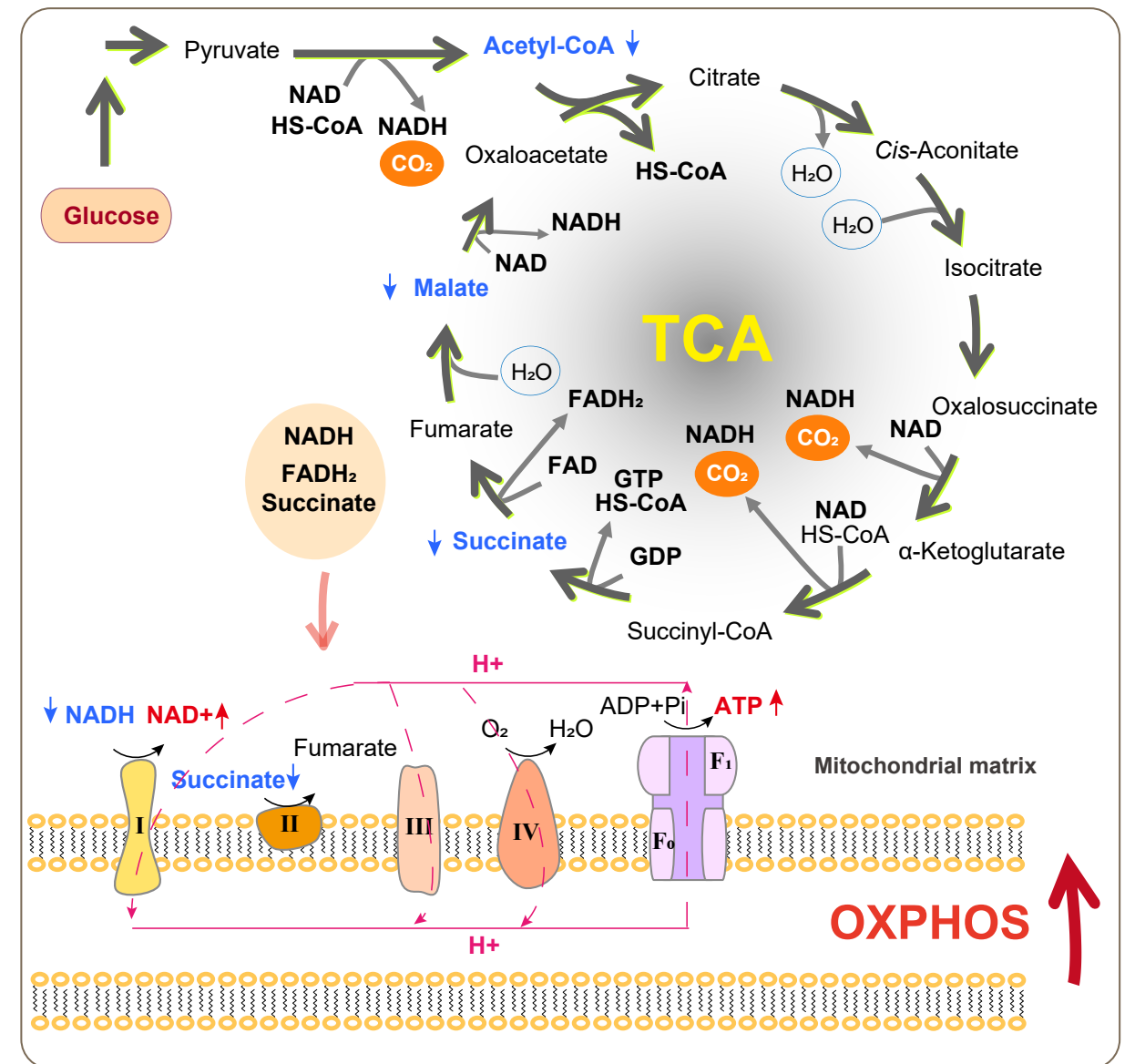**C**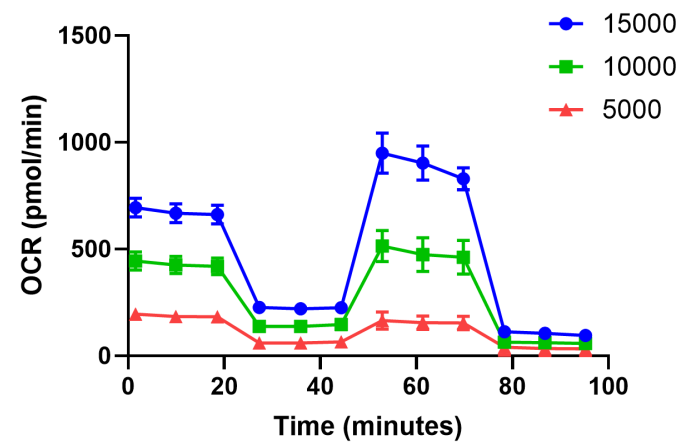**D**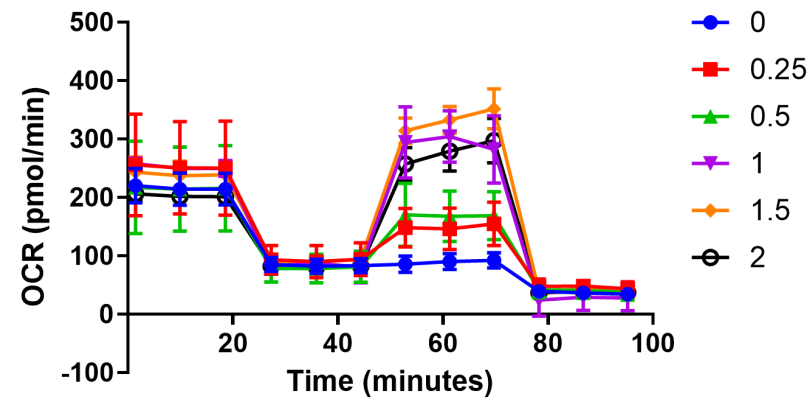

Supplement: Supplementary 1 — Figs. S1 to S13 Tables S1 and S2 [file research.0604.f1.zip › Fig S5.pdf]

# ATDC5 cell line

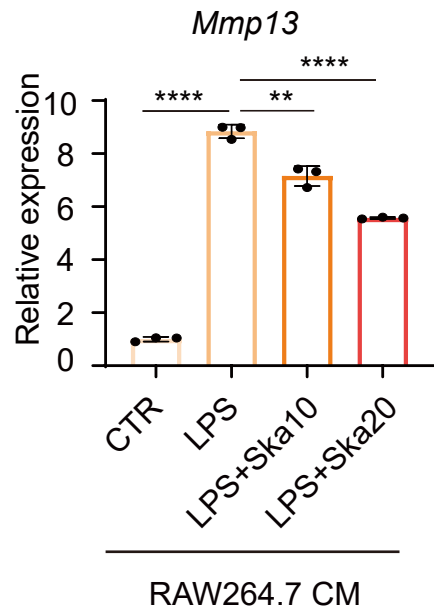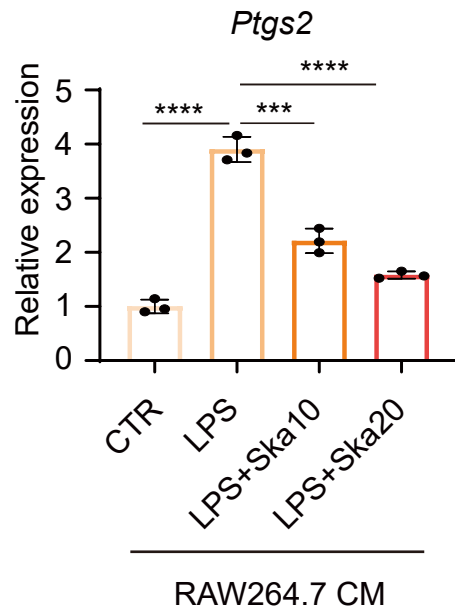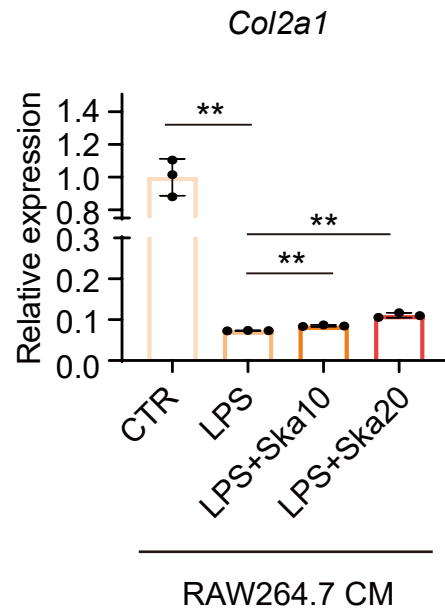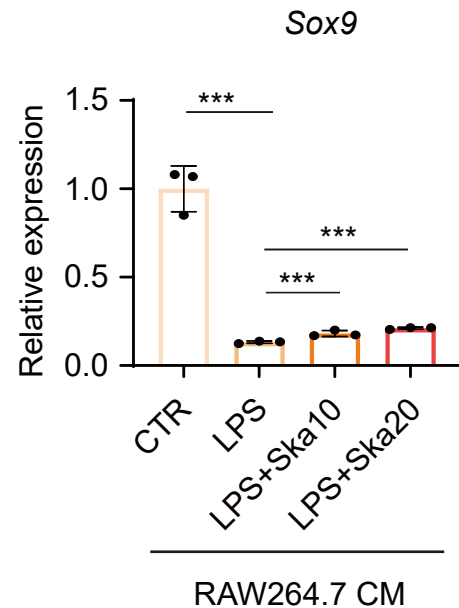

Supplement: Supplementary 1 — Figs. S1 to S13 Tables S1 and S2 [file research.0604.f1.zip › Fig S6.pdf]

# *Cd80*

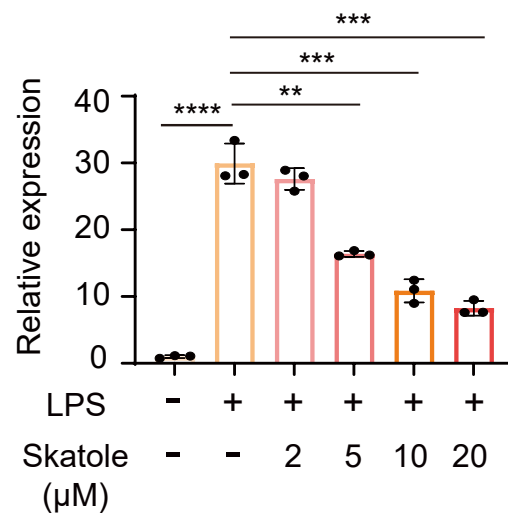

Supplement: Supplementary 1 — Figs. S1 to S13 Tables S1 and S2 [file research.0604.f1.zip › Fig S7.pdf]

**A**

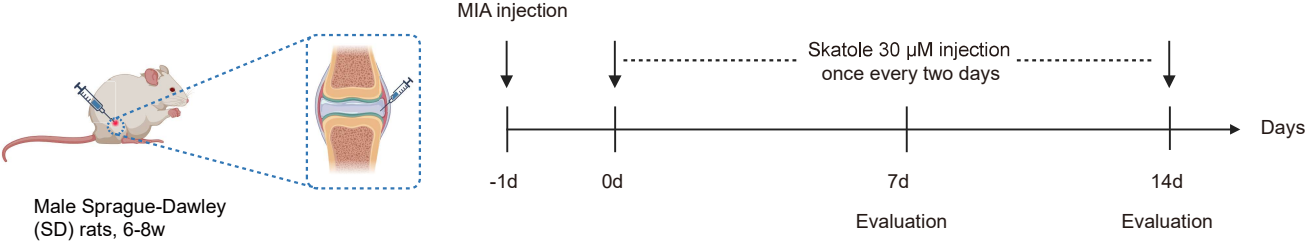

**B**

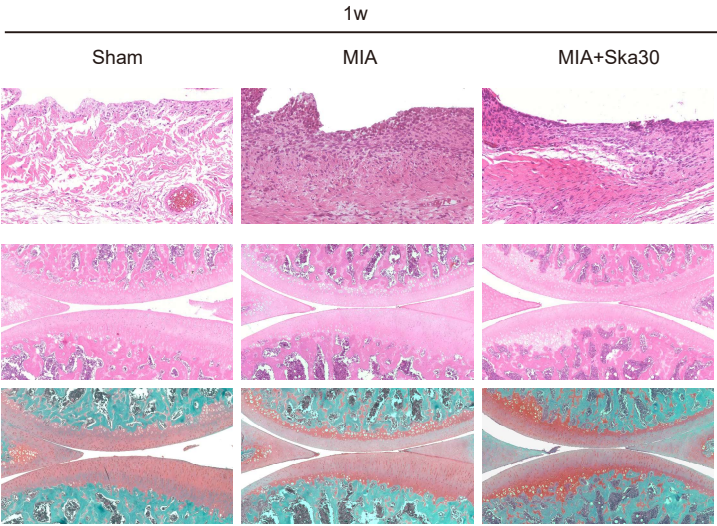

**C**

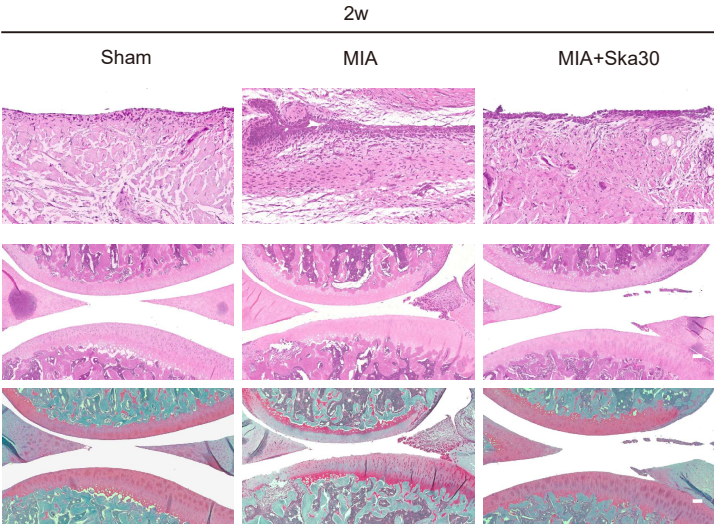

Supplement: Supplementary 1 — Figs. S1 to S13 Tables S1 and S2 [file research.0604.f1.zip › Fig S8.pdf]

Normal joint    OA joint

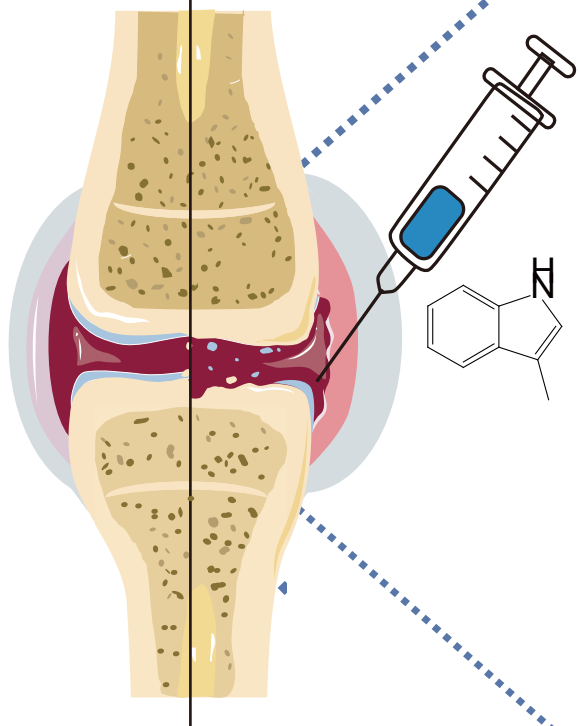

**Skatole**

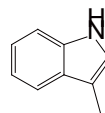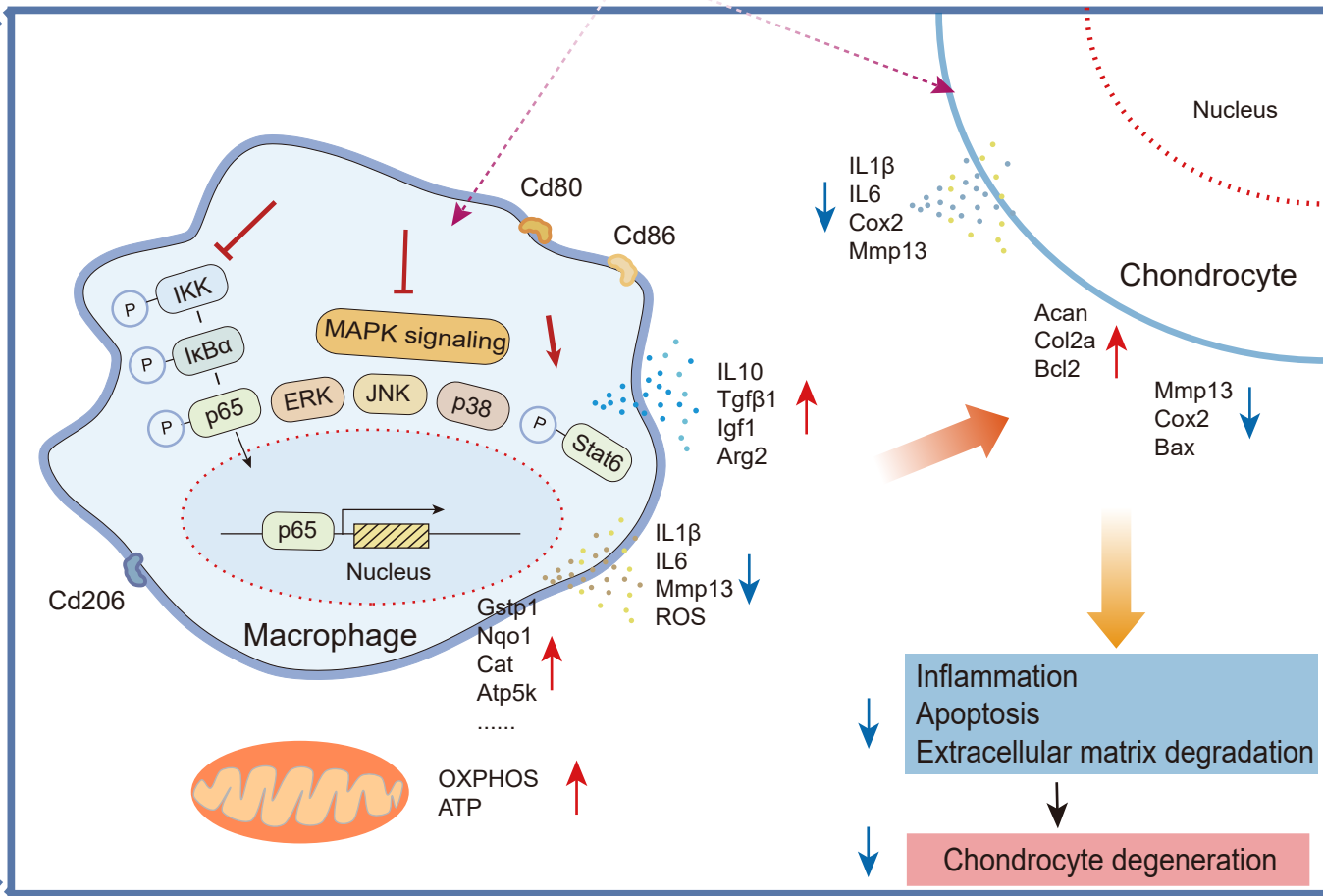

Supplement: Supplementary 1 — Figs. S1 to S13 Tables S1 and S2 [file research.0604.f1.zip › Fig S9.pdf]
